# Assessment of the Effects of MPTP and Paraquat on Dopaminergic Neurons and Microglia in the Substantia Nigra Pars Compacta of C57BL/6 Mice

**DOI:** 10.1371/journal.pone.0164094

**Published:** 2016-10-27

**Authors:** Richard Jay Smeyne, Charles B. Breckenridge, Melissa Beck, Yun Jiao, Mark T. Butt, Jeffrey C. Wolf, Dan Zadory, Daniel J. Minnema, Nicholas C. Sturgess, Kim Z. Travis, Andrew R. Cook, Lewis L. Smith, Philip A. Botham

**Affiliations:** 1 St. Jude Children’s Research Hospital, Dept. of Developmental Neurobiology, 262 Danny Thomas Place, Memphis, TN 38105, United States of America; 2 Syngenta Crop Protection, LLC, P.O. Box 18300, Greensboro, NC 27419–8300, United States of America; 3 WIL Research Laboratories, LLC., Ashland, OH 44805, United States of America; 4 Tox Path Specialists, LLC, 8747 Chestnut Grove Road, Frederick, MD 21701–2607, United States of America; 5 Experimental Pathology Laboratories, Inc., 45600 Terminal Drive, Sterling, VA 20166, United States of America; 6 Syngenta Limited, Jealott's Hill International Research Centre, Bracknell, Berkshire, RG42 6EY, United Kingdom; 7 University of Leicester, University Road, Leicester LE1 7RH, United Kingdom; Emory University, UNITED STATES

## Abstract

The neurotoxicity of paraquat dichloride (PQ) was assessed in two inbred strains of 9- or 16-week old male C57BL/6 mice housed in two different laboratories and compared to the effects of 1-methyl-4-phenyl-1,2,3,6-tetrahydropyridine (MPTP). PQ was administered by intraperitoneal injections; either once (20 mg/kg) or twice (10 mg/kg) weekly for 3 weeks, while MPTP-HCl was injected 4 times on a single day (20 mg/kg/dose). Brains were collected 8, 16, 24, 48, 96 or 168 hours after the last PQ treatment, and 48 or 168 hours after MPTP treatment. Dopamine neurons in the substantia nigra pars compacta (SNpc) were identified by antibodies to tyrosine hydroxylase (TH^+^) and microglia were identified using Iba-1 immunoreactivity. The total number of TH^+^ neurons and the number of resting and activated microglia in the SNpc at 168 hours after the last dose were estimated using model- or design-based stereology, with investigators blinded to treatment. In a further analysis, a pathologist, also blinded to treatment, evaluated the SNpc and/or striatum for loss of TH^+^ neurons (SNpc) or terminals (striatum), cell death (as indicated by amino cupric silver uptake, TUNEL and/or caspase 3 staining) and neuroinflammation (as indicated by Iba-1 and/or GFAP staining). PQ, administered either once or twice weekly to 9- or 16-week old mice from two suppliers, had no effect on the number of TH^+^ neurons or microglia in the SNpc, as assessed by two groups, each blinded to treatment, using different stereological methods. PQ did not induce neuronal cell loss or degeneration in the SNpc or striatum. Additionally, there was no evidence of apoptosis, microgliosis or astrogliosis. In MPTP-treated mice, the number of TH^+^ neurons in the SNpc was significantly decreased and the number of activated microglia increased. Histopathological assessment found degenerating neurons/terminals in the SNpc and striatum but no evidence of apoptotic cell death. MPTP activated microglia in the SNpc and increased the number of astrocytes in the SNpc and striatum.

## Introduction

Paraquat (1,1'-dimethyl-4,4'-bipyridinium dichloride) [PQ] is a non-selective herbicide that interferes with photosynthesis (photosystem I) and damages plant membrane proteins by producing oxygen free radicals. The PQ ion acts as a redox cycling agent by accepting an electron from reducing equivalents from chloroplasts (plants) or NADPH/NADH (animals) to form a PQ radical. Provided oxygen is present, the additional electron of the radical is passed onto molecular oxygen to form a superoxide radical with the concomitant regeneration of the paraquat ion [1–3]. The generation of sufficient quantities of superoxide will lead to the formation of hydrogen peroxide and a cascade of reactions leading to lipid peroxidation that causes cell damage and death. Furthermore, this continual redox cycling of PQ between the oxidized and radical form, depletes cellular reducing equivalents (such as NADPH/NADH) to cause the inhibition of vital physiological and biochemical activity. Due to paraquat’s selective uptake by the lung, this organ is the primary target organ associated with paraquat poisoning in humans [4, 5]. It has been reported that PQ can cross the blood brain barrier [6], possibly via neutral amino acid transporters [7] or the organic cation transporters, OCT-2 and OCT-3 [8, 9]. While it has been postulated that PQ is taken up into dopaminergic neurons by the dopamine transporter (DAT), the divalent paraquat ion does not appear to be a substrate for DAT [10, 11].

Some epidemiological studies have found significant associations between PQ use and Parkinson’s disease (PD) in agricultural workers [12–14], while others have not [15, 16]. These results have been reviewed by Mandel et al., [17] in regard to epidemiology studies and Breckenridge et al. [18], who conducted a weight of the evidence assessment.

A number of studies in C57BL/6 male mice have shown that intraperitoneal (ip) injections of PQ reduced the number of tyrosine-hydroxylase-positive (TH^+^) neurons in the substantia nigra pars compacta (SNpc) [19–21]. However, others have found no effect of PQ on TH^+^ neurons in the SNpc, and no evidence of neurodegeneration in the SNpc or striatum [22].

The purpose of this study was to investigate the potential basis for differences in the effects of PQ on the SNpc reported in different laboratories [21–23] by conducting controlled, multi-site experiments that systematically varied PQ dose and dose frequency, the source and age of the C57BL/6 male mice, animal housing conditions and stereological and neuropathological methods. The in-life phases of these studies were conducted in two laboratories. Stereology was performed by two investigators who were blinded to treatment, and the neuropathological evaluations were conducted by another investigator, also blinded to treatment.

In the current study in was found that, unlike the neurotoxin 1-methyl-4-phenyl-1,2,3,6-tetrahydropyridine (MPTP), [24], PQ did not induce a loss of SNpc dopaminergic (DA) neurons, did not cause damage to DA axons/terminals or produce evidence of neuroinflammation. This was true irrespective of PQ dose or dose frequency, the source of PQ, the source, age or husbandry of the C57BL/6J male mice evaluated, and the stereological or neuropathological methods employed. The results were robust and consistent among the three investigators.

## Materials and Methods

### Animals

Male C57BL/6J mice were supplied by Jackson Laboratories (JAX) (Bar Harbor, ME) and male C57BL/6NHsd mice were obtained from Harlan Laboratories (Indianapolis, IN). Depending on the study, the mice were approximately 3 to 6 weeks of age upon receipt and were housed in either a designated animal room of the AAALAC accredited WIL Research Laboratories, LLC, Ashland, Ohio (C57BL/6J mice), or under maximum barrier conditions (Jackson Laboratories and Harlan mice) at St. Jude Children’s Research Hospital (SJCRH, Memphis, TN). The experiment conducted at WIL was performed according to Good Laboratory Practice and the protocol (WIL 639193) was approved by the Institutional Animal Care and Use committees at WIL. The experiments at SJCRH were performed in accordance with the NIH Guide for the Care and Use of Laboratory Animals, and all protocols were approved by the Saint Jude Children's Research Hospital IACUC. Experiments were carried out in accordance with The Code of Ethics of the World Medical Association (Declaration of Helsinki, *2013*) for animal experimentation.

Mice were single-housed at WIL in stainless-steel, wire-mesh-floored cages on a daily 12:12 light-dark cycle with room temperature and relative humidity maintained at 22°C ± 3°C and 50% ± 20%, respectively. For the first 24 hrs after MPTP treatment, mice were housed in plastic shoe-box cages with Bed-O’Cobs nesting material and provided with supplemental heating pads. Mouse chow (PMI Nutrition International, LLC Certified Rodent LabDiet^®^ 5002) and municipal water were provided *ad libitum*. At SJCRH, mice were housed 5/cage in polycarbonate shoebox cages with corn cob bedding, on a daily 12:12 light-dark cycle with room temperature and relative humidity maintained at 23 ± 1°C. Mouse chow (LabDiet 5013, Purina) and reverse-osmosis (RO) treated water were provided *ad libitum*.

Prior to all studies, mice underwent a 3–6 week quarantine period during which they were observed twice daily for mortality, as well as for changes in general appearance or behavior. At the end of the quarantine period, the mice were assigned to control and treatment groups using a randomized block design, stratified by weight and age (Table 1).

**Table 1:** Number, design and location of studies examining effects of paraquat and MPTP on C57BL/6J and C57BL/6NHsd male mice (aged 9 or 16 weeks)^1^.

Parameter	SJCRH: Model-BasedStereology	EPL:Design-Based Stereology	TPS:Neuropathology
**Experiment 1a: C57BL/6J Males: 9-Week Old at 1^st^ PQ Dose (WIL)**			
Vehicle Controls ^2^	**14**	**20**	**50**
PQ (10 mg/kg x 6 doses)	**14**	**18**	**50**
PQ (20 mg/kg x 3 doses)	**13**	**20**	**50**
MPTP (20 mg/kg x 4 doses)	**10**	**8**	**5**
**Experiment 1b: C57BL/6J Males: 16-Week Old at 1**^**st**^ **PQ Dose (WIL)**			
	**SJCRH: Model-Based Stereologyof DA Neurons in the SNpc**	**SJCRH: Model-Based Stereology of Resting and Active Microglia in the SNpc**	
**C57BL/6J Mice**			
Vehicle Controls ^2^	**19**	**19**	
PQ (10 mg/kg x 6 doses)	**19**	**19**	
MPTP (20 mg/kg x 4 doses)	**10**	**10**	
**C57BL/6NHsd Mice**			
Vehicle Controls	**10**	**10**	
PQ (10 mg/kg x 6 doses)	**13**	**12**	
MPTP (20 mg/kg x 4 doses)	**14**	**13**	

^1^10 mice/group were administered MPTP. Fewer mice were examined due to tissue damage during processing.

^**2**^ The vehicle control group was 9 weeks of age at the initiation of dosing in the WIL study and 16 weeks of age at the initiation of dosing in the SJCRH study. Statistical comparisons of treated groups were to the appropriate aged controls.

### Experimental Design

The purpose of Experiment 1 (Table 1) was to assess the effect of paraquat on the total number of TH^+^ neurons in the SNpc in male C57BL/6 mice of two ages (9- or 16-weeks) with different dose levels and dose frequency (1 or 2 doses/week); via two different stereological assessment methods (design or model-based stereology); and employing semi-quantitative neurohistopathological assessments (TH^+^ staining reduction, neurodegeneration, apoptosis or gliosis). The stereology was performed at St. Jude Children’s Research Hospital (SJCRH) and Experimental Pathology Laboratories, Inc (EPL, Sterling VA), while the qualitative neuropathology was performed at Tox Path Specialists LLC (TPS, Frederick MD).

Due to the large number of groups of mice evaluated, age effects were assessed by comparing 9-week old PQ and MPTP-treated animals (Experiment 1a) to 16-week old mice (Experiment 1b). Sixteen week old mice in the WIL study (Experiment 1b) were also statistically compared to 16-week old control mice in the study conducted at SJCRH (Experiment 2) and 16-week old PQ and MPTP groups at WIL (Experiment 1b) were compared to the corresponding 16-week old PQ and MPTP groups at SJCRH (Experiment 2, TH^+^ stereology only).

### Preparation of PQ and MPTP Dose Formulations

#### Experiment 1 (G1 to G3; WIL)

MPTP hydrochloride, a 100% pure fine white powder, was obtained by WIL from Sigma-Aldrich, Inc., St. Louis, MO (Lot #128K1549), and stored at room temperature. Paraquat dichloride monohydrate (99.7% pure) was supplied by Syngenta (Batch ID 550055) and kept desiccated at 2°C to 8°C. All materials were used within the period defined by their respective expiration dates. Both MPTP-HCl (7.75 mg/mL, or 6.4 mg/L as the free base) and PQ (1.0 or 2.0 mg/mL, expressed as the dichloride salt) were formulated as solutions in 0.9% sodium chloride (USP). All PQ dose formulations, verified by HPCL prior to dose administration as described previously [22](S1 Appendix), were homogeneous, stable, and within ± 10% of target concentration.

#### Experiment 2 (G4-G5; SJCRH)

MPTP hydrochloride was obtained by SJCRH from Sigma-Aldrich, Inc. (St. Louis, MO, Catalog #M0896, Lot #SLBH6218V), and stored at room temperature. Paraquat dichloride monohydrate was supplied by Sigma-Aldrich (CAS Number 75365-73-0, Lot #SZBB348XV) and kept desiccated at 2°C to 8°C. Both MPTP-HCl (5.0 mg/mL, or 4.1 mg/mL expressed as the free base) and PQ (1.2 and 2.4 mg/mL, expressed as the dichloride salt, for the C57BL/6J and the C57BL/6NHsd mice, respectively) were formulated as solutions in 0.9% sodium chloride (USP). All PQ dose formulations were verified by HPCL prior to dose administration as described previously [22].

### Administration of PQ and MPTP

Mice were weighed immediately prior to injection. PQ was administered by ip injection twice weekly at a dose of 10 mg PQ (salt)/kg/dose for 3 weeks (60 mg/kg total in 6 injections), or once weekly at 20 mg PQ (salt)/kg/dose for 3 weeks (60 mg/kg in 3 injections). PQ dose volumes were 10 mL/kg/dose at WIL, 8.3 mL/kg/dose for the C57BL/6J mice at SJCRH, and 4.3 mL/kg/dose for the C57BL/6NHsd mice at SJCRH.

Mice administered MPTP·HCl (19.4 mg/kg/dose at WIL; 20 mg/kg/dose at SJCRH) were weighed immediately prior to the first injection, and then injected (ip) at 2 hour intervals for a total of 4 doses (total dose of 80 mg/kg). MPTP dose volumes were 2.5 mL/kg/dose at WIL and 4 mL/kg/dose at SJCRH. Shoebox bin cages housing MPTP-treated animals housed at both SJCRH and WIL were placed on heating pads for 24 hours after dosing in order to maintain animal viability [25].

Control mice were administered 0.9% saline at the same volume as the PQ mice.

### Brain PQ Concentration

To quantitate brain levels of PQ, five C57BL/6J mice at SJCRH were sacrificed 24 hours after the final (sixth) PQ dose (10 mg/kg/dose) and brains, excluding olfactory bulbs, were removed and frozen samples were shipped on dry ice to Charles River Laboratory, Tranent, Edinburgh, UK, where they were homogenized and analyzed for PQ concentration using LC-MS/MS (S1 Appendix). Group mean concentration and the relative standard deviation (CV) were calculated.

### Estimation of the number of TH^+^ neurons in the SNpc

Two stereological methods were utilized to estimate the total number of TH^+^ (DA) neurons in the SNpc: Model-Based and Design-Based stereology [26]. Mice were deeply anesthetized with sodium pentobarbital (WIL) or Avertin (SJCRH) until all deep tendon and corneal reflexes were absent. Mice were then perfused transcardially with buffered physiological saline followed by 4% (WIL) or 3% (SJCRH) paraformaldehyde. Brains from the WIL-treated mice were sent either to Experimental Pathology Laboratories (EPL, Sterling VA) or SJCRH for analysis, whereas mice-treated at SJCRH were processed on site. All of the stereological evaluations were performed blinded to treatment group at both EPL and SJCRH.

### Model-based stereology

**Fig 1:**
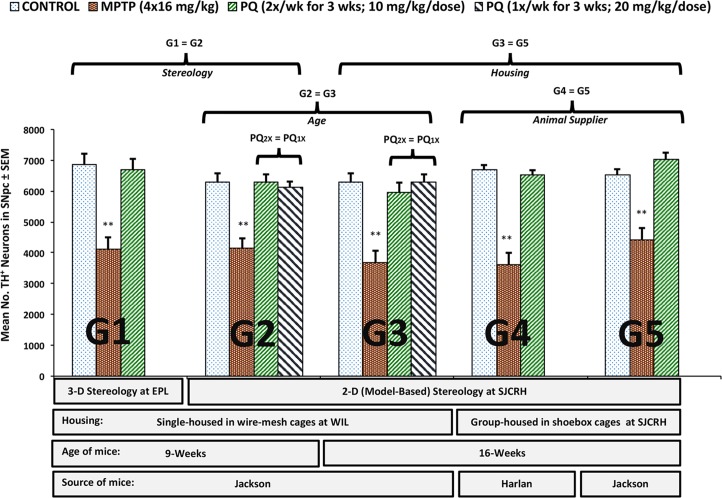
Stereological assessment of the mean number of TH^+^ neurons in the SNpc following PQ or MPTP treatment in C57BL/6J and C57BL/6NHsd male mice. Five different groups (G1-G5) of animals, varying in age and site of experiment were injected with saline, paraquat or MPTP and the extent of TH^+^ neuron loss was assessed by design-based or model-based stereology. ** significantly different from control mice (p ≤ 0.01). Syngenta-sourced PQ was used to treat mice G1 to G3 mice, whereas Sigma Chemical PQ was used to treat mice in groups G4 and G5.

The model-based (thin section, 2-dimensional (2D)) stereological evaluations were carried out at SJCRH with the investigator (RJS) blinded to treatment. After perfusion, brains were removed from the calvaria and post-fixed in the corresponding fixatives and processed for stereological analysis as previously described [26]. Briefly, each brain was blocked in paraffin and serially sectioned at 10 μm from the rostral hippocampus to the anterior aspects of the cerebellar–midbrain junction, thereby including the rostral and caudal extent of the SNpc. Five 10 μm sections were mounted onto an individual slide and every other slide was immunostained for TH (mouse monoclonal TH; Sigma-Aldrich; 1:500) and visualized using 3,3’-diaminobenzidine (DAB). From these slides, the 3^rd^ section was selected for analysis, so that sampling was at 100 μm intervals. After staining, the anterior to posterior extent of the SN (See Fig 1 Baquet *et al*., 2009) [26] was identified based on a standard mouse brain atlas [27] using landmarks shown in Nelson et al. [28]. The rostral portion of the SNpc starts with the first TH^+^ cells located near the end of the subthalamic nucleus and lateral to the TH^+^ stained fibers in the medial forebrain bundle. The caudal SNpc ends where the retrorubral field becomes visible. The outlines used include the substantia nigra lateralis. The dorsal portion of the SN pars reticulata defined the ventrolateral boundary. The anterior medial boundary is defined by the ventral tegmental area and by size and orientation of stained cells. DA neurons of the SNpc are larger than ventral tegmental area DA neurons [28], and SNpc DA neurons orient along the long axis of the SNpc. The posterior medial portion of the SNpc is defined by the medial lemniscus.

For each animal, the number of TH^+^ neurons was counted separately for the left and right side of the brain using a 40x objective (total magnification 400x). Once a section was counted, the TH^+^ cell count was multiplied by 10 to correct for uncounted sections and corrected for split nuclei using the Abercrombie correction factor [29]. Here, section thickness was measured by focusing from the top to the bottom of at least 20 representative sections/brain (100x magnification). The average thickness (t) was then used to estimate the correction factor to be applied to the total number of TH^+^ neurons actually counted (Eq 1).

N=n(t÷(t+x))

where *N* equals the estimated cell number, *n* equals raw cells counts, t equals section thickness measured for each brain, and x equals the empirically determined counting particle size.

To correct for DA neuronal size (counting particle, x), we measured the largest diameter of DA neurons in the x-or y-axes in each of three sections at the rostral, intermediate and caudal boundaries of the SNpc (total number of cells was approximately 200 cells/animal/axis). These values were summed and a mean calculated. The mean was then used as the value in calculating the total number of neurons (N). The number of neurons in the right and left SNpc were summed to provide an estimate of the total number of TH^+^ neurons in the SNpc.

### Designed-based stereology

Design-based stereology (thick section, 3-dimensional [3D]) was carried out at EPL with the investigator (DZ) blinded to treatment. For this analysis, brain tissue was cryopreserved, frozen sectioned at 40 μm in the coronal plane from the rostral hippocampus to the anterior aspects of the cerebellar–midbrain junction. Every section was immunostained for tyrosine hydroxylase (polyclonal TH, EMB Millipore, Billerica, MA, 1:1000) using free floating protocols as described previously [22], with DAB as the visualizing chromogen. Estimates of the number of TH^+^ neurons in the SNpc were generated by first delineating the borders of the SNpc as described above. The number of SNpc TH^+^ (DA) neurons was then calculated using the optical fractionator probe (StereoInvestigator, version 8.11, MBF Bioscience, Williston, VT, USA), as previously described [26] with sampling parameters empirically determined using methods described by Slomianka and West [30]. Based on differential shrinkage and differences in TH^+^ neuron distribution throughout the section, cells were sampled through the entire thickness of the tissue using a 1 μm guard zone. The number of neurons in the right and left SNpc were summed to provide an estimate of the total number of TH^+^ neurons in the SNpc.

### Estimation of the total number of microglia in the SNpc

To estimate the number of microglia in the SNpc, the alternative unstained slides used for model-based TH^+^ cell number were double-immunostained for TH (as described above) and Iba-1 (polyclonal Iba-1, Wako Chemicals; 1:500). The numbers of resting and activated [31] Iba-1-positive microglia in the SNpc were estimated using the optical fractionator probe with the investigator blinded to treatment. Microglia were classified as activated if the cell body was visibly increased in diameter and the cell had shortened and thickened processes. Microglia were classified as resting if they had a small cell body (≤3μm) and longer, thinner processes [32]. The number of microglia in the right and left SNpc were summed to provide an estimate of the total number of resting and active microglia in the SNpc.

### Semi-quantitative neuropathological assessment at ToxPath Specialists

At WIL, 8, 16, 24, 48, 96 and 168 hours after the final dose of PQ, or 48 hours after MPTP dosing, mice were anesthetized with sodium pentobarbital and then perfused transcardially with 25 mL of sodium cacodylate buffer followed by 75 mL of sodium cacodylate-based 4% paraformaldehyde (methanol free) as previously described [22]. Vehicle controls were also euthanized and perfused at each of these post-dose time points. The fixed brains were shipped to Neuroscience Associates (www.neuroscienceassociates.com/stains.htm), where the brains were trimmed, sectioned and stained. A maximum of 25 brains were embedded in a gelatinous matrix (Multibrain1) by Neuroscience Associates Inc.

Twenty-five brains were assigned to the matrix in a block-random fashion, so that a block comprised of a vehicle control animal, an MPTP-treated animal and an animal from each PQ-treated group. The brain (caudate/putamen area) was sectioned in the transverse plane from a level rostral to the striatum (caudate/ putamen) caudally through the caudal extent of the SNpc at section thicknesses of 30 μm in the coronal plane. Seven serial sections were obtained at 12-section (360 μm) intervals. This block of tissue encompassed all of the structures defined as basal ganglia including the striatum, globus pallidus, SNpc, and subthalamic nuclei.

Serial sections were immuno/cytochemically stained as follows:

1. Tyrosine hydroxylase (TH), an enzyme involved in the synthesis of DA, was immune-histochemically labeled (TH^+^) to identify dopaminergic neurons and their neuronal processes (axons, dendrites and synaptic terminals).
2. Glial fibrillary acidic protein (GFAP) was immunohistochemically labeled to selectively identify protein filaments unique to activated astrocytes.
3. Ionized Calcium Binding Adaptor Molecule 1 (Iba-1), a protein expressed by activated microglia, was immunohistochemically labeled to detect microglial cell activation.
4. Amino Cupric Silver (AmCuAg) is a cytochemical stain that selectively stains the disintegrating elements of dead neuronal cell bodies and the neuronal processes (axons, dendrites and synaptic terminals). The dead cells/processes are labeled with black particles (silver) against a pale background, permitting the detection of single necrotic neurons.
5. Caspase 3 is an enzyme that is expressed by cells during apoptotic cell death. The caspase 3 cleavage product was immunochemically labeled to detect neurons that are in the processes of dying via an apoptotic mechanism. Only SNpc sections were evaluated for caspase 3.
6. Thionine is a general morphological stain that detects Nissl substance, thereby revealing nuclear details within a variety of cell types.
7. Terminal deoxynucleotidyl transferase dUTP nick end labeling (TUNEL) is a method for detecting DNA fragmentation. When used in combination with cleaved caspase 3 immuno-reactivity, it provides a sensitive indicator of apoptosis. Only sections of the SNpc were processed for TUNEL.

The processed slides were shipped to ToxPath Specialists (www.toxpath.net), where they were evaluated by the study pathologist (M.B.) who was blinded to treatment. A qualitative scoring system (S2 Appendix) was used to characterize the severity of the occurrence of necrotic cells, indicated by cellular uptake of amino cupric silver, the magnitude of the glial (microglia, astrocyte) response to treatment, the loss of staining for TH and an assessment of whether treatment triggered apoptotic mechanisms (caspase 3 expression and DNA fragmentation [TUNEL]).

### Statistical Analyses

The mean number of TH^+^ neurons in the SNpc of PQ- and MPTP-treated groups (left and right side combined) was compared statistically to the vehicle control group mean using either an analysis of variance (ANOVA) followed by a Student’s t-test [33], or, if variances were unequal, by a Welch’s t-test [34]. Since there was an *a priori* hypothesis that both PQ and MPTP would reduce the number of TH^+^ neurons in the SNpc, a one-sided t-test was performed.

The mean number of activated, resting and total microglia in the SNpc of vehicle controls was compared statistically to PQ and MPTP treated groups using a two-sided Welch’s t-test. A two-side test was used because it was considered equally likely that these agents could activate microglia as a result of DA neuron cell death or have a direct cytotoxic effect on glia (i.e. both a negative or positive response was possible). The relationship between the number of TH^+^ DA neurons in the SNpc and the number of microglia evaluated in the same animal within a group of controls, MPTP or PQ mice, was assessed by calculating the Pearson product-moment correlation coefficient. The statistical significance of the correlation coefficient was evaluated using a one-tailed t-test. The mean severity grade scores derived from the semi-quantitative neuropathological assessment of neurodegeneration and gliosis were evaluated using a one-sided Welch’s t-test (Z-scores reported). In all cases, a difference was concluded to be statistically significant when the probability of achieving the result by chance was less than 0.05. If the p value was lower, thereby reducing the chance of a type-1 error, this is stated.

## Results

### Body Weight, Food Consumption, Clinical Signs and Survival

PQ treatment (20 mg/kg/week for 3 weeks) resulted in a statistically significant reduction in body weight (S1 Fig) and food consumption (data not shown) after the first dose and less so after subsequent doses There were minimal clinical signs following PQ treatment and no effects on survival in either Jackson Laboratories or Harlan mice. MPTP-treated mice displayed hunched posture and hypoactivity after dosing and body weight was transiently reduced on the day of treatment. There were no effects of MPTP-treatment on survival in either the WIL or SJCRH studies.

### Concentration of Paraquat in the Brain

Following six ip doses of PQ (10 mg/kg/dose, twice weekly for 3 weeks), the mean concentration of PQ detected in brain was 0.54 ± 0.03 μg/gram tissue at 24 hours after the final dose. The amount of PQ remaining in the brain 24 hours after the final dose was less than 0.03% of the total dose injected (i.e. total PQ dose = 60 mg/kg, mean body weight ~25 g; mean brain weight ~ 0.45 g)

### Total number of TH^+^, Dopaminergic Neurons in the SNpc

C57BL/6J or C57BL/6NHsd mice, aged 9 or 16 weeks at initiation of dosing, were administered PQ ip twice weekly for 3 weeks (total of 6 doses at 10 mg/kg/dose) at both WIL and SJCRH, or once weekly for 3 weeks (total of 3 doses at 20 mg/kg/dose) at WIL (Table 1). The number of TH^+^ DA neurons in the SNpc was determined empirically 7 days after the final dose of PQ, using both design- or model-based stereology by two independent investigators who were blinded to experimental treatment. The number of TH^+^ DA neurons in the SNpc of PQ-treated groups was not significantly different from the respective control groups (Fig 1). In contrast, 7 days after MPTP treatment (20 mg/kg/dose x 4 doses), a statistically significant reduction in the number of TH^+^ neurons was observed in the SNpc in evaluations performed at both EPL and SJCRH (Fig 1).

The stereological results in both the PQ and MPTP-treated groups were consistent between laboratories and independent of: 1) the stereological method used to count TH^+^ DA neurons (design- vs. model-based stereology); 2) source of C57BL/6 male mice (Harlan or Jackson Laboratories); 3) age of mice at the time of dose initiation (9 or 16 weeks); 4) housing conditions (individually housed in wire-mesh cages at WIL vs. group housed in plastic shoebox-type cages at SJCRH); or 5) PQ dosing regimen (once weekly for three weeks at 20 mg/kg/dose vs. twice weekly for three weeks at 10 mg/kg/dose; S1 Table).

### Total number of resting and activated microglia in the SNpc

**Fig 2:**
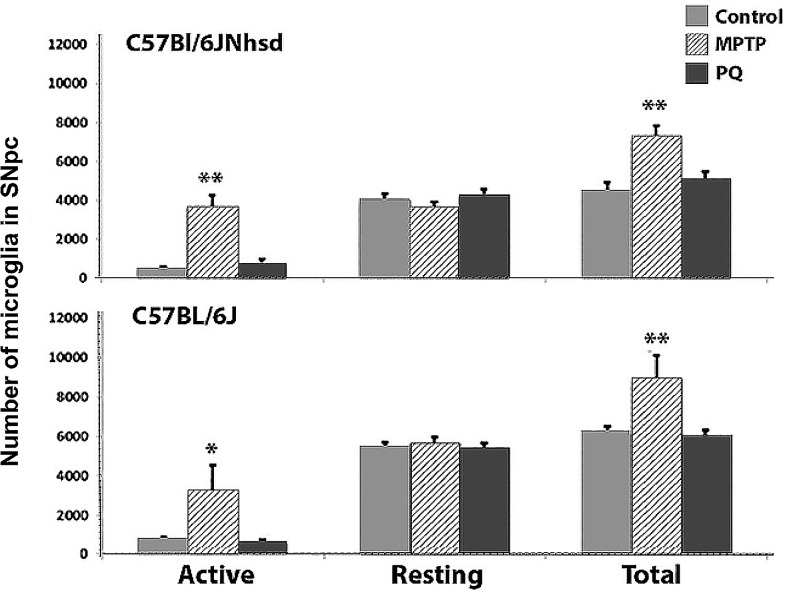
Stereological assessment of the mean number of resting and activated microglial cells in the SNpc of C57BL/6J or C57BL/6NHsd male mice treated with saline, PQ or MPTP. No change in resting or activated microglia number was seen in PQ-treated mice of either C57BL/6 substrain, while both C57BL/6 substrains demonstrated increased numbers of activated and total microglia 7 days following MPTP treatment. * p ≤ 0.05, **p ≤ 0.01.

**Fig 3:**
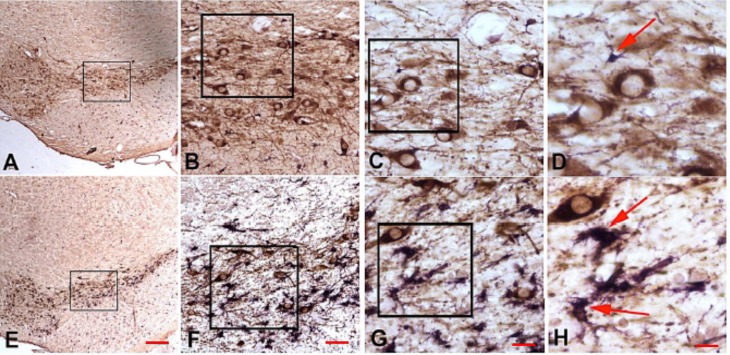
Appearance of microglia in PQ- and MPTP-treated mice. Representative photomicrographs of microglia (Iba-1+) cells in PQ-treated (Panels A-D) or MPTP-treated (Panel E-H) in the SNpc of C57BL/6J or C57BL/6NHsd male mice aged 9- or 16-weeks at the time of the 1^st^ dose. The boxes in each panel indicate the region shown in the adjacent box. Red arrow in D shows an example of resting microglia; characterized by a small cell body and thin processes. Red arrows in H show the typical appearance of activated microglia seen in MPTP-treated mice where the cell body is increased in size compared to resting microglia and the processes are shortened and thickened. Scale bars A,E = 100 μm, B,F = 40 μm, C,G = 20 μm, D-H = 10 μm.

C57BL/6J or C57BL/6NHsd mice, aged 9 or 16 weeks at initiation of dosing, were administered PQ ip twice weekly for 3 weeks at SJCRH. The number of resting and active microglia [31] were assessed using model-based stereology. PQ had no effect (Fig 2) on either the number of resting or active microglia in mice (Fig 3A–3D) from either source (C57BL/6NHsd [p≤0.08] or C57BL/6J [p≤0.44]). In contrast, 7 days after MPTP·HCl, there was a significant inflammatory response characterized by increased numbers of active microglia (Figs 2 and 3E–3H) in both C57BL/6NHsd (p≤0.0002) and C57BL/6J (p≤0.01) mice. The increase in active microglia resulted in a significant increase in the number of total microglia in both C57BL/6J (p≤0.008) and C57BL/6NHsd (p≤0.0008) mice (Fig 2).

### Correlation between activated microglia and TH^+^ neurons in the SNpc

**Fig 4:**
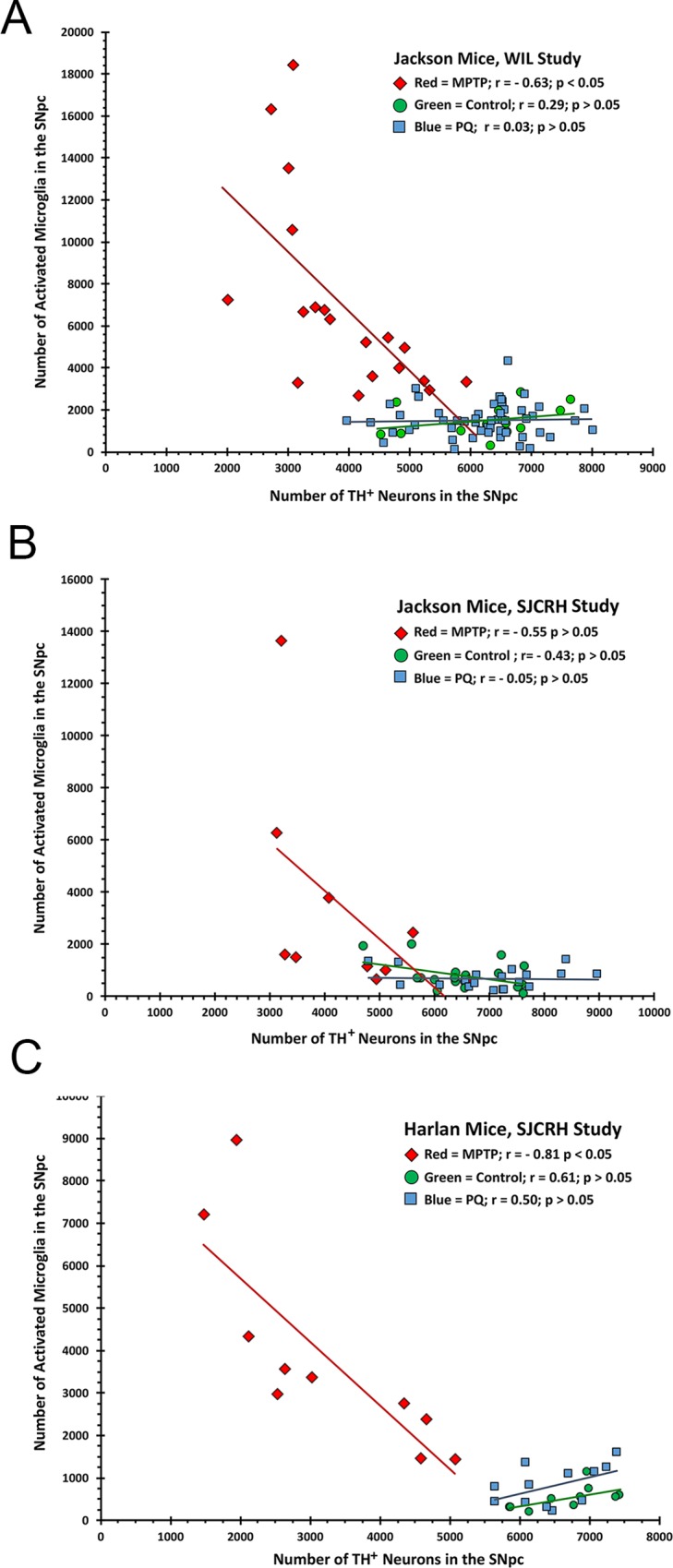
Correlation between the number of activated microglia in the SNpc and the number of TH^+^ neurons. The correlation between the number of activated microglia in the SNpc and the number of TH^+^ neurons was assessed in the same animal in two studies on C57BL/6J mice (A, B) and one study using C57BL/6NHsd (C) performed at two different sites (WIL, A) and SJCRH (B, C). A significant negative inverse correlation between TH^+^, DA neuron number and activated microglia was observed in MPTP-treated mice, while no correlation was seen in PQ- or saline treated mice.

In MPTP-treated mice, the number of activated microglia in the SNpc was inversely correlated with the number of TH^+^ neurons (Fig 4). The correlation coefficient was statistically significant for one study (WIL) using Jackson Laboratory-supplied mice (Fig 4A, r = - 0.63, p ≤ 0.05) but not in a second study (SJCRH) using mice from the same supplier (Fig 4B, r = - 0.55; p ≥ 0.05). The correlation coefficient was statistically significant in the study using Harlan mice (SJCRH, r = - 0.81, p ≤ 0.05). There was no statistically significant relationship between the number of microglia and the number of TH^+^ neurons in the SNpc of any control or PQ-treated group (Fig 4A–4C).

### Semi-quantitative neuropathological assessment of the effects of PQ and MPTP on the SNpc and Striatum

**Fig 5:**
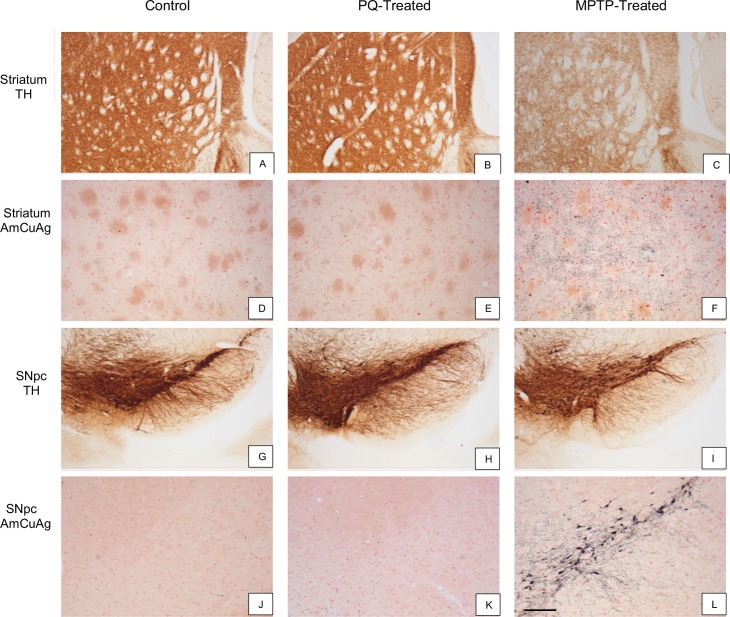
Pathological assessment of SNpc and striatum in PQ- or MPTP-treated mice. Microscopic appearance of the SNpc (G-L) and striatum (A-F) in control (column 1), PQ- (column 2) or MPTP-treated (column 3) mice, 48 hours after dosing. Tyrosine hydroxylase (TH) immunostaining was decreased in the striatum (C) and SNpc (I) of the MPTP-treated animal but was unchanged in the PQ-treated mouse (B, H) compared to the control (A, G). Amino cupric silver (AmCuAg) staining was used to reveal degenerating neurons in the SNpc (J-L) and degenerating fibers in the striatum (D-F). There were no differences in AmCuAg staining in either the SNpc or striatum after PQ treatment compared to the control (D,J). AmCuAg staining was increased in the SNpc and striatum of the MPTP-treated mouse (F,L). The scale bar shown in Panel L represents 400 μm in panels A-C, G-I and 40 μm in panels D-F and J-L.

**Fig 6:**
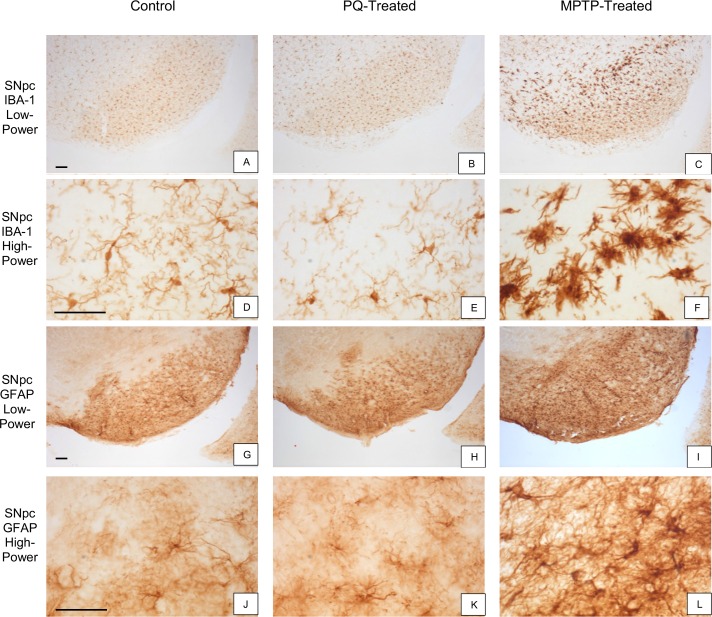
Appearance of microglia and astrocytes in SNpc and striatum of PQ- and MPTP-treated mice. Microscopic appearance of Iba-1-stained microglia (A-F) and GFAP-stained astroctyes (G-L) in the SNpc of mice 48 hours after PQ (column 2) or MPTP treatment (column 3) compared to a control (column 1). Iba-1 immunostaining of microglia was increased in the MPTP-treated mouse (C, F) but was not different in the PQ-treated mouse (B, E) compared to the control (A, D). Similarly, increased GFAP immunostaining of astrocytes was noted in the MPTP-treated mouse (I, L) but not in the PQ-treated mouse (H, K) compared to the control (G, J). Scale bar A-L 40 μm.

**Fig 7:**
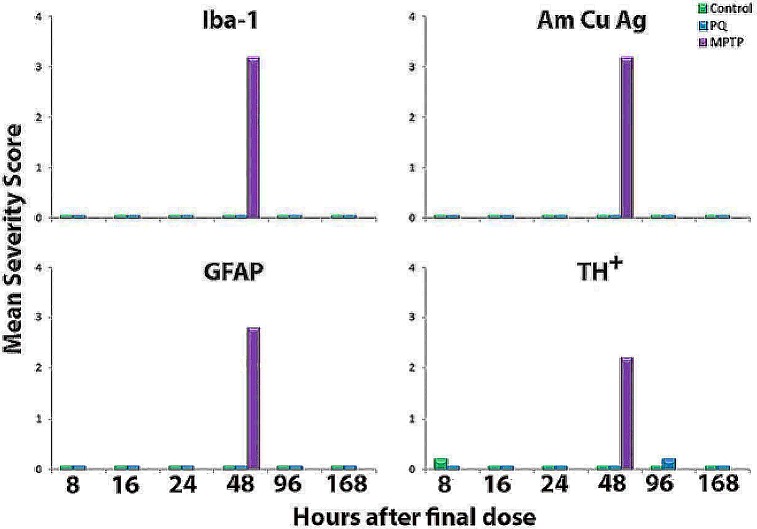
Mean histopathological severity scores in control, paraquat and MPTP-treated groups of C57BL/6J male mice. Mice were 16 weeks of age at the time of treatment initiation. Mice were administered 10 mg/kg/dose PQ·Cl_2_ by ip injection, twice a week for 3 weeks and were sacrificed 8, 16, 24, 48, 96 or 168 hours after the last dose. Control mice were given the vehicle while MPTP-treated mice received four injections of MPTP (16 mg/kg/dose; expressed as free base) at 2-hour intervals, and then euthanized 48 hours after the final dose. Serial sections through the SNpc were evaluated qualitatively and the group mean severity grades are plotted. Grades 0 to 5 reflect increasing intensity of staining for Iba-1, AmCuGg, GFAP and decreased staining intensity of TH.

C57BL/6J mice, aged 9- or 16-weeks at initiation of dosing, were administered PQ twice weekly for 3 weeks (6 doses at 10 mg/kg/dose) at WIL. Eight, 16, 24, 48, 96 and 168 hours after the final injection of PQ, the striatum and SNpc were examined for markers of TH expression (Fig 5A, 5B, 5G and 5H), axonal degeneration (Fig 5D, 5E, 5J and 5K), microgliosis (Fig 6A, 6B, 6D and 6E), and astrogliosis (Fig 6G, 6H, 6J and 6K). Semi-quantitative analysis demonstrated that PQ had no effect on TH^+^ immunostaining in either the striatum (Fig 5A and 5B and Fig 7) or SNpc (Fig 5G and 5H and Fig 7) at any of the time points evaluated. Additionally, there was no evidence of cell death (TUNEL, Caspase-3 staining; data not shown) or degeneration in either the SNpc or striatum (AmCuAg stain; Fig 5D, 5E, 5J and 5K and Fig 7) in either age group of C57BL/6J mice.

In contrast, by both qualitative observation (Figs 5C, 5F, 5I, 5L, 6C, 6F, 6I and 6L) and severity score (Fig 7, p ≤0.0001), TH^+^ immunostaining was significantly decreased in both the striatum (Fig 5C) and the SNpc (Fig 5I) at 48 hours post-MPTP administration. The severity score for degeneration (AmCuAg stain) was also statistically significant (Fig 7, p ≤ 0.0001) and qualitative markers of neurodegeneration were present in the striatum 48 hours after MPTP administration (Fig 5F and 5L). There was no evidence of apoptotic cell death in MPTP-treated mice (severity scores all equal to zero) (Individual animal data are presented in S2 Table).

The immunological response to PQ or MPTP, was evaluated in the striatum and SNpc by immunostaining for markers of microgliosis (Iba-1) and astrogliosis (GFAP). Semi-quantitative analysis demonstrated that PQ had no effect on microgliosis (Fig 6B and 6E and Fig 7) or astrogliosis (Fig 6H and 6K and Fig 7) in the SNpc or striatum (not shown) compared to saline-treated mice (Fig 6A, 6D, 6G and 6J and Fig 7). In contrast, 48 hours post-MPTP administration, the severity scores were increased for both microgliosis (Fig 6C and 6F and Fig 7 p ≤ 0.0001) and astrogliosis (Fig 6I and 6L and Fig 7, p ≤ 0.0001). Furthermore, activation of both microglia (Fig 6C and 6F) and astroglia (Fig 6I and 6L) was evident. (Individual animal data are presented in S2 Table).

## Discussion

Although a number of genes have been implicated in familial forms of Parkinson’s disease [35], the vast majority of cases likely arise from interactions between lifestyle risk factors, exposure to environmental agents [36–38] and genetic susceptibility factors [39–41]. It has been suggested that increased susceptibility for developing PD following exposure to environmental chemicals may result from genetic polymorphisms that affect metabolism, transport or modify the cell’s anti-oxidant defenses against free radicals [42–45].

Paraquat is capable of generating superoxide radicals that result in lipid peroxidation of cellular membranes [4, 5]. It has also been postulated that once inside cells, PQ can block the progression through the electron transport chain within mitochondria [46, 47] leading to an impairment of energy production and the generation of secondary oxidative stress and the initiation of cell death signaling [48]. There is also evidence that PQ can be transported into the brain via the neutral amino acid transporters [7] or through the organic cation transporter 2 and 3 (Oct3, Slc22a3) [8, 9]. However, as we report, only a small fraction of the administered dose (less than 1%) actually enters the brain.

It has been reported previously that PQ administered to C57BL/6J male mice causes a loss of DA neurons in the SNpc [19–21, 49] and induces microglial activation [50, 51]. However, in the current study, and as reported previously [22], PQ-treated mice did not display reduced numbers of TH^+^ neurons in the SNpc, and did not trigger any other indicator of neurotoxicity including loss of a dopaminergic phenotype, neuroinflammation, neuronal cell death or Wallerian degeneration. The same results were observed in two different substrains of the C57BL/6 mouse examined at two different ages (9 and 16 weeks at initiation of dosing). The veracity of these findings was confirmed in two independent laboratories, each using a different method of stereological assessment, as well as neuropathological assessment by a veterinary pathologist blinded to treatment. Additionally, these two laboratories, using different methods of stereology, reported essentially an equivalent magnitude loss of DA neurons in the SNpc of mice treated with the neurotoxin, MPTP.

The results of this study contrast with those from a number of studies that examined both SNpc TH^+^ neuron loss as well as neuroinflammatory effects in the basal ganglia following systemic exposure to PQ. Brooks et al [19] administered 10 mg/kg PQ once/week for three weeks (total of 30 mg), and reported a significant reduction in striatal DA terminals and a concomitant loss of SNpc DA neurons based on a non-stereological assessment of SNpc DA neurons. McCormack & Di Monte [52] used the optical fractionator approach to estimate SNpc DA neuron numbers in C57BL/6 mice administered 30 mg/kg PQ (10 mg/kg/week for 3 weeks) and reported a 25% loss of SNpc TH^+^ neurons, 7 days after the final injection of PQ. Jiao et al [21] reported a 50% loss of TH^+^ neurons in the SNpc, 7 days following administration of 60 mg/kg PQ (10 mg/kg/dose, twice weekly for 3 weeks).

A comprehensive review of 51 published studies (including this study) on the effects of PQ on SNpc DA neurons was conducted (S3 Appendix and S4 Appendix). This review showed that 28 of these published studies performed stereological assessments (73 group comparisons). In 47 of these 73 group comparisons, the investigators were unblinded with respect to treatment, and in 94% of these comparisons (i.e. 44 of 47), the mean number of TH^+^ neurons in the SNpc of PQ treated mice were reduced (the average reduction was 26.5% compared to the vehicle control group). Among the 26 (of 73 total) blinded comparisons, 81% (i.e. 21 of 26) were statistically null. Aside from blinding, the most noticeable difference between studies showing a PQ-induced cell loss and those that did not, was the observation that the average coefficient of variation (CV) among the “positive” studies (~ 6–7%) was statistically significantly less than the average CV observed in the negative studies (CV = 16.4%; p ≤ 0.0001). We examined factors that might contribute to the apparent greater precision in the statistically positive studies (S4 Appendix), but did not identify any single variable that was consistently different between positive and null studies on PQ. In comparison with much of the published literature, the current study used a relatively large number of replicates (n) per group and there was sufficient statistical power (0.994) to detect the average 26% reduction in the mean number of TH^+^ neurons reported in the statistically positive studies. No effect of PQ treatment was observed.

In addition to TH^+^ DA neuron number, we also stereologically estimated the total number of microglia (broken into resting and activated) in the SNpc using the same animals and sections in which TH^+^ neurons were counted. We found a significant inverse correlation between the number of TH^+^ neurons and activated microglia in MPTP-treated mice; i.e., mice that had low numbers of TH^+^ neurons in the SNpc tended to have increased numbers of activated microglia. No such relationship was noted in either the control or PQ-treated mice. These results are consistent with the absence of any effect of PQ on microglia in the semi-quantitative assessment of microglia in a second subset of mice that was evaluated by a neuropathologist who was blinded to treatment. These results are also consistent with the microglia findings of three other studies in which the investigators were blinded to treatment [22, 23, 53]. A review of quantitative inflammatory effects was not provided in 7 of 8 previously mentioned unblinded studies that reported more activated microglia activation in PQ treated mice (S3 Appendix). One blinded study [54] that used a statistical bootstrapping procedure found significantly more 7–8 μm diameter (activated) microglia and fewer 3 μm diameter microglia in PQ-treated mice compared to controls. Interestingly, in this study, PQ had no effect on the mean number of TH^+^ neurons in the SNpc. There were no effects of PQ on astrocytes in 4 out of 6 evaluations in the published literature (S4 Appendix).

It is unclear why the present investigation, in contrast to a number of other published investigations (see S4 Appendix), did not induce apparent dopaminergic cell loss in the SNpc or striatum following administration of PQ. One possibility is that, as compared to previous studies, the mice used in this study were of different ages, substrain, and/or were obtained from different vendors. To control for these differences, we examined C57BL/6 mice from two different vendors (Jackson Labs (C57BL/6J) and Harlan Laboratories (C57BL/6NHsd), and found no difference in their response to PQ. This suggests that substrain variability is not the explanation for the differential response observed in our studies vs. other studies. Additionally, previous studies examining PQ effects on SNpc DA neurons have been performed on mice of variable ages. In the current study we examined mice that spanned the ages of those previously reported and found no age-related differences in PQ sensitivity.

It is also possible that variability in processing and tissue handling could underlie differences in phenotypic expression of the TH immunostaining that was used to identify DA neurons. For example, in the Jiao study [21] where a 50% loss of SNpc DA neurons was observed, the sections of SN had been immunostained for TH several years after sectioning (Smeyne, personal communication); it is known that such delays may impair antigenicity in immunohistochemical preparations [55]. Additionally, tissues that are only post-fixed by immersion fixation rather than perfusion, as was the case for the brains evaluated in the McCormack et al. studies [20, 52], may also display a loss of sensitivity to the TH antigen.

Another potential explanation for our lack of any effect of PQ may be that insufficient amounts of PQ reached critical targets within the brain to induce degeneration. However, we administered near maximum tolerated, non-lethal doses to the mice in our study [22, 56] and found that 0.03% of the total amount of injected PQ (approximately 1.5 mg/mouse) was present in brain 24 hours after the last injection. This amount was similar to that predicted by pharmacokinetic modeling [22]. It is not possible to compare our results to those studies that reported cell loss because none of those papers examined brain PQ concentrations, but it is unlikely that equal or (often) lower doses of PQ administered in other studies would have resulted in higher concentrations of PQ in the brains of mice in these studies.

A third possibility is that PQ-induced neurodegeneration is seen only after a previous inflammatory insult that preconditions the CNS to the effects of PQ. For example, Brooks et al. [19] administered fluorogold as a tracer prior to administration of PQ. A number of studies have suggested that this tracer may, in and of itself, be toxic and induce neurological syndromes [57, 58]. Thus, it is possible that the tracer primed the CNS for PQ-induced damage, or even alone was responsible for the observed neuroinflammatory changes. Similarly, in the previous study conducted in our laboratory at SJCRH, [21], the C57BL/6J mice were initially housed in a standard (low) containment facility, which screens for, but allows exposure to, a number of pathogenic organisms, including *Klebsiella pneumonia* and nonpathogenic protists (e.g. trichomonads). Infection by such agents has been shown to induce a number of pro-inflammatory cytokines and activate microglia [59] and is linked to abnormal protein aggregation in the brain [60]. It has also been reported that previous neural inflammation and/or deposition of proteins [50, 61] can exacerbate the effects of oxidative stress inducing agents, potentially making non-pathogenic agents, such as PQ, pathogenic [62].

There remains significant controversy in the scientific literature regarding the neurotoxicity of paraquat as it relates to dopaminergic neurotoxicity [19–21, 23, 49, 63–65]. We have not resolved the controversy with this study. However, using three methods of assessment performed by three groups blinded to treatment for a series of neuropathological indices evaluated in two ages and two sub-strains of male C57BL/6 mice housed under different conditions in two laboratories, PQ administered at maximum tolerated doses did not induce any neuropathogenic effects.

## Supporting Information

S1 Appendix HPLC-MS/MS method used to quantify the concentration of PQ in the brain of mice. (DOCX)

S2 Appendix Neuropathological Grading System. (DOCX)

S3 Appendix A systematic review of the published literature that has evaluated the effects of paraquat on the SNpc and striatum in male mice. (DOCX)

S4 Appendix Stereological and neuropathological studies on paraquat in mice. (DOCX)

S1 Fig Mean body weight over the course of the study. Mice were either 9 weeks of age (9Wk, solid lines) or 16 weeks of age (16Wk, dashed lines) at the time of treatment initiation on Study Day 1. Mice receiving 10 mg/kg/dose PQ·Cl_2_ were injected with the test item formulation on Study Days 0, 3, 7, 10, 14 and 17, and then euthanized at 8, 16, 24, 48, 96 and 168 hours after the final dose (neuropathology) or on Study Day 24 (stereology). Mice receiving 20 mg/kg/dose PQ·Cl_2_ were injected with the test item formulation on Study Days 0, 7 and 14, and then euthanized at 8, 16, 24, 48 96 and 168 hours after the final dose (neuropathology) or on Study Day 21 (stereology). MPTP mice received four injections of MPTP (16 mg/kg/dose; expressed as free base) at 2-hour intervals on Study Day 17, and then euthanized on either Study Day 19 (48 hours after the final dose; neuropathology) or Study Day 24 (stereology). Control mice were administered saline vehicle ip on the same days as the 10 mg/kg/dose PQ·Cl_2_ mice. Body weights were measured and recorded daily.
(TIF)

S1 Table Statistical comparisons of the effect of animal supply, age, housing, paraquat dose frequency or stereological method on the number of TH^+^ neurons in control, paraquat or MPTP treated groups of mice. (DOCX)

S2 Table Individual Animal Neuropathology Severity Grade in the SNpc of 9 Week Old C57BL/6J Mice (WIL Study): 8 Hours Post-Dosing. (DOCX)
