# Supplementary material for: Assessment of the Effects of MPTP and Paraquat on Dopaminergic Neurons and Microglia in the Substantia Nigra Pars Compacta of C57BL/6 Mice
Source: PLoS One. 2016 Oct 27;11(10):e0164094. doi: 10.1371/journal.pone.0164094 (PMC5082881; doi:10.1371/journal.pone.0164094)
Supplement: S1 Appendix — (DOCX) [file pone.0164094.s001.docx]

**S1Appendix: HPLC-MS/MS method used to quantify the concentration of PQ in the brain of mice**

**Testing Facility:** Charles River Laboratories, Chemistry Department, Tranent, Edinburgh, EH33 2NE, UK

**Study Number:** 223875

**Analytical Method:** Charles River Analytical Method No. 8936 Version 2

**Purpose of Method:** To quantify the concentration of paraquat in brain samples from mice that had been administered paraquat dichloride by i.p. injection.

**Analytic Standard:**

**Paraquat dichloride:** Batch SZBB348XV

**Physical Description:** Crystalline solid

**Molecular Formula:** C_12_H_14_Cl_2_N_2;_

**Molecular Weight**: 257.16 g/mol

**Molecular Weight as cation:** 186.3 g/mol

| **Chemical Structure:** | 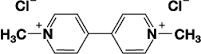 |
| --- | --- |

**Source:** Sigma Aldrich Company, Ltd., (Gillingham, Dorset)

**Purity:** 99.9%;

**Expiration Date:** December 14, 2016.

**Storage:** Room temperature

**Study Initiation Date:** August 12, 2014

**Sponsor**: Syngenta Ltd., Jealott’s Hill Research Centre, Bracknell, Berkshire, RG42 6EY

**Brain Samples:** Whole mouse brains, excluding olfactory bulbs, were collected 24 hours after the last PQ dose from a group of 5 mice. Each mouse received a total of six doses of PQ administered by i.p. injection (10 mg/kg/dose) over a 3 week period. The samples were shipped on dry ice from SJCRH to Charles River Laboratories, UK.

**Sample Storage:** Brain samples were stored frozen at - 20^O^C until analyzed.

**Homogenization of Brain:** The whole brains (excluding olfactory bulbs) were homogenized and 50 mg samples of the homogenate were analyzed.

**Extraction Reagents:**

**Acetonitrile:** Rathburn Chemical (HPLC grade).

**Formic acid:** Fisher Chemical (Analytical grade)

**Distilled water:** Charles River

**Trichloroacetic acid:** Fisher Chemical (Analytical grade)

**Ammonium formate:** Sigma Aldrich (Analytical grade

**Paraquat Extraction:**

1. 50 mg of mouse brain homogenate was added to a 2 mL Eppendorf tube and 200 µl of 10% trichloroacetic acid solution.
2. Suspension homogenized for ~ 15 seconds with a fine-tipped homogenizer.
3. Centrifuged (13000 rpm) for 5 minutes.
4. 75µl supernatant transferred to an LC vial
5. 250 µl of 0.25M ammonium formate, pH 3.7 added (mobile phase A)
6. 150 µl of acetonitrile added (mobile phase B)

**Chromatographic Analysis**

**High Pressure Liquid Chromatograph (HPLC) Equipment**

**HPLC System (Pump, Autosampler, Column, Oven):** Perkin Elmer Series 220

**Analytical Column:** Atlantis HILIC Silica 3 µm, 100 x 3 mm

**Detector:** Applied Biosystems SCIEX API 4000

**Data System:** Analyst Version 1.1

**HPLC Settings**

**Mobile Phase A:** 0.25M ammonium formate, pH 3.7

**Mobile Phase B**: Acetonitrile

**Injection Volume:** 30 µl

**Flow Rate:** 1.0 mL/min

**Oven Setting:** ~ 4 ^O^C

**Autosampler Temperature:** ~ 4 ^O^C

**Column Temperature:** ~ 40 ^O^C

**Retention Time:** ~ 3 minutes

| **Gradient:** | **Time (min)** | **(%) A** | **(%) B** |
| --- | --- | --- | --- |
|  | 0.0 | 60 | 40 |
|  | 7.0 | 60 | 40 |

**Mass Spectroscopy Conditions**

| **Scan Type:** MRM | **GS2:** 60 |
| --- | --- |
| **Polarity:** Positive | **Ion Spray Voltage:** 1500V |
| **Ion Source:** Turbo ion spray | **Temperature:** 600 ^O^C |
| **Transitions:** 93.1 – 171.2 m/z | **Declustering Potential:** 40V |
| **CAD Gas:** 5 | **Entrance Potential:** 12V |
| **Curtain Gas:** 15 | **Cell Exit Potential:** 5V |
| **GS1:** 40 | **Collision Energy:** 20eV |

**Analytic System Calibration**

**Analytic Standard:**

1. Corrected the analytic standard for purity and salt content (Equation 1)
2. Oven dried paraquat analytic standard at 100 ^O^C and store in dessicator
3. Prepared paraquat stock solution (200 µg/ml)
4. Diluted the stock solution with water to achieve the following final concentration standards: 0.2, 0.1, 0.05, 0.02, 0.01, 0.005, 0.002, 0.001 and 0.0005 µg/ml

Actual Mass = Molecular Weight _Cation_ x Purity (%) (Equation 1)

Molecular Weight _Salt_  100

**Linearity of Analytic System**

The linearity of the analytic system was confirmed prior to analysis of mouse brain samples (See Supplemental Figure 1.1 and 1.2)

Figure 1.1 Extracted Calibration Line for a Control Mouse Brain Fortified with Paraquat (0.5 - 20 ng/g)

Figure 1.2 Representative Chromatogram of an Extracted Calibration Standard at the Lower Limit of Linearity (0.5 ng/g)

**Lower Limit of Quantitation (LLC)**

The lower limit of quantification of paraquat in mouse brain was 0.5 ng/g brain sample.

**Procedural Recovery**

The recovery of paraquat from brain samples that had been fortified with paraquat at concentrations of 0.5, 1, 5 and 10 ng/g were 110-126, 108-115; 88.6-103 and 85.7-98.1% respectively. Due to response drift after the calibration line was injected, the sample batch was processed from bracketing standards interspersed at regular intervals throughout the run.
