# Supplementary material for: Assessment of the Effects of MPTP and Paraquat on Dopaminergic Neurons and Microglia in the Substantia Nigra Pars Compacta of C57BL/6 Mice
Source: PLoS One. 2016 Oct 27;11(10):e0164094. doi: 10.1371/journal.pone.0164094 (PMC5082881; doi:10.1371/journal.pone.0164094)
Supplement: S2 Appendix — (DOCX) [file pone.0164094.s002.docx]

**S2 Appendix: Neuropathological Grading System^1^**

Semi-quantitative grades were assigned to each section based upon the percentage of the slide evaluated displaying the finding. The directionality of the effect (i.e. decrease or increase)

was based on the effect expected for the MPTP, positive control animals. All evaluations were performed by a board certified pathologist (MB) who was blinded to treatment, which is typical for a morphologic assessment conducted for a GLP compliant study.

| **Diagnosis/Stain/Site** | **Grade 0 normal** | **Grade 1 slight** | **Grade 2 Minimal** | **Grade 3 Mild** | **Grade 4 Moderate** | **Grade 5 Severe** |
| --- | --- | --- | --- | --- | --- | --- |
| Necrosis/AmCuAg SNpc | No necrotic neurons | <1% | 1 to 5% | 5 to 15 % | 15 to 40% | >40% |
| Disintegrating synaptic terminals/AmCuAg striatum | No staining of synaptic terminals | <1% Disintegrating synaptic terminals | 1 to 5% Disintegrating synaptic terminals | 5 to 15 % Disintegrating synaptic terminals | 15 to 40% Disintegrating synaptic terminals | >40% Disintegrating synaptic terminals |
| Decrease TH+ neurons/TH+ stain/SNpc | Densely stained neurons | 1% to 20% decrease in staining | 20% to 40% decrease in staining | 40% to 60% decrease in staining | 60% to 80% decrease in staining | 80% to 100% decrease in staining |
| Reactive Astrocytes/GFAP/ SNpc or striatum | No reactive astrocytes | 1% to 20% reactive astrocytes | 20% to 40% reactive astrocytes | 40% to 60% reactive astrocytes | 60% to 80% reactive astrocytes | 80% to 100% reactive astrocytes |
| Reactive microgliosis/Iba-1 /SNpc or striatum | No reactive microgliosis | 1% to 20% reactive microglia | 20% to 40% reactive microglia | 40% to 60% reactive microglia | 60% to 80% reactive microglia | 80% to100% reactive microglia |

^1^Since there was no evidence of apoptosis (Caspase 3 immunostaining) or Terminal deoxynucleotidyl transferase dUTP nick end labeling (TUNEL) in any animal in any group, no severity grade was calculated for these endpoints.

AmCuAg – Tissue sections histochemically stained for Amino Cupric Silver

TH^+^ – Neurons immunolabeled for Tyrosine Hydroxylase

GFAP – Astrocytes immunolabeled for Glial Fibrillary Acidic Protein

Iba-1 – Microglia immunolableled for Ionized calcium-binding adapter molecule 1
