# Supplementary material for: Assessment of the Effects of MPTP and Paraquat on Dopaminergic Neurons and Microglia in the Substantia Nigra Pars Compacta of C57BL/6 Mice
Source: PLoS One. 2016 Oct 27;11(10):e0164094. doi: 10.1371/journal.pone.0164094 (PMC5082881; doi:10.1371/journal.pone.0164094)
Supplement: S3 Appendix — (DOCX) [file pone.0164094.s003.docx]

**S3 Appendix: A systematic review of the published literature that has evaluated the effects of paraquat on the SNpc and striatum in male mice.**

**INTRODUCTION**

Studies commissioned by Syngenta and conducted in independent laboratories [1, 2] have shown that paraquat (PQ), administered to C57BL/6J mice by intraperitoneal (i.p.) injection or in the diet at maximum tolerated doses, had no effect on the estimated mean number of TH^+^ neurons in the SNpc or TH^+^ axons and terminals in the striatum. There was no evidence of neuronal cell death in the SNpc, degenerating axons projecting to the striatum or gliosis in the SNpc or striatum. Dopamine (DA) levels and DA turnover in the striatum of PQ-treated mice were comparable to controls. However, there are many published studies that have reported effects of PQ on these parameters. The purpose of this evaluation was to conduct a systematic review of the published literature on the *in vivo* effects of PQ on dopaminergic systems in mice. Published data for the rat or other species, and the results from behavioral investigations, were not included because these species and endpoints were not evaluated in studies conducted as part of the current investigation. In addition to the animal (strain, source, age and husbandry) and dose variables (source of PQ, dose route, dose level and frequency and the duration of treatment), the potential impact of stereological, methodological and other factors on experimental outcomes, were evaluated.

**METHODS**

**Literature search**

A comprehensive search of the English language literature was conducted in order to identify all published studies on PQ (paraquat, gramoxone or methyl viologen). Libraries searched included PubMed, CAB Abstracts (Ovid), BIOSIS (Ovid), Embase (OVID), CAPLUS (SciFinder), CiNii, and WorldCat. In addition, all references contained in a Syngenta publication database on PQ dating back to 1960 were included. All citations were entered into a PQ-specific EndNote^®^ database (EndNote^®^ Version X4). EndNote^®^ search tools were used to identify all studies conducted on PQ that use the mouse as the experimental model. Abstracts, letters to editors and summaries of presentations or the proceedings of scientific meetings were excluded. Every publication identified in this search was evaluated to determine whether stereological, neuropathological or neurochemical data had been collected and reported for male mice. If such data were reported, then the information for the following data fields was extracted into tables:

Animal: Strain, source, age, number/group, sacrifice method.

Husbandry: Housing, quarantine duration.

Dosing: Paraquat source, route, dose, frequency/duration, analytic method and whether positive or vehicle control groups were included.

Histology: Tissue collection, microtomy and staining methodology.

Stereology: Type, section thickness, number of sections, disector height, guard zone.

Neuropathology: Methods used for quantification.

Neurochemistry: Effects of PQ on DA levels and DA turnover.

Blinding: Whether the investigators were blinded to treatment.

**Data extraction**

Only data from wild-type mice were summarized from studies in which both wild-type and genetically-modified mice were included. Results were excluded from studies in which mice were exposed to PQ and a second chemical or to some other experimental intervention that was predicted to augment or attenuate the effect of PQ. For any study in which a quantitation of the effect of PQ on TH^+^ neurons was provided, either in tables or figures, the mean, the standard error of the mean (SEM) and the numbers of mice in the control and PQ-treated group were extracted. Data presented in figures were extracted by copying the appropriate figure into a scaled digitized graph in order to estimate the mean and SEM. These estimates were validated against results obtained from a computer program (Plot Digitizer 2.6.8; October 27, 2015) that calculated the mean and SEM from digitized images.The absolute number of TH^+^ neurons or Nissl-positive neurons was determined for the control and PQ-treated groups. When the investigator reported the effect of PQ on the number of TH^+^ neurons as a percent of controls, those values were also captured.

**Data tabulation**

Qualitative and semi-quantitative data from publications that reported stereological and/or neuropathological findings were tabulated (S4 Appendix). Key study-related data were extracted from 50 publications that reported stereological and/or neuropathological data (including the present study). The effect of PQ on DA and/or DA turnover was extracted from 26 publications that reported neurochemistry data. For the purposes of the categorical analyses described below, the present study was counted as two independent studies because separate groups of mice were housed at different times in two different facilities (i.e. WIL Research and the vivarium of St. Jude Children’s Research Hospital).

Categorical data that described whether PQ had an effect on each experimental parameter evaluated were extracted and tabulated. If statistical analysis was conducted by the investigators, any study that reported a statistically significant difference between the PQ-treated group and the control group was recorded as indicating there was a statistically significant effect. If the investigator concluded that PQ had an effect on a parameter, based on qualitative data (e.g. subjectively evaluated change in intensity or frequency of staining for neuropathological endpoints like microglial activation), then these results were also recorded as an effect of treatment.

Quantitative data on the effect of PQ on the number TH^+^ neurons in the SNpc, or on the intensity of TH staining in the striatum, was represented as percentages of control values. In those studies that reported the estimated mean (±SEM) number of TH^+^ neurons or the number of Nissl-stained neurons in the SNpc of control and PQ-treated mice, such values were tabulated and the statistical significance of any differences was recorded. In a number of studies, the same control group was used for several statistical comparisons within the study, and in some cases it appeared that the same data were presented in multiple publications (e.g. Peng et al. [3-6]). Nevertheless, for the purposes of this assessment, all the data were retained and no correction was made for the lack of statistical independence arising from the use of a common control group(s) within a study.

**Statistical analyses**

For studies where an estimate of the absolute number of TH^+^ or Nissl-positive neurons was provided for the control and PQ-treated groups, a coefficient of variation (CV, as a percentage) was calculated (Equation 1) based on the mean and SEM. The standard deviation (SD) was calculated based upon the SEM and the number of mice reported for the group. In many instances, investigators reported the number of mice in a group as a range, or generally described the number of mice used in the method section. In those cases the mid-point or the upper end of the reported range was used.

***CV=* (SD ÷ Mean) x 100% (Equation 1)**

There were 73 stereological group comparisons in 28 studies that provided estimates of the mean (±SEM) number of TH^+^ neurons in the SNpc of control and PQ-treated mice. A group of 49 stereological results, reported as statistically significant (i.e. positive studies), were compared to a group of 24 negative studies reported as statistically null. Differences in means, standard deviations (SDs), and coefficients of variation (CVs), and in the distributions of the results for statistically positive studies were compared to results from null studies using one-sided Welch’s t-test (means), two-sided F-test (variances), and two-sided Kolmogorov-Smirnov test (distributions). Differences were considered statistically significant if the probability of observing each difference was less than 5%.

**Sensitivity analyses**

Monte Carlo simulations were performed using Microsoft Excel (Version 14) and the data reported by Baquet et al. [7] to evaluate the impact on the estimated number of TH^+^ neurons in the SNpc when 1 in every 3^rd^, 6^th^, 9^th^ or 12^th^ section was sampled. The total number of TH^+^ neurons in the SNpc was estimated using TH^+^ neuronal counts reported for the left, right or both sides of the SNpc. For these analyses, piecewise linear interpolation was used to estimate the number of TH^+^ neurons in 10 μm bins based on the values reported by Baquet et al. [7] for 50 μm bins in the SNpc in four serially reconstructed brains of 4 control mice. The use of interpolation did not alter the total number of TH^+^ neurons reported by Baquet et al. [7].

Sensitivity analyses were also conducted using data reported by Breckenridge et al. [1], in order to evaluate the impact on the estimated mean number of TH^+^ neurons of the SNpc of the following variables:

1. the interval between sections evaluated (1 every 3^rd^, 6^th^ or 12^th^ section)
2. whether TH^+^ neurons were counted in the left, right or both sides of the SNpc
3. the disector height and/or guard zone height and
4. the combined variability in the stereologist’s judgment in drawing contours around the SNpc and/or in identifying and counting TH^+^ neurons and the variability resulting from the random placement of counting frames by the stereology software.

**RESULTS**

**Categorical stereological data from published studies**

Of the 47 studies that evaluated the effect of PQ on the number of TH^+^ neurons in the SNpc, 38 of the studies (81%) reported a statistically significant reduction in the number of TH^+^ neurons in the SNpc of male mice administered high doses of PQ (1 – 20 mg/kg), usually once or twice weekly by i.p. injection, typically over 3 weeks (Table 3.1). Eight of the 38 positive studies (21%) were conducted with the investigator blinded to treatment whereas 6 of the 9 null studies (67%) were conducted blinded.

A review of the meta-data from the statistically null studies compared to the statistically positive studies (Table 3.1 and 3.2; S4 Appendix) indicated that the age of the mice at commencement of dosing, the frequency, duration and dose level used, were not predictive of whether statistically positive or null results were obtained (Table 3.2). There was insufficient information in the published record to determine whether animal husbandry practices had an influence on outcome but this was considered unlikely because the majority of the studies were conducted in modern facilities whose animal practices were governed by similar regulations.

**Quantitative stereological data from published studies**

Among the 47 studies that evaluated the effect on PQ on TH^+^ neurons in the SNpc, the estimated number of TH^+^ neurons was reported in 28 studies (Table 3.2). In 73 comparisons from these 28 studies, the mean number of TH^+^ neurons in PQ-treated mice was compared to a control group mean. The estimated mean number of TH^+^ neurons in PQ-treated groups was reported by the authors as statistically significantly less than the mean number of TH^+^ neurons in the control group in 67% (49 of 73) of the group comparisons (Table 3.2). Ninety-four percent of the non-blinded group comparisons (44 of 47) were statistically significant, whereas only 19% of the blinded assessments (5 of 26) were significant and 81% (21 of 26) of the comparisons were not.

Among the statistically significant positive studies, the mean number of TH^+^ neurons in the SNpc was, on average, 26.5% less in the PQ-treated groups compared to controls, whereas in the statistically null studies, on average there were 5.2% fewer TH^+^ neurons in PQ-treated mice (Figure 3.1). The average CV for the vehicle control and PQ-treated groups among the statistically positive studies were 5.6 and 7.2%, respectively. These CVs were statistically significantly different from the CVs for the statistically null studies (i.e. control and PQ-treated group CVs were 16.4% in the null studies). Among the unblinded positive studies, the CV for the control and PQ-treated groups were 5% and 7%, respectively and the corresponding CV for the blinded null studies were both 18% (Figure 3.1). In the present investigation, the average statistical power to detect a 26.5% reduction in TH^+^ neurons in the SNpc, was greater than 0.994.

**Sensitivity analyses: Variability in estimates of the mean number of TH^+^ neurons**

**Inter-animal variability**

In the study by Baquet et al. [7], all TH^+^ neurons were counted in successive 10 µm sections taken from the rostral to caudal extent of the SNpc of 4 male C57BL/6J mice. The mean (± SEM) number of TH^+^ neurons in this serial reconstruction of the SNpc was 8305 (± 540; CV = 13%; Figure 3.2). A design based (40 µm nominal thick section; disector height = 20 µm; guard zone = 1 µm top and bottom, counting frame 60 x 60 µm; 1 in 6 sections evaluated; N= 10 mice) 3D stereological evaluation yielded an estimate of the total number of TH^+^ neurons (8716±338) that was within 5% of the serial reconstruction; the CV (7.8%) was 40% less than the CV calculated based on serial reconstruction. In the model based 2D assessment (nominal thickness = 10 µm; 1 in every 3 sections evaluated; N=10), the estimated mean number of TH^+^ neurons in the SNpc (8002±91) was within 8% of the serial reconstruction, but the CV was approximately 83% less than the CV calculated based on serial reconstruction.

**Inter-hemispheric variability**

The mean number of TH^+^ neurons in the SNpc in the left hemisphere of the brain was comparable to the estimated mean number in the right hemisphere for a group of control mice. However, individual mice displayed up to a 3-fold difference between hemispheres (Figure 3.3; Table 3.3). Inter-hemispheric variability in the number of TH^+^ neurons would contribute to between-animal variability in the estimate of the mean number of TH^+^ neurons only if the investigator sampled one side of the brain. This information was not provided in the majority of studies (S4 Appendix).

**Medial-lateral and dorsal-ventral variability within the SNpc**

Fernagut et al. [8] estimated that out of a total of 10,416 TH^+^ neurons in the SNpc there were 33.3%, 11.2%, 31.5% and 23.9% in the medial (3472), lateral (1167), dorsal (3285) and ventral (2492) SNpc, respectively . Watson et al. [9] obtained similar results with 30.7%, 7.7%, 43.8% and 17.8% in the medial, lateral, dorsal and ventral SNpc. The 3-fold difference in the number of TH^+^ neurons between sub-regions of the SNpc is not expected to affect the estimate of the total number of TH^+^ neurons in SNpc as long as the stereological software randomly superimposes counting frames with approximately equal frequency throughout the entire contoured SNpc section.

**Inter-laboratory variability**

The mean number of TH^+^ neurons in the SNpc of control C57BL/6 mice that were 6 to 13 weeks of age) was highly variable between studies (Mean = 11133; SD = 2610; SEM = 533; Range =4444 to 15173). These inter-laboratory differences (up to 3.4-fold; Figure 3.4), were not due to differences in the age of the mice (Figure 3.4) but more likely reflect differences in factors related to the stereological assessment methodology (e.g. disector height and placement) and/or the criteria used by the stereologist to define the location of the SNpc or to identify TH^+^ neurons within the SNpc.

**Intra-reader variability**

Intra-reader variability was calculated based on differences in the mean number of TH^+^ neurons recorded for two C57BL/6J male control mice that were evaluated on multiple occasions within a study [2]. The stereologist was blinded to treatment, so when presented with the brain sections from the same animal on multiple occasions based on a block random design, the stereologist did not know to which group the animal belonged, nor the fact that the brain had been evaluated previously. Therefore, on each assessment occasion, new contours were drawn around sections of the SNpc and new, randomly assigned counting frames were assessed. The results (Table 3.4) indicate that the intra-reader CV ranged from 8-9%.

**Variability due to stereological factors**

**Effect of guard zone and disector height**

In the majority of stereological studies conducted on paraquat (S4 Appendix), including our own studies [1, 2], a guard zone of 2 µm and a disector height of 4 µm were used. In the present investigation, and in a reevaluation of the control group from a previously reported study [1], TH^+^ neurons were counted throughout the entire depth of section (nominal section thickness = 40 μm; measured thickness after tissue processing = 14-18 μm). The results from Breckenridge et al. [1] indicate that the mean number of TH^+^ neurons was not uniform throughout the depth of the section (Figure 3.5, top) but instead displayed a bimodal density of TH^+^ neurons that peaked within approximately 6 μm of the top or bottom of the section. The CV for the number of TH^+^ neurons counted within 2-6 um of the surface of the section was smallest in this region and the CV increased progressively toward the middle portion of the section (Figure 3.5, bottom).

The mean number of TH^+^ neurons in the SNpc was overestimated by ~37% when a 4 μm disector height was used compared to the estimate that was based on counting TH^+^ neurons throughout the entire depth of section (i.e., disector height = 2 to 15 µm; Figure 3.6). This difference was attributed to the unequal density of TH^+^ neurons throughout the depth of sections Therefore, when the disector height was set equal to the entire depth of field, excluding the 1 um guard zone, the estimated number of TH^+^ neurons closely approximated the estimate reported by Baquet et al. [7] based on serial reconstruction, 2D stereology, or 3D stereology using a disector height of 20 μm and a guard zone of 1 μm.

**Effect of the number of sections evaluated**

The impact of varying the number of sections evaluated on the estimated mean number of TH^+^ neurons was assessed by stereologically sampling 1 in every 3, 6, 9 or 12 sections in control animals from Baquet et al. [7] and in 1 in every 3, 6 or 12 sections from the control animals displayed in Figure 3.5. The estimated mean number of TH^+^ neurons, based on infrequent sampling of sections through the SNpc (i.e. 1 in every 12 sections), was not significantly different from results obtained when two or four times as many sections were evaluated (Table 3.5). Similar insensitivity was found when the mean data reported by Baquet et al. [7]; Figure 3.2 was sampled at different frequencies through the rostral-caudal extent of the SNpc (data not shown).

**Estimated mean number of TH^+^ vs. Nissl-positive neurons in the SNpc**

It is recognized that identification and enumeration of DA neurons in the SNpc based on stereological methods depend upon both the phenotypic expression of TH in DA neurons and the ability of specific antibodies to bind to epitopes on TH. Therefore, investigators have used alternate histochemical methods to identify losses in DA neurons. Although not specific for DA neurons, Nissl stains (e.g. cresyl violet) have been the method of choice in studies on PQ. In a subset of these studies, quantitative estimates of the “mean number of DA neurons lost in PQ treated mice” was estimated using TH^+^ and Nissl-positive stains. The results indicate that there is reasonable concordance between the reduction in TH^+^ and Nissl-positive neurons for the statistically significant positive studies, but less of a concordance in the statistically null studies (Table 3.6; Figure 3.7).

**Neuropathology**

There were 7 studies in the published literature that investigated the effects of PQ on neuropathological indicators of cell death (AmCuAg, Fluoro-Jade B, Caspase 3, TUNEL) in the SNpc and/or striatum (Table 3.1). In the 4 positive studies, the investigators did not provide quantitative data, but only showed “representative photomicrographs indicating that PQ increased Fluoro-Jade, silver or TUNEL staining” [10-12]. In contrast, in the series of 3 studies we conducted [1, 2], the neuropathologist, using a categorical severity scale, reliably identified all MPTP-treated mice (~95 mice) but could not distinguish PQ-treated ~545 mice from ~120 control mice, based upon multiple indicators of cell death (AmCuAg, Caspase 3, TUNEL).

There were 13 studies in the published literature that evaluated the effects of PQ on microglia in the SNpc and/or striatum and 5 studies on astrocytes. No effects of PQ were observed in 5 out of 13 studies on microglia or in 4 out of 5 studies on astrocytes (Table 3.1, S4 Appendix). The positive studies, except for Watson et al. [9], did not provide quantitative data. Watson et al. [9], reported an increase in the diameter of Iba-1-stained microglia in PQ-treated mice in the absence of any effect of PQ on the number of TH^+^ neurons in the SNpc or on TH staining in the striatum.

In contrast, in 3 negative studies [1, 2], the neuropathologist reliably separated all of the MPTP-treated mice from control and PQ-treated mice based upon a blinded evaluation of Iba-1 (microglia) and GFAP (astrocytes). The microglia results were also confirmed in the present investigation by an independent stereological assessment of the number of active vs. resting microglia in the SNpc.

**Neurochemistry**

PQ had no effect on DA levels in 15 out of 26 published studies (Supplemental Table 3.7). DA levels were decreased in 8 studies and transiently decreased in 1 study. DA levels were transiently increased in 1 study and increased in a second study. Dopamine turnover was unaltered in 8 of 12 studies, decreased in 2 studies and increased or increased followed by a transition to no effect in 2 studies.

**DISCUSSION**

**Stereology**

The most noticeable difference between “PQ-positive” and “PQ null” studies was that the null studies were generally conducted with the investigator blinded to treatment, whereas in the positive studies the investigator was not blinded to treatment. Related to this was the finding that the mean CV’s in the control (CV= 5.6%); and the PQ-treated group (CV =7.2%) for “positive” studies were statistically significantly less (p < 0.0001) than the mean CV’s for the negative studies (CV_Control_ = 16.4%; CV_PQ_ =16.4%). In spite of larger CVs in blinded studies compared to unblinded studies, the present investigation had greater than a 99.4% probability of detecting the 26.5% mean reduction in TH^+^ neurons reported in statistically positive studies.

Several factors that might contribute to the variability in stereological estimates of the absolute number of TH^+^ neurons in the SNpc of C57BL/6 mice were considered. The CV attributed to between-animal variability was 13% based on the Baquet et al. [7] reconstruction of the SNpc of male mice. In individual animals, up to a 3-fold difference in the mean number of TH^+^ neurons was observed in the left vs. the right hemispheres (Figure 3.3; Breckenridge et al. [1]). Up to a 3-fold difference was also noted in the mean number of TH^+^ neurons in medial vs. lateral aspects of the SNpc [8, 9]. Variability of 8-9% was attributed to the stereologist and/or the random placement of counting frames on the sections selected to be counted (Table 3.5; [2]) and between-laboratory differences in the estimate of the number of TH^+^ neurons in control mice of the same age was up to 3-fold. These latter differences are likely due to differences in the selection of the guard zone and disector height, whereas sample frequency played only a minor role.

These results raise the interesting question of how stereological estimates using design-based 3D stereological methods could have a greater degree of precision than those based upon a complete determination of the number of TH^+^ in the entire structure. In theory, if the investigator evaluated PQ-treated and control mice in an identical manner, then these sources of variability should not contribute differentially to the estimations of the mean number of TH^+^ neurons in PQ-treated vs. control mice. In order to ensure that differential effects do not occur, investigators should employ a block random design so that all temporal factors that might affect measurement outcomes (i.e. date of sacrifice, order of tissue processing, sectioning, staining, and stereological evaluation) are equally/randomly distributed among the vehicle, PQ-treated and positive control groups. Furthermore, if the investigator is blinded to treatment, then other sources of potential bias would be randomly distributed among the treated and control groups.

It has been postulated that the reduction in TH^+^ neurons in PQ-treated mice is not due to reduced expression or altered antibody recognition of TH, but is instead attributable to an actual death of DA neurons. To support this hypothesis, researchers have double-stained DA neurons for both TH and Nissl substance. Some investigators have reported there was an equivalent reduction in the estimated total number of TH^+^-stained neurons and Nissl-stained neurons in the SNpc, whereas others have not (Table 3.6). Although there is a strong correlation between the estimated mean number of TH^+^-stained neurons in the SNpc and the total number of Nissl-stained cells (Figure 3.7), the agreement between the losses of DA neurons, estimated by the two techniques, is far from perfect. In our experience, the dark brown chromogen used to stain DA neurons obstructs the visualization of the Nissl-stained cells, making it difficult to achieve a reliable count of Nissl-stained neurons. When we stained alternate sections for the TH^+^-DAB-linked chromophore or Nissl [1], we found no evidence of a loss of DA neurons. Furthermore, when we used two different fluorescent chromophores to identify TH and Nissl substance in DA neurons, we found no evidence of a loss in the estimated number of DA neurons in PQ-treated mice.

**Evidence of DA neuronal cell death in PQ-treated mice**

Due to limitations of stereological methods that rely on phenotypic expression of TH to identify cell loss, direct evidence of cell death from histopathological examinations must take precedence in determining whether PQ kills DA neurons in the SNpc. A review of the published literature (Table 3.1) indicates that, aside from studies we conducted [1, 2], few studies have provided sufficient information on the neuropathological effects of PQ (Supplemental Table 4.1). Based on the scope and comprehensiveness of our studies, we conclude that it is implausible that DA neurons could have died without detection in PQ-treated mice in these studies.

**Neurochemistry**

When a significant number of DA neurons die in the SNpc, as is routinely seen in MPTP-treated mice, a concomitant reduction in striatal DA levels and increased DA turnover is typically observed [13-15]. In contrast, PQ-treated mice in our studies did not display neuronal cell death, reduced striatal DA levels, or increased DA turnover. Overall, the administration of PQ to mice at maximum tolerated doses for different durations of time had no effect on DA levels or DA turnover. However, a detailed time course study may be warranted given inconsistent results among the published studies.

**Conclusions**

The results from the current investigation, coupled with data from the published literature, indicate that significant disagreement regarding the neurotoxicity of paraquat remains [16-23]. Our experiments have clearly shown that PQ, administered at maximum tolerated doses, does not induce any neuropathogenic effects using three methods of quantitation performed by three independent groups of investigators who were blinded to treatment for a series of neuropathological indices evaluated at two ages in sub-strains of male C57BL/6 mice housed under different conditions in two laboratories. In contrast, the neurotoxin, MPTP, when administered to mice housed and treated under identical conditions, produced consistent evidence of neurodegenerative changes in all parameters investigated and for all scenarios evaluated.

We have identified some of the limitations in the application of stereology to the SNpc, especially if block random procedures for controlling potential systematic differences in tissue processing, immunostaining and stereological factors are not utilized. This issue has become especially apparent in our review of certain published studies in which the combined biological and methodological variation was reported to be less than 10% in 3D stereological studies that evaluated the loss DA neurons in PQ-treated mice. Furthermore, as other researchers have suggested [24], the lack of investigator blinding in animal bioassays raises the possibility of observer bias affecting outcomes. Finally, uncertainty around the spontaneous variation in estimates of the number of TH^+^ neurons in the left and right SNpc of individual animals obtained from several commercial suppliers generate additional concerns about the reliability of stereological findings [25, 26]}. Thus as Prasad et al. [26] has suggested, biological and/or methodological variability may render stereology unsuitable for assessing the neurotoxicity potential of agents suspected of being linked to Parkinson’s disease (PD). Until there is a clearer understanding of the impact of these limitations on experimental outcomes, it is not likely that significant progress will be made in developing models of PD, or in the use of these models as tools to evaluate neuroprotective strategies.

**S3 Fig 1:** Percent change in the mean number of TH^+^ neurons in the SNpc of a PQ-treated group compared to the mean in the corresponding control group plotted against the coefficient of variation (CV) measured in the control group. (Current study: Green markers, bolded outlines).

**S3 Fig 2:** Mean number of TH^+^ neurons in successive blocks of serial sections through the SNpc of C57BL/6J male mice.

**S3. Fig 3:** Correlation between the estimated total number of TH^+^ neurons in the left vs. the right SNpc of 9 week old control C57BL/6J male mice (Data from Breckenridge et al., 2013 [1]).


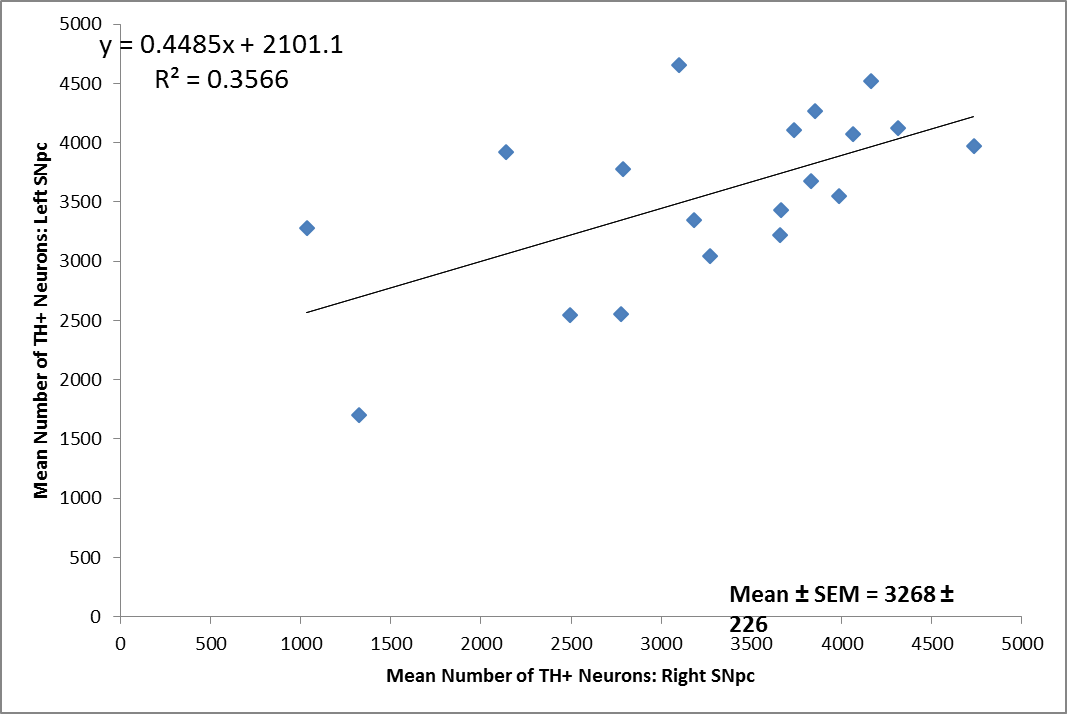


**Mean ± SEM = 3567 ± 170**

**S3 Fig 4:** Mean number of TH^+^ neurons in the SNpc of 24 groups of control, 6-13 week old C57BL/6 male mice reported in 19 studies^1^. Group means were estimated were based on 3D, design-based stereology of DAB-stained TH^+^ neurons (Mean ± SEM = 11133 ± 533).

^1^Study number 1G1, 3, 6, 10, 14, 20, 23, 24, 25, 26, 27, 30, 31, 37, 38, 41, 47, 48 and 50. In some studies (#3, 23, 24, 25, 37) the results from two separate control groups are included.

**S3 Fig 5:** Estimated mean number (top) and CV (bottom) for TH^+^ neurons counted through the entire Z-depth of a nominal 40 μm thick section of the SNpc in 9 week-old, untreated C57BL/6J male mice (Data from Breckenridge et al., 2013 [1]).

**S3 Fig 6:** The effect of disector height on the estimated total number of the TH^+^ neurons in the SNpc of untreated 9-week old male C57BL/6J male mice (Data from Breckenridge et al., 2013 [1]).

**S3 Fig 7:** Mean difference in the number of TH^+^ neurons in the SNpc of PQ treated C57BL/6J male mice compared to controls vs. the mean difference in the number of Nissl positive neurons in PQ-treated mice compared to controls (positive evaluations based upon statistically significant reductions in TH^+^ neurons in PQ-treated mice compared to controls).

S3 Table 1: Stereological and neuropathological characteristics of published studies on paraquat in male mice.

| **First Author (Date)** | **Study Number** | **SNpc: Stereology/Pathology of Dopaminergic Neurons, Microglia and Astrocytes Evidence of a PQ, Treatment-Related Effect** | | | | | | | **Striatal Pathology** |
| --- | --- | --- | --- | --- | --- | --- | --- | --- | --- |
|  |  | **Decreased No. of TH^+^ Neurons** | **Decreased No. of Nissl Neurons** | **Silver/Fluoro Jade B Stains** | **Caspase 3 Stain** | **TUNEL Stain** | **Microglia Activation** | **Astrogliosis** | **Decreased TH Stain** |
| Smeyne (2016) This study St Jude | 1a | **No** |  |  |  |  | **No** |  |  |
| Smeyne (2016) This study c EPL/ToxPath | 1b | **No** | **No** | **No** | **No** | **No** | **No** | **No** | **No** |
| Barlow (2004) [27] | 2 | **No** | **No** |  |  |  |  |  |  |
| Breckenridge (2013) [1] | 3 | **No** | **No** | **No** | **No** | **No** | **No** | **No** | **No** |
| Brooks (1999) [18] | 4 | **Yes** |  |  |  |  |  |  | **Yes** |
| Chen (2008) [28] | 5 | **Yes** |  |  |  |  |  |  |  |
| Choi (2006a) [29] | 6 | **Yes** | **Yes** |  |  |  |  |  |  |
| Choi (2006b) [30] | 7 |  | **Yes** |  |  |  |  |  |  |
| Choi (2010) [31] | 8 | **Yes** |  |  |  |  |  |  |  |
| Cristovao (2009) [32] | 9 | **Yes** | **Yes** |  |  |  | **Yes** |  |  |
| Fei (2008) [33] | 10 | **Yes** | **Yes** |  |  |  |  |  |  |
| Fernagut (2007) [8] | 11 | **Yes** |  |  |  |  | **No** |  | **Yes** |
| Gollamudi (2012) [34] | 12 | **Yes** |  |  |  |  |  |  |  |
| Jiao (2012) [19] | 13 | **Yes** |  |  |  |  |  |  |  |
| Kang (2009) [35] | 14 | **Yes** |  |  |  |  |  |  |  |
| Kang (2010) [36] | 15 | **Yes** |  |  |  |  |  |  |  |
| Khwaja (2007) [37] | 16 | **Yes** | **Yes** |  |  |  |  |  |  |
| Li (2005) [38] | 17 | **Yes** |  |  |  |  |  |  |  |
| Li (2012) [12] | 18 | **Yes** |  |  |  | **Yes** |  |  |  |
| Mangano (2009) [39] | 19 | **Yes** | **Yes** |  |  |  | **Yes** |  |  |
| Mangano (2011) [40] | 20 | **Yes** | **No** |  |  |  | **Yes** |  |  |
| Mangano (2012) [41] | 21 | **Yes** |  |  |  |  |  |  |  |
| Manning-Bog (2003) [42] | 22 | **Yes** | **Yes** | **Yes** |  |  |  |  |  |
| McCormack (2002) [17] | 23 | **Yes** | **Yes** | **Yes** |  |  | **Yes** |  |  |
| McCormack (2003) [43] | 24 | **Yes** | **Yes** |  |  |  |  |  |  |
| McCormack (2005) [44] | 25 | **Yes** | **Yes** |  |  |  |  |  |  |
| McCormack (2006) [45] | 26 | **Yes** | **Yes** |  |  |  |  |  |  |
| Minnema (2014) [2] | 27 | **No** | **No** | **No** | **No** | **No** | **No** | **No** | **No** |
| Mitra (2011) [46] | 28 | **Yes** | **Yes** |  |  |  | **Yes** |  |  |
| Norris (2007) [47] | 29 | **No** |  |  |  |  |  |  |  |
| Peng (2004) [3] | 30 | **Yes** |  |  |  |  |  |  |  |
| Peng (2005) [6] | 31 | **Yes** |  |  |  |  |  |  |  |
| Peng (2006) [48] | 32 | **Yes** |  |  |  |  |  |  |  |
| Peng (2007) [5] | 33 | **Yes** |  |  |  |  |  |  |  |
| Peng (2010) [4] | 34 | **Yes** |  |  |  |  |  |  |  |
| Prakash (2013) [49] | 35 | **Yes** |  |  |  |  |  |  |  |
| Prasad (2009) [50] | 36 |  |  |  |  |  |  |  | **No** |
| Purisai (2007) [51] | 37 | **Yes** |  |  |  |  | **Yes** | **Yes** |  |
| Rappold (2011) [52] | 38 | **Yes** | **Yes** |  |  |  |  |  | **No/Yes** |
| Reeves (2003) [53] | 39 |  |  |  |  |  |  |  | **Yes** |
| Ren (2009) [54] | 40 | **Yes** |  |  |  |  |  |  |  |
| Rojo (2007) [55] | 41 | **No** |  |  |  |  |  |  | **No** |
| Srivastava (2012) [11] | 42 | **Yes** |  | **Yes** |  |  |  |  |  |
| Su (2015) [56] | 43 | **Yes** |  |  |  |  |  |  |  |
| Thiruchelvam (2000a) [57] | 44 |  |  |  |  |  |  |  | **No** |
| Thiruchelvam (2000b) [58] | 45 | **No** |  |  |  |  |  | **No** | **No** |
| Thiruchelvam (2002) [59] | 46 | **Yes** | **No** |  |  |  |  |  |  |
| Thiruchelvam (2003) [60] | 47 | **Yes** |  |  |  |  |  |  | **No/Yes** |
| Watson (2013) [9] | 48 | **No** | **No** |  |  |  | **Yes** |  | **No** |
| Yin (2011) [61] | 49 | **Yes** |  |  |  |  |  |  |  |
| Zhou (2011) [62] | 50 | **Yes** |  |  |  |  |  |  |  |
| **Number of Blinded Positive Study** | | 8 | 4 | **0** | **0** | 1 | 1 | 0 | 1 |
| **Number of Unblinded Positive Study** | | 30 | 9 | **3** | **3** | 0 | 6 | 1 | 2 |
| **Number of Blinded Negative Study** | | 6 | 5 | 3 | 0 | 3 | 5 | 3 | 4 |
| **Number of Unblinded Negative Study** | | 3 | 2 | 0 | 0 | 0 | 0 | 1 | 4 |
| **Inconsistent Results: No/Yes** | | 0 | 0 | 0 | 0 | 0 | 0 | 0 | 2 |
| **Number Not Evaluated** | | 4 | 31 | 45 | 48 | 47 | 39 | 46 | 38 |

S3 Table 2: Stereological data from published studies on paraquat in male mice.

S3 Table 3: Asymmetry in the estimated number of TH^+^ neurons between the left SNpc vs. the right SNpc of 9 week old, control C57BL/6J male mice (data from Breckenridge et al. [1]).

S3 Table 4: Variability in the stereologist’s judgment (contour drawing, TH^+^ neuron identification) and the stereology method (counting frame placement) in estimating the total number of TH^+^ neurons in the SNpc (left + right side combined) of two control 9-week old, male C57BL/6J mice. The assessment was repeated on several occasions by a stereologist who was blinded to treatment (Guard zone = 0; Disector Height = full depth of section; data from Minnema et al., 2014 [2]).

S3 Table 5: Effect of the number of 40 μm (nominal) thick sections evaluated (3D stereology; Guard zone = 2 μm; Disector height = 4 μm) on the estimated total number of TH^+^ neurons (left + right SNpc combined) in 9 week old control C57BL/6J male mice (data from Breckenridge et al. [1]).

S3 Table 6: Published studies that used bothTH^+^ and Nissl to label and calculate the mean number of neurons of each type in the SNpc of male mice.

| **First Author (Date)[Reference]** | **Study Number** | **SNpc: Decreased Number of TH+ Neurons** | **PQ Dose (mg/kg)** | **Number of Doses** | **Control: Mean TH^+^ Neurons** | **Paraquat: Mean TH^+^ Neurons** | **TH^+^ Difference (PQ-Control)** | **Control: Mean Nissl Positive (TH^+^ + TH^-^)** | **Paraquat: Mean Nissl Positive (TH^+^ + TH^-^)** | **Nissl+ Difference (PQ-Control)** |
| --- | --- | --- | --- | --- | --- | --- | --- | --- | --- | --- |
| Barlow (2004) [27] | 2 | **No: GD 10-17; PND 48-55** | GD (0.3); PND (5) | 8 | 5961 | 5346 | -615 | 8807 | 8423 | -384 |
| Breckenridge (2013) [1] | 3 | **No** | 10 | 3 | 11700 | 10965 | -735 | 17281 | 16217 | -1064 |
| Breckenridge (2013) [1] | 3 | **No** | 10 | 3 | 11700 | 10106 | -1594 | 17281 | 15890 | -1391 |
| Breckenridge (2013) [1] | 3 | **No** | 15 | 1 | 11700 | 10210 | -1490 | 17281 | 16205 | -1076 |
| Breckenridge (2013) [1] | 3 | **No** | 15 | 2 | 11700 | 10852 | -848 | 17281 | 16889 | -392 |
| Breckenridge (2013) [1] | 3 | **Yes** | 15 | 3 | 11700 | 8020 | -3680 | 17281 | 14656 | -2625 |
| Breckenridge (2013) [1] | 3 | **No: DAB** | 10 | 3 | 9888 | 9518 | -370 | 16463 | 17672 | 1209 |
| Breckenridge (2013) [1] | 3 | **No: DAB** | 15 | 3 | 9888 | 8631 | -1257 | 16463 | 17571 | 1108 |
| Breckenridge (2013) [1] | 3 | **No: DAB** | 25 | 3 | 9888 | 8748 | -1140 | 16463 | 15503 | -960 |
| Breckenridge (2013) [1] | 3 | **No: ALEXA/DAPI** | 10 | 3 | 16615 | 16270 | -345 | 29637 | 28225 | -1412 |
| Breckenridge (2013) [1] | 3 | **No: ALEXA/DAPI** | 15 | 3 | 16615 | 14541 | -2074 | 29637 | 26852 | -2785 |
| Breckenridge (2013) [1] | 3 | **No: ALEXA/DAPI** | 25 | 3 | 16615 | 14470 | -2145 | 29637 | 27282 | -2355 |
| Minnema (2014) [2] | 27 | **No: Males - 3 Months** | 1.7 | > 91 d | 15173 | 15624 | 451 | 22663 | 23223 | 560 |
| Minnema (2014) [2] | 27 | **No: Males - 3 Months** | 10.2 | > 91 d | 15173 | 14794 | -379 | 22663 | 22085 | -578 |
| Fei (2008) [33] | 10 | **Yes** | 10 | 3 | 5900 | 4075 | -1825 | 7143 | 5143 | -2000 |
| Khwaja (2007) [37] | 16 | **Yes** | 10 | 3 | 6185 | 4615 | -1570 | 8000 | 6000 | -2000 |
| Manning-Bog (2003) [42] | 22 | **Yes** | 10 | 3 | 12084 | 9462 | -2622 | 16468 | 13522 | -2946 |
| McCormack (2002) [17] | 23 | **Yes** | 10 | 3 | 12321 | 8422 | -3899 | 16534 | 12464 | -4070 |
| McCormack (2003) [43] | 24 | **Yes** | 10 | 3 | 12476 | 9284 | -3192 | 17042 | 12646 | -4396 |
| McCormack (2003) [43] | 24 | **Yes** | 10 | 3 | 12519 | 9481 | -3038 | 17037 | 12741 | -4296 |
| McCormack (2005) [44] | 25 | **Yes: 8 Weeks** | 10 x 1: D7 | 1 | 11770 | 11511 | -259 | 15808 | 15521 | -287 |
| McCormack (2005) [44] | 25 | **Yes: 8 Weeks** | 10 x 2:D1 | 2 | 11770 | 11070 | -700 | 15808 | 14955 | -853 |
| McCormack (2005) [44] | 25 | **Yes: 8 Weeks** | 10 x 2:D2 | 2 | 11770 | 10215 | -1555 | 15808 | 14292 | -1516 |
| McCormack (2005) [44] | 25 | **Yes: 8 Weeks** | 10 x 2:D4 | 2 | 11770 | 8504 | -3266 | 15808 | 12699 | -3109 |
| McCormack (2005) [44] | 25 | **Yes: 8 Weeks** | 10 x 2:D7 | 2 | 11770 | 8866 | -2904 | 15808 | 12237 | -3571 |
| McCormack (2005) [44] | 25 | **Yes: 8 Weeks** | 10 x 3:D7 | 3 | 12369 | 9277 | -3092 | 15201 | 12288 | -2913 |
| McCormack (2006) [45] | 26 | **Yes** | 10 | 3 | 12852 | 8889 | -3963 | 15852 | 11407 | -4445 |
| Purisai (2007) [51] | 37 | **No: Single PQ dose** | 10 | 1 | 12937 | 12107 | -830 | 14842 | 14583 | -259 |
| Thiruchelvam (2002) [59] | 46 | **Yes: PND 5-19** | 0.3: (PND 5-19) | 15 | 11833 | 9915 | -1918 | 15389 | 13174 | -2215 |
| Thiruchelvam (2002) [59] | 46 | **Yes: PND + Adult (6.5 Months)** | 0.3 PND/10 Adult | 15 +/7 | 12194 | 7222 | -4972 | 15861 | 10833 | -5028 |
| Thiruchelvam (2002) [59] | 46 | **Yes: Adult Only (6.5 Months)** | 10 | 7 | 11556 | 8833 | -2723 | 15074 | 12203 | -2871 |
| Watson (2013) [9] | 48 | **No: 8-13 Weeks** | 10 | 1 | 13704 | 12370 | -1334 | 26519 | 26222 | -297 |

S3 Table 7: Association between neurochemisty and neuropathology in published studies on paraquat in male mice

**REFERENCES**

1. Breckenridge CB, Sturgess NC, Butt M, Wolf JC, Zadory D, Beck M, et al. Pharmacokinetic, neurochemical, stereological and neuropathological studies on the potential effects of paraquat in the substantia nigra pars compacta and striatum of male C57BL/6J mice. Neurotoxicology. 2013;37:1-14. Epub 2013/03/26. doi: 10.1016/j.neuro.2013.03.005. PubMed PMID: 23523781.

2. Minnema DJ, Travis KZ, Breckenridge CB, Sturgess NC, Butt M, Wolf JC, et al. Dietary administration of paraquat for 13 weeks does not result in a loss of dopaminergic neurons in the substantia nigra of C57BL/6J mice. Regulatory Toxicology and Pharmacology. 2014;68(2):250–8. doi: 10.1016/j.yrtph.2013.12.010.

3. Peng J, Mao XO, Stevenson FF, Hsu M, Andersen JK. The herbicide paraquat induces dopaminergic nigral apoptosis through sustained activation of the JNK pathway. J Biol Chem. 2004;279(31):32626-32. Epub 2004 May 20.

4. Peng J, Oo ML, Andersen JK. Synergistic effects of environmental risk factors and gene mutations in Parkinson's disease accelerate age-related neurodegeneration. Journal of Neurochemistry. 2010;115(6):1363-73. Epub 2010/11/03. doi: 10.1111/j.1471-4159.2010.07036.x. PubMed PMID: 21039522.

5. Peng J, Peng L, Stevenson FF, Doctrow SR, Andersen JK. Iron and paraquat as synergistic environmental risk factors in sporadic Parkinson's disease accelerate age-related neurodegeneration. Journal of Neuroscience. 2007;27(26):6914-22. Epub 2007/06/29. doi: 27/26/6914 [pii]

10.1523/JNEUROSCI.1569-07.2007 [doi]. PubMed PMID: 17596439.

6. Peng J, Stevenson FF, Doctrow SR, Andersen JK. Superoxide dismutase/catalase mimetics are neuroprotective against selective paraquat-mediated dopaminergic neuron death in the substantial nigra: implications for Parkinson disease. J Biol Chem. 2005;280(32):29194-8. Epub 2005/06/11. doi: M500984200 [pii]

10.1074/jbc.M500984200 [doi]. PubMed PMID: 15946937.

7. Baquet ZC, Williams D, Brody J, Smeyne RJ. A comparison of model-based (2D) and design-based (3D) stereological methods for estimating cell number in the substantia nigra pars compacta (SNpc) of the C57BL/6J Mouse. Neuroscience. 2009;161(4):1082-90. Epub 2009/04/21. doi: S0306-4522(09)00637-X [pii]

10.1016/j.neuroscience.2009.04.031 [doi]. PubMed PMID: 19376196.

8. Fernagut PO, Hutson CB, Fleming SM, Tetreaut NA, Salcedo J, Masliah E, et al. Behavioral and histopathological consequences of paraquat intoxication in mice: effects of alpha-synuclein over-expression. Synapse (New York, NY). 2007;61(12):991-1001. Epub 2007/09/20. doi: 10.1002/syn.20456. PubMed PMID: 17879265; PubMed Central PMCID: PMCPMC3097512.

9. Watson MB, Nobuta H, Abad C, Lee SK, Bala N, Zhu C, et al. PACAP deficiency sensitizes nigrostriatal dopaminergic neurons to paraquat-induced damage and modulates central and peripheral inflammatory activation in mice. Neuroscience. 2013;240:277-86. Epub 2013/03/19. doi: 10.1016/j.neuroscience.2013.03.002. PubMed PMID: 23500093; PubMed Central PMCID: PMCPMC3637876.

10. Manning-Bog AB, McCormack AL, Li J, Uversky VN, Fink AL, Di Monte DA. The herbicide paraquat causes up-regulation and aggregation of alpha-synuclein in mice: paraquat and alpha-synuclein. J Biol Chem. 2002;277(3):1641-4. Epub 2001/11/15. doi: 10.1074/jbc.C100560200. PubMed PMID: 11707429.

11. Srivastava G, Dixit A, Yadav S, Patel DK, Prakash O, Singh MP. Resveratrol potentiates cytochrome P450 2d22-mediated neuroprotection in maneb- and paraquat-induced parkinsonism in the mouse. Free Radical Biology and Medicine. 2012;52(8):1294-306. Epub 2012/02/16. doi: 10.1016/j.freeradbiomed.2012.02.005. PubMed PMID: 22334051.

12. Li H, Wu S, Wang Z, Lin W, Zhang C, Huang B. Neuroprotective effects of tert-butylhydroquinone on paraquat-induced dopaminergic cell degeneration in C57BL/6 mice and in PC12 cells. Archives of Toxicology. 2012;86(11):1729-40. Epub 2012/09/18. doi: 10.1007/s00204-012-0935-y. PubMed PMID: 22983789.

13. Bezard E, Jaber M, Gonon F, Boireau A, Bloch B, Gross CE. Adaptive changes in the nigrostriatal pathway in response to increased 1-methyl-4-phenyl-1,2,3,6-tetrahydropyridine-induced neurodegeneration in the mouse. European Journal of Neuroscience. 2000;12(8):2892-900.

14. Przedborski S, Jackson-Lewis V, Yokoyama R, Shibata T, Dawson VL, Dawson TM. Role of neuronal nitric oxide in 1-methyl-4-phenyl-1,2,3,6-tetrahydropyridine (MPTP)-induced dopaminergic neurotoxicity. Proc Natl Acad Sci U S A. 1996;93(10):4565-71.

15. Stephenson D, Ramirez A, Long J, Barrezueta N, Hajos-Korcsok E, Matherne C, et al. Quantification of MPTP-induced dopaminergic neurodegeneration in the mouse substantia nigra by laser capture microdissection. J Neurosci Methods. 2007;159(2):291-9. Epub 2006/09/05. doi: 10.1016/j.jneumeth.2006.07.027. PubMed PMID: 16949674.

16. Bove J, Prou D, Perier C, Przedborski S. Toxin-induced models of Parkinson's disease. NeuroRx. 2005;2(3):484-94. Epub 2006/01/04. doi: 10.1602/neurorx.2.3.484. PubMed PMID: 16389312; PubMed Central PMCID: PMC1144492.

17. McCormack AL, Thiruchelvam M, Manning-Bog AB, Thiffault C, Langston JW, Cory-Slechta DA, et al. Environmental risk factors and Parkinson's disease: Selective degeneration of nigral dopaminergic neurons caused by the herbicide paraquat. Neurobiology of Disease. 2002;10(2):119-27.

18. Brooks AI, Chadwick CA, Gelbard HA, Cory-Slechta DA, Federoff HJ. Paraquat elicited neurobehavioral syndrome caused by dopaminergic neuron loss. Brain Res. 1999;823(1-2):1-10.

19. Jiao Y, Lu L, Williams RW, Smeyne RJ. Genetic dissection of strain dependent paraquat-induced neurodegeneration in the substantia nigra pars compacta. PLoS One. 2012;7(1):e29447. Epub 2012/02/01. doi: 10.1371/journal.pone.0029447. PubMed PMID: 22291891; PubMed Central PMCID: PMC3265472.

20. Miller GW. Paraquat: the red herring of Parkinson's disease research. Toxicol Sci. 2007;100(1):1-2. Epub 2007/10/16. doi: 10.1093/toxsci/kfm223. PubMed PMID: 17934192.

21. Minnema DJ, Travis KZ, Breckenridge CB, Sturgess NC, Butt M, Wolf JC, et al. Dietary administration of paraquat for 13weeks does not result in a loss of dopaminergic neurons in the substantia nigra of C57BL/6J mice. Regulatory Toxicology and Pharmacology. 2014;68(2):250-8. Epub 2014/01/07. doi: 10.1016/j.yrtph.2013.12.010. PubMed PMID: 24389362.

22. Jones BC, Huang X, Mailman RB, Lu L, Williams RW. The perplexing paradox of paraquat: the case for host-based susceptibility and postulated neurodegenerative effects. J Biochem Mol Toxicol. 2014;28(5):191-7. Epub 2014/03/07. doi: 10.1002/jbt.21552. PubMed PMID: 24599642.

23. Jones BC, Lu L, Williams RW, Unger EL, Yin L. Response to Breckenridge et al. (2013). Neurotoxicology. 2013;38:23-4. Epub 2013/06/04. doi: 10.1016/j.neuro.2013.05.010. PubMed PMID: 23727076.

24. Bello S, Krogsboll LT, Gruber J, Zhao ZJ, Fischer D, Hrobjartsson A. Lack of blinding of outcome assessors in animal model experiments implies risk of observer bias. J Clin Epidemiol. 2014;67(9):973-83. Epub 2014/06/29. doi: 10.1016/j.jclinepi.2014.04.008. PubMed PMID: 24972762.

25. Pioli EY, Dovero S, Bioulac BH, Gross CE, Bezard E. Asymmetrically lesioned mesencephalon in healthy rodents: call for caution. Brain Res. 2004;1022(1-2):251-3. Epub 2004/09/09. doi: 10.1016/j.brainres.2004.07.014. PubMed PMID: 15353237.

26. Prasad K, Richfield E. Sporadic midbrain dopamine neuron abnormalities in laboratory mice. Neurobiol Dis. 2008;32(2):262-72. doi: 10.1016/j.nbd.2008.07.007.

27. Barlow BK, Richfield EK, Cory-Slechta DA, Thiruchelvam M. A fetal risk factor for Parkinson's disease. Developmental Neuroscience. 2004;26(1):11-23. Epub 2004/10/29. doi: DNE2004026001011 [pii]

10.1159/000080707 [doi]. PubMed PMID: 15509894.

28. Chen P, Chen Z, Li A, Lou XC, Wu XK, Zhao CJ, et al. Catalytic metalloporphyrin protects against paraquat neurotoxicity in vivo. Biomedical and Environmental Sciences. 2008;21(3):233-8. Epub 2008/08/22. doi: 10.1016/s0895-3988(08)60035-5. PubMed PMID: 18714822.

29. Choi HS, An JJ, Kim SY, Lee SH, Kim DW, Yoo KY, et al. PEP-1-SOD fusion protein efficiently protects against paraquat-induced dopaminergic neuron damage in a Parkinson disease mouse model. Free Radical Biology and Medicine. 2006;41(7):1058-68. Epub 2006/09/12. doi: S0891-5849(06)00390-X [pii]

10.1016/j.freeradbiomed.2006.06.006 [doi]. PubMed PMID: 16962931.

30. Choi HS, Lee SH, Kim SY, An JJ, Hwang SI, Kim DW, et al. Transduced Tat-alpha-synuclein protects against oxidative stress in vitro and in vivo. Journal of Biochemistry and Molecular Biology. 2006;39(3):253-62. Epub 2006/06/08. PubMed PMID: 16756753.

31. Choi WS, Abel G, Klintworth H, Flavell RA, Xia Z. JNK3 Mediates Paraquat- and Rotenone-Induced Dopaminergic Neuron Death. Journal of Neuropathology and Experimental Neurology. 2010;69(5):511-20. Epub 2010/04/27. doi: 10.1097/NEN.0b013e3181db8100. PubMed PMID: 20418776.

32. Cristovao AC, Choi D, Baltazar G, Beal F, Kim YS. The role of NADPH oxidase 1-derived reactive oxygen species in paraquat-mediated dopaminergic cell death. Antioxidants and Redox Signaling. 2009;11(9):2105-18. Epub 2009/05/20. doi: 10.1089/ARS.2009.2459 [doi]. PubMed PMID: 19450058.

33. Fei Q, McCormack AL, Di Monte DA, Ethell DW. Paraquat neurotoxicity is mediated by a Bak-dependent mechanism. J Biol Chem. 2008;283(6):3357-64. Epub 2007/12/07. doi: 10.1074/jbc.M708451200. PubMed PMID: 18056701.

34. Gollamudi S, Johri A, Calingasan NY, Yang L, Elemento O, Beal MF. Concordant signaling pathways produced by pesticide exposure in mice correspond to pathways identified in human Parkinson's disease. PLoS One. 2012;7(5):e36191. Epub 2012/05/09. doi: 10.1371/journal.pone.0036191. PubMed PMID: 22563483; PubMed Central PMCID: PMC3341364.

35. Kang MJ, Gil SJ, Koh HC. Paraquat induces alternation of the dopamine catabolic pathways and glutathione levels in the substantia nigra of mice. Toxicology Letters. 2009;188(2):148-52. Epub 2009/05/19. doi: 10.1016/j.toxlet.2009.03.026. PubMed PMID: 19446248.

36. Kang MJ, Gil SJ, Lee JE, Koh HC. Selective vulnerability of the striatal subregions of C57BL/6 mice to paraquat. Toxicology Letters. 2010;195(2-3):127-34. Epub 2010/03/24. doi: 10.1016/j.toxlet.2010.03.011. PubMed PMID: 20307631.

37. Khwaja M, McCormack A, McIntosh JM, Di Monte DA, Quik M. Nicotine partially protects against paraquat-induced nigrostriatal damage in mice; link to alpha6beta2* nAChRs. Journal of Neurochemistry. 2007;100(1):180-90. Epub 2007/01/18. doi: JNC4177 [pii]

10.1111/j.1471-4159.2006.04177.x [doi]. PubMed PMID: 17227438.

38. Li X, Yin J, Cheng CM, Sun JL, Li Z, Wu YL. Paraquat induces selective dopaminergic nigrostriatal degeneration in aging C57BL/6 mice. Chinese Medical Journal (English Edition). 2005;118(16):1357-61. Epub 2005/09/15. PubMed PMID: 16157030.

39. Mangano EN, Hayley S. Inflammatory priming of the substantia nigra influences the impact of later paraquat exposure: Neuroimmune sensitization of neurodegeneration. Neurobiology of Aging. 2009;30(9):1361-78. Epub 2008/01/12. doi: S0197-4580(07)00449-6 [pii]

10.1016/j.neurobiolaging.2007.11.020 [doi]. PubMed PMID: 18187236.

40. Mangano EN, Peters S, Litteljohn D, So R, Bethune C, Bobyn J, et al. Granulocyte macrophage-colony stimulating factor protects against substantia nigra dopaminergic cell loss in an environmental toxin model of Parkinson's disease. Neurobiol Dis. 2011;43(1):99-112. Epub 2011/03/08. doi: 10.1016/j.nbd.2011.02.011. PubMed PMID: 21377529.

41. Mangano EN, Litteljohn D, So R, Nelson E, Peters S, Bethune C, et al. Interferon-gamma plays a role in paraquat-induced neurodegeneration involving oxidative and proinflammatory pathways. Neurobiology of Aging. 2012;33(7):1411-26. Epub 2011/04/13. doi: 10.1016/j.neurobiolaging.2011.02.016. PubMed PMID: 21482445.

42. Manning-Bog AB, McCormack AL, Purisai MG, Bolin LM, Di Monte DA. Alpha-synuclein overexpression protects against paraquat-induced neurodegeneration. Journal of Neuroscience. 2003;23(8):3095-9. Epub 2003/04/30. doi: 23/8/3095 [pii]. PubMed PMID: 12716914.

43. McCormack AL, Di Monte DA. Effects of L-dopa and other amino acids against paraquat-induced nigrostriatal degeneration. Journal of Neurochemistry. 2003;85(1):82-6. Epub 2003/03/19. PubMed PMID: 12641729.

44. McCormack AL, Atienza JG, Johnston LC, Andersen JK, Vu S, Di Monte DA. Role of oxidative stress in paraquat-induced dopaminergic cell degeneration. Journal of Neurochemistry. 2005;93(4):1030-7. PubMed PMID: 15857406.

45. McCormack AL, Atienza JG, Langston JW, Di Monte DA. Decreased susceptibility to oxidative stress underlies the resistance of specific dopaminergic cell populations to paraquat-induced degeneration. Neuroscience. 2006;141(2):929-37. Epub 2006/05/09. doi: 10.1016/j.neuroscience.2006.03.069. PubMed PMID: 16677770.

46. Mitra S, Chakrabarti N, Bhattacharyya A. Differential regional expression patterns of alpha-synuclein, TNF-alpha, and IL-1beta; and variable status of dopaminergic neurotoxicity in mouse brain after Paraquat treatment. J Neuroinflammation. 2011;8(1):163. Epub 2011/11/25. doi: 10.1186/1742-2094-8-163. PubMed PMID: 22112368.

47. Norris EH, Uryu K, Leight S, Giasson BI, Trojanowski JQ, Lee VM. Pesticide exposure exacerbates alpha-synucleinopathy in an A53T transgenic mouse model. American Journal Of Pathology. 2007;170(2):658-66. Epub 2007/01/27. doi: 170/2/658 [pii]

10.2353/ajpath.2007.060359 [doi]. PubMed PMID: 17255333.

48. Peng J, Xie L, Stevenson FF, Melov S, Di Monte DA, Andersen JK. Nigrostriatal dopaminergic neurodegeneration in the weaver mouse is mediated via neuroinflammation and alleviated by minocycline administration. Journal of Neuroscience. 2006;26(45):11644-51. Epub 2006/11/10. doi: 10.1523/jneurosci.3447-06.2006. PubMed PMID: 17093086.

49. Prakash J, Yadav SK, Chouhan S, Singh SP. Neuroprotective Role of Withania somnifera Root Extract in Maneb-Paraquat Induced Mouse Model of Parkinsonism. Neurochemical Research. 2013;38(5):972-80. Epub 2013/02/23. doi: 10.1007/s11064-013-1005-4. PubMed PMID: 23430469.

50. Prasad K, Tarasewicz E, Mathew J, Strickland PA, Buckley B, Richardson JR, et al. Toxicokinetics and toxicodynamics of paraquat accumulation in mouse brain. Experimental Neurology. 2009;215(2):358-67. Epub 2008/12/17. doi: S0014-4886(08)00429-9 [pii]

10.1016/j.expneurol.2008.11.003 [doi]. PubMed PMID: 19084006.

51. Purisai MG, McCormack AL, Cumine S, Li J, Isla MZ, Di Monte DA. Microglial activation as a priming event leading to paraquat-induced dopaminergic cell degeneration. Neurobiol Dis. 2007;25(2):392-400. PubMed PMID: 17166727.

52. Rappold PM, Cui M, Chesser AS, Tibbett J, Grima JC, Duan L, et al. Paraquat neurotoxicity is mediated by the dopamine transporter and organic cation transporter-3. Proc Natl Acad Sci U S A. 2011;108(51):20766-71. Epub 2011/12/07. doi: 10.1073/pnas.1115141108. PubMed PMID: 22143804; PubMed Central PMCID: PMCPmc3251116.

53. Reeves R, Thiruchelvam M, Baggs RB, Cory-Slechta DA. Interactions of paraquat and triadimefon: behavioral and neurochemical effects. Neurotoxicology. 2003;24(6):839-50. Epub 2003/11/26. doi: 10.1016/s0161-813x(03)00057-3. PubMed PMID: 14637379.

54. Ren JP, Zhao YW, Sun XJ. Toxic influence of chronic oral administration of paraquat on nigrostriatal dopaminergic neurons in C57BL/6 mice Chinese Medical Journal (English Edition). 2009;122(19):2366-71.

55. Rojo AI, Cavada C, de Sagarra MR, Cuadrado A. Chronic inhalation of rotenone or paraquat does not induce Parkinson's disease symptoms in mice or rats. Experimental Neurology. 2007;208(1):120-6. Epub 2007/09/21. doi: S0014-4886(07)00300-7 [pii]

10.1016/j.expneurol.2007.07.022 [doi]. PubMed PMID: 17880941.

56. Su C, Niu P. Low doses of single or combined agrichemicals induces alpha-synuclein aggregation in nigrostriatal system of mice through inhibition of proteasomal and autophagic pathways. Int J Clin Exp Med. 2015;8(11):20508-15. Epub 2016/02/18. PubMed PMID: 26884967; PubMed Central PMCID: PMC4723812.

57. Thiruchelvam M, Brockel BJ, Richfield EK, Baggs RB, Cory-Slechta DA. Potentiated and preferential effects of combined paraquat and maneb on nigrostriatal dopamine systems: environmental risk factors for Parkinson's disease? Brain Res. 2000;873(2):225-34. Epub 2000/08/10. doi: S0006-8993(00)02496-3 [pii]. PubMed PMID: 10930548.

58. Thiruchelvam M, Richfield EK, Baggs RB, Tank AW, Cory-Slechta DA. The nigrostriatal dopaminergic system as a preferential target of repeated exposures to combined paraquat and maneb: implications for Parkinson's disease. Journal of Neuroscience. 2000;20(24):9207-14.

59. Thiruchelvam M, Richfield EK, Goodman BM, Baggs RB, Cory-Slechta DA. Developmental exposure to the pesticides paraquat and maneb and the Parkinson's disease phenotype. Neurotoxicology. 2002;23(4-5):621-33. Epub 2002/11/14. doi: S0161-813X(02)00092-X [pii]. PubMed PMID: 12428734.

60. Thiruchelvam M, McCormack A, Richfield EK, Baggs RB, Tank AW, Di Monte DA, et al. Age-related irreversible progressive nigrostriatal dopaminergic neurotoxicity in the paraquat and maneb model of the Parkinson's disease phenotype. Eur J Neurosci. 2003;18(3):589-600. Epub 2003/08/13. doi: 2781 [pii]. PubMed PMID: 12911755.

61. Yin L, Lu L, Prasad K, Richfield EK, Unger EL, Xu J, et al. Genetic-based, differential susceptibility to paraquat neurotoxicity in mice. Neurotoxicology and Teratology. 2011;33(3):415-21. Epub 2011/03/05. doi: 10.1016/j.ntt.2011.02.012. PubMed PMID: 21371552.

62. Zhou H, Huang C, Tong J, Xia XG. Early Exposure to Paraquat Sensitizes Dopaminergic Neurons to Subsequent Silencing of PINK1 Gene Expression in Mice. Int J Biol Sci. 2011;7(8):1180-7. Epub 2011/11/02. PubMed PMID: 22043175; PubMed Central PMCID: PMC3204408.

63. Lermontova NN, Solyakov LS, Bachurin SO, Tkachenko SE, Serkova TP, Petrova LN, et al. Ability of 1-methyl-4-phenyl-1,2,3,6-tetrahydropyridine and some other pyridine derivatives to cause parkinsonism. Bulletin of Experimental Biology and Medicine. 1989;107(6):815-7. Epub 1989/06/01. doi: 10.1007/BF00840748. PubMed PMID: 2790166.

64. Litteljohn D, Mangano EN, Hayley S. Cyclooxygenase-2 deficiency modifies the neurochemical effects, motor impairment and co-morbid anxiety provoked by paraquat administration in mice. Eur J Neurosci. 2008;28(4):707-16. Epub 2008/07/29. doi: EJN6371 [pii]

10.1111/j.1460-9568.2008.06371.x [doi]. PubMed PMID: 18657183.

65. Litteljohn D, Nelson E, Bethune C, Hayley S. The effects of paraquat on regional brain neurotransmitter activity, hippocampal BDNF and behavioural function in female mice. Neurosci Lett. 2011;502(3):186-91. PubMed PMID: 21835224.

66. Perry TL, Yong VW, Wall RA, Jones K. Paraquat and two endogenous analogues of the neurotoxic substance N-methyl-4-phenyl-1,2,3,6-tetrahydropyridine do not damage dopaminergic nigrostriatal neurons in the mouse. Neurosci Lett. 1986;69(3):285-9. Epub 1986/09/12. PubMed PMID: 3489912.

67. Shepherd KR, Lee ES, Schmued L, Jiao Y, Ali SF, Oriaku ET, et al. The potentiating effects of 1-methyl-4-phenyl-1,2,3,6-tetrahydropyridine (MPTP) on paraquat-induced neurochemical and behavioral changes in mice. Pharmacol Biochem Behav. 2006;83(3):349-59. PubMed PMID: 16580056.

68. Songin M, Strosznajder JB, Fital M, Kuter K, Kolasiewicz W, Nowak P, et al. Glycogen synthase kinase 3beta and its phosphorylated form (Y216) in the paraquat-induced model of parkinsonism. Neurotox Res. 2011;19(1):162-71. Epub 2010/02/10. doi: 10.1007/s12640-010-9153-7. PubMed PMID: 20143200.

69. Woolley DE, Gietzen DW, Gee SJ, Magdalou J, Hammock BD. Does paraquat (PQ) mimic MPP+ toxicity? Proc West Pharmacol Soc. 1989;32:191-3. Epub 1989/01/01. PubMed PMID: 2789384.
