# Supplementary material for: Assessment of the Effects of MPTP and Paraquat on Dopaminergic Neurons and Microglia in the Substantia Nigra Pars Compacta of C57BL/6 Mice
Source: PLoS One. 2016 Oct 27;11(10):e0164094. doi: 10.1371/journal.pone.0164094 (PMC5082881; doi:10.1371/journal.pone.0164094)
Supplement: S4 Appendix — (DOCX) [file pone.0164094.s004.docx]

**S4 Appendix:** **Stereological and neuropathological studies on paraquat in mice^1^**

| **Reference** | **Strain, Source. Age, Gender, Husbandry** | **Paraquat Source, Dose, Route, Frequency, Duration** | **Neurotoxicity Parameters Evaluated** | **Results** |
| --- | --- | --- | --- | --- |
| Smeyne (2016)  (Current Study)  **Study** **#1**  **(G1 to G5)** | **Strain:** C57BL/6 mice  **Source**: Jackson Labs (ME) and Harlan (IN)  **Gender:** Male  **Age:** 9 or 16 weeks  **Quarantine Period:**  WIL: 2 weeks  SJCRH: 4-6 weeks  **Housing**  **WIL:** Wire-mesh cage; 1 mouse/cage (G1)  **SJCRH**: Plastic shoe box cages; 4 mice/ cage (G2 to G5)  **Number of PQ Mice:** 10-20 mice/group | **PQ dichloride hydrate**  **Sources:** Sigma (SJCRH) or Syngenta (WIL)  **PQ Dose (WIL):** 20 mg/kg (i.p.),1x per week for 3 weeks; Sacrificed 1 week post-dosing for stereology and 8, 16, 24, 48, 96 or 168 hours post-dosing for neuropathology.  **PQ Dose (SJCRH):** 10 mg/kg i.p.. 2x per week for 3 weeks.  Sacrificed 1 week post-dosing.  **Negative Control:** Saline  **Positive Control:** 20 mg/kg MPTP 4x in 1 day.  Sacrificed 7 days post dosing.  **HPLC confirmation of dosing solutions:** Yes | **Stereology**: **TH^+^ Neurons**  **3D Stereology:** 40 µm nominal section thickness (1 in 3 sections evaluated bilaterally) through the SNpc for immune-reactive, DAB chromogen-labeled TH^+^ neurons. Each section examined (MicroBrightfield) through its depth excluding 2 µm guard zones at the top and bottom of the section.  **2D Stereology:** 10 µm sections (1 in 5) through the SNpc for immune-reactive, DAB chromogen-labeled TH^+^ neurons.  **Stereology**: **Iba-1 labeled Microglia**  2D stereology on 10 µm sections (1 in 5) through the SNpc for immune-reactive, Iba-1-labeled resting or activated microglia in the SNpc.  **Neuropathology**  Semi-quantitative neuropathological assessment of SNpc:  • Cell Death (AmCuAg),TUNEL,  Caspase 3)  • TH^+^ neurons: Staining intensity  • Microglia (Iba-1)  • Astrocytes (GFAP)  **Stereology and neuropathology conducted blinded to dose group?** Yes | **Stereology**: **TH^+^ Neurons**  PQ had no effect on the number of TH^+^ neurons in the SNpc based on 2D or 3D stereology.  **Stereology**: **Nissl-Stained Neurons**  PQ had no effect on the number of TH^-^ neurons in the SNpc based on 3D stereology.  **Stereology**: **Iba-1 labeled Microglia**  PQ had no effect on the number of resting or activated microglia in the SNpc based on 2D stereology.  **Neuropathology**  PQ had no effect on TH^+^ staining intensity, DA neuronal cell death or on microglia or astrocytes based upon a blinded, semi-quantitative neuropathological evaluation.  **MPTP Positive Control**  MPTP-treated mice displayed statistically significant positive effects on all stereological and neuropathological endpoints evaluated. |
| Barlow (2004)  [1]  **Study #2** | **Strain:** C57BL/6 mice  **Source**: Jackson Laboratory  **Gender:** Male and female  **Age at receipt:** 6 weeks  **Quarantine Period:** 1 week  **Housing:**  **During Mating**: 1 male mated with 3 females  **During Pregnancy:** Dam and litter housed together  **Post-Weaning**  1 mouse /cage; cage type not given.    **Number of PQ Mice**:  **Dams/PQ group**:24-30 dams  **Pups/PQ group:** 4-5; no more than 2 pups from each litter | **PQ dichloride hydrate**  **Source:** Sigma  **PQ Dose of Dams:** 0.3 mg/kg s.c.; Daily GD 10-17.    **PQ Dose of F1 generation:** 5 mg/kg s.c. Daily PND 48-55  Sacrifice 1 week post-dosing.  **Negative Control:** Saline  **HPLC confirmation of dosing solutions**: Yes | **Stereology: TH^+^ Neurons:**  30 µm nominal section (1 in 8 section evaluated unilaterally) through the SNpc and VTA counted for immune-reactive, DAB chromogen-labeled TH^+^ neurons  **Stereology**: **Nissl-Stained Neurons**  The sections described above were counterstained with cresyl violet and Nissl-stained, TH^-^ neurons were counted.  **Stereology conducted blinded to dose group?** Yes | **Stereology**: **TH^+^ Neurons**  PQ had no effect on TH^+^ or TH^-^ neurons in the SNpc or VTA, irrespective of whether males or females were exposed to PQ during gestation or during gestation and postnatally.  **Stereology**: **Nissl-Stained Neurons**  PQ had no effect on TH^-^ neurons in the SNpc or VTA, irrespective of whether males or females were exposed to PQ during gestation or during gestation and postnatally. |
| Breckenridge (2013) [2]  **Study #3** | **Strain:** C57BL/6 mice  **Source**: Jackson Labs (ME)  **Gender:** Male  **Age at receipt:** 6-7 weeks  **Quarantine Period:** 3 to 5 weeks  **Housing:** Individual stainless-steel, wire-mesh-floored cages    **Number of PQ Mice**:  **Study** **3:** 5 mice for neuropathology  **Study 4:** 5 mice for neuropathology and 10 mice for stereology  **Study 5:** 10 mice for stereology (DAB, DAPI, Alexa red)  **Study 6:** 5 mice for neuropathology at each time point | **PQ dichloride hydrate**  **Source:** Syngenta (Studies 3-5) and Sigma (Study 4)  **PQ Dose:**  **Study 3:** 10 mg/kg i.p., 1x per week for 1 or 2 weeks  **Sacrifice:** 1, 2, 4, or 7 days post-dosing  **Study 4:** 10 mg/kg i.p., 1x per week for 3 weeks: Syngenta and Sigma PQ  **Sacrifice:** 1, 2, 4 or 7 days post-dosing for pathology, 7 day (stereology); or 15 mg/kg i.p., 1x per week for 1, 2 or 3 weeks for Syngenta PQ  **Sacrifice:** 2 or 7 days post-dosing for pathology, 7 days (stereology)  **Study 5:** 10, 15 or 25 mg/kg i.p., 1x per week for 3 weeks  **Sacrifice:** 1 week post-dosing  **Study 6:** 10, 15 or 25 mg/kg i.p., 1x per week for 1, 2 or 3 weeks  **Sacrifice:** 4, 8, 16 hours, 1, 2, 4 or 7 days    **Negative Control:** Saline  **Positive Control:** MPTP  **Analytical confirmation of dosing solutions**: Yes | **Stereology**: **TH^+^/TH^-^ Neurons**  **(Study 4):**  40 µm nominal section thickness (1 in 3 sections evaluated bilaterally) through the SNpc for immune-reactive, DAB chromogen-labeled TH^+^ neurons, counter-stained with cresyl violet. Each section examined through its depth excluding guard zones.  **Stereology (Study 5):** 30 µm sections through the SNpc for immuno-reactive, Alexa red labeled TH^+^ neurons and DAPI-stained TH^-^ neurons  **Neuropathology (Study 3, 4 and 6)**  Semi-quantitative neuropathological assessment of SNpc:  • Cell Death: (AmCuAg,TUNEL,  Caspase 3)  • TH^+^ Neurons: Staining intensity  • Microglia: (Iba-1)  • Astrocytes: (GFAP)  • Nissl staining: (thionine)  **Stereology conducted blinded to dose group?** Yes | **Stereology**: **TH^+^/TH^-^ Neurons**  PQ reduced TH^+^ neurons in the SNpc for the 3x 15 mg/kg dose regime in experiment **4** only. This was not repeated in experiment **5** at 3x 15 or 3x 25 mg/kg dose.  **Neuropathology**:  There was no effect of PQ on indicators of cell death (AmCuAg, TUNEL, Caspase 3), cell loss (TH or Nissl staining) or microglial (Iba-1) or astrocyte (GFAP) activation after 1, 2 or 3 doses of PQ when assessed after multiple time points (4, 8, 16 hrs or 1, 2, 4 or 7 days) after the last dose  **MPTP Positive Control**  MPTP-treated mice displayed statistically significant effects on stereological and neuropathological endpoints. |
| Brooks (1999) [3]  **Study #4** | **Strain**: C57BL/6J mice  **Source**: Jackson Labs  **Gender:** Male  **Age at receipt:** Adult (age not specified)  **Quarantine Period:** Not provided  **Housing:** Not stated    **Number of PQ Mice**: Minimum of 3 mice/group | **PQ dichloride hydrate**  **Source:** Sigma Chemical/RBI  **PQ Dose:** 5 or 10 mg/kg i.p.; once/week for 3 weeks  **Negative Control:** Saline  **Positive Control:** MPTP  A cumulative MPTP dose of 40 or 120 mg /kg administered over two 16-hour periods that were separated by 7 days.  100 nL of a 2% Fluoro-Gold^®^ solution was injected stereotaxically into the striatum in halothane- anaesthetized mice 7 days before the 1^st^ PQ or MPTP dose.  **Sacrifice:** Approximately 12 days after the last dose of PQ or MPTP, brain were collected and processed for image analysis  **Analytical confirmation of dosing solutions:** Not stated | **Tissue Collection and Processing**  Approximately 12 days after the last dose of PQ or MPTP, brains were paraffin-embedded and sectioned (6 μm) through the SNpc. Heat-deparaffinized sections were treated with a primary antibody in order to immunolabel TH^+^ terminals (DAB chromogen) in the striatum and a secondary antibody to visualize Fluoro-Gold^®^-labeled neurons in the SNpc.  Number of sections evaluated/group: 7 to 10 sections/group in a minimum of 3 mice. (9 sectors comprising 15% of the total area of the SNpc were evaluated separately).  **Image Analysis: TH^+^ densitometry**  Image-Pro Plus software to characterize the density of TH^+^ staining in the striatum.  **Image Analysis: Fluoro-Gold**^®^ **florescence**: Assessed in the SNpc  **Image analysis conducted blinded to dose group?** Not stated | **Image Analysis: TH^+^ terminals in the striatum**  Statistically significant reductions were observed in TH^+^ density in the striatum of the 5 (87%) and 10 (94%) mg/kg PQ-treated groups.    In the MPTP-treated groups there were a 76% (40 mg/kg cumulative dose) and 98% (120 mg/kg/day cumulative dose), statistically significant reductions in TH^+^ density in the striatum.  **Image Analysis: Fluoro-Gold**^®^**-labeled neurons in the SNpc**  The percent of Fluoro-Gold^®^-labeled TH^+^ neurons in the SNpc was reduced by 36% in the 5 mg/kg PQ-treated group and by 61% in the 10 mg/kg PQ-treated group.  The percent of Fluoro-Gold^®^-labeled TH^+^ neurons in the SNpc was reduced by 50% and 75% respectively in the 40 and 120 mg/kg cumulative MPTP dose groups. |
| Chen (2008) [4]  **Study #5** | **Strain:** C57BL/6 mice  **Source**: SIPPR-BK Labs, Shanghai  **Gender:** Male  **Age at receipt:** 8 weeks  **Quarantine Period:** Not provided    **Housing:** Not stated  **Number of PQ Mice**: 5 mice/group. | **PQ dichloride hydrate**  **Source:** Sigma Chemical  **PQ Dose**: 7 mg/kg s.c. every 2 days for a total of 10 doses  **Negative Control:** Saline  **Sacrifice:** 8 days post-dosing  **Analytical Confirmation of Dosing Solutions:** Not stated | **Tissue Collection and Processing**  8 days after the last PQ dose, brains were paraffin-embedded and sectioned (5 μm) through the SNpc and striatum. The deparaffinized sections were treated with a primary antibody in order to immunolabel TH^+^ neurons (DAB chromogen) in the SNpc.  **Image Analysis of TH^+^ Neurons**  Kato et al. (2003) [5] was cited as the source of the method used for counting TH^+^ neurons. However, no information was provided on the method used of counting TH^+^ neurons in the paper by Kato et al. (2003) [5].  **Image analysis conducted blinded to dose group?** Not stated | **Image Analysis of TH^+^ Neurons**  A statistically significant (~34%) reduction in the number of TH^+^ neurons was reported for the PQ treated group compared to the control group (Fig. 4)  Data was not included in the quantitative analysis of TH^+^ neurons. |
| Choi (2006a) [6]  **Study #6** | **Strain:** C57BL/6 mice  **Source**: Unknown  **Gender:** Male  **Age at receipt:** 8 weeks  **Quarantine Period:** Not provided  **Housing:** Not stated  **Number of PQ Mice**: 10 mice/group | **PQ dichloride hydrate**  **Source:** Sigma Chemical  **PQ Dose:** 10mg/kg i.p., single dose (there is uncertainty as to whether Choi et al. (2006a) [6] administered PQ as a single dose or whether PQ was given once weekly for 3 week).  **Negative Control:** Saline  **Sacrifice:** 1 week post-dosing  **Analytical Confirmation of Dosing Solutions:** Not stated | **Tissue Collection and Processing**  7 days after the PQ dose, brains were frozen and sectioned (thickness not provided) through the SNpc. The sections were treated with a primary antibody in order to immunolabel TH^+^ neurons (DAB chromogen) in the SNpc.  Nissl-positive neurons were stained with cresyl violet (details not provided)  **Stereology: TH^+^ Neurons**  Methods not provided. Choi (2006a) [6] cited the methods in Manning-Bog et al. (2002, 2003) [7, 8]although Manning-Bog et al. (2002) [7] did not evaluate the number of TH^+^ neurons in the SNpc.  **Was stereology conducted blinded to dose group?** Not stated  **Image Analysis of Nissl-Positive Neurons**  The number of Nissl-positive neurons was counted by computer (optical fractionator) for ~70 sections through the SNpc; cell were counted in the “mid-point region” of the SNpc. Criteria for selecting cells to be counted (i.e. size, optical density) was not provided.  **Image analysis conducted blinded to dose group?** Not stated although it is stated that image analysis was done using image analysis software. | **Stereology: TH^+^ Neurons**  The number of TH^+^ neurons in the SNpc was statistically significantly reduced by approximately 68% in the PQ-treated group compared to controls (Fig. 9)  **Image Analysis of Nissl-Positive Neurons**  The relative density, stated as a percent of control, of Nissl-positive neurons in the SNpc was reduced by ~ 64% in PQ-treated wild-type mice. Statistical significance of results not provided (Fig. 9).    **Overall Assessment:** The ambiguity of the description of the study conduct provided in the methods and results presented in this publication limits the utility of this study. |
| Choi (2006b) [9]  **Study #7** | **Strain:** C57BL/6 mice  **Source**: Unknown  **Gender:** Male  **Age:** 8 weeks  **Quarantine Period:** Not provided  **Housing:** Not stated  **Number of PQ Mice**: 10 mice/group | **PQ dichloride hydrate**  **Source:** Sigma Chemical  **PQ Dose**: Discrepancy noted in statements concerning the number of doses of PQ administered: the Method section (p 255) states that a single PQ dose of 10mg/kg i.p. was administered (the text is identical to that published by Choi et al. (2006a) [6]).  The Results section (p 258) states that PQ (10 mg/kg i.p.) was administered weekly for 3 weeks.  **Negative Control:** Saline  **Sacrifice:** 1 week post-dosing  **Analytical Confirmation of Dosing Solutions:** Not stated | **Tissue Collection and Processing**  7 days after the PQ dose, brains were frozen and sectioned (thickness not provided) through the SNpc. The sections were treated with a primary antibody in order to immunolabel TH^+^ neurons (DAB chromogen) in the SNpc.  Nissl-positive neurons were stained with cresyl violet (details were not provided).  **Stereology: TH^+^ Neurons**  Stereology data were not reported in this study.  **Image Analysis of Nissl-Positive Neurons**  The number of Nissl-positive neurons was counted by computer for ~70 sections/mouse through the SNpc. Cell counts were taken at the “mid-point region of the SNpc.” The criteria used for selecting cells to be counted (i.e. size, optical density) were not provided.  The section describing image analysis in Choi et al. (2006b) [9] is identical to the section appearing in Choi et al. (2006a) [6].  **Image analysis conducted blinded to dose group?** Not stated, although image analysis was done using image analysis software. | **Image Analysis of Nissl-Positive Neurons**  The number of Nissl-positive neurons counted in the SNpc was statistically significantly reduced by approximately 40% in the PQ-treated group compared to controls (Fig. 9).  **Overall Assessment:** The ambiguity of the description of the methods and results presented in Choi et al. (2006b) [9]and the apparent overlap with Choi et al. (2006a) [6]limit the utility of these studies. |
| Choi (2010) [10]  **Study #8** | **Strain:**C57BL/6 Jnk3^+/+^ and Jn3^-/-^ mice  **Source**: Unknown  **Gender:** Male and female  **Age at receipt:** 10 weeks  **Quarantine Period:** 1 week  **Housing:** 4-5 mice per cage  **Number of PQ Mice**: 4 mice/group | **PQ dichloride hydrate**  **Source:** Sigma Chemical  **PQ Dose:** 10 mg/kg i.p. twice weekly for 6 weeks.  **Negative Control:** Saline  **Sacrifice:** 4 days post-dosing  **Analytical Confirmation of Dosing Solutions:** Not stated | **Tissue Collection and Processing**  4 days after the PQ dose, brains were frozen and 40 μm sections through the SNpc were collected. The sections were treated with a primary antibody in order to immunolabeled TH^+^, DAB- chromogen-stained neurons in the SNpc.  Nissl-positive neurons were stained with cresyl violet (details not provided)  **3D Stereology: TH^+^ Neurons:**  40 µm nominal section thickness (1 in 4 sections evaluated bilaterally) through the SNpc for immune-reactive, DAB labeled TH^+^ neurons in 4 PQ-treated mice/group. Each section was examined using the Stereology Module, Slidebook Olympus. Guard zones, counting frame and disector height were not described. An optical fractionator was used to calculate the total number of TH^+^ neurons in the SNpc.  TH^+^ cell counts were assessed by “non-stereological” procedures (methods not provided) in 2 mice. (Data, which was stated to be comparable to stereological assessment, was not provided).  **Image analysis conducted blinded to dose group?** Yes | **Stereology: TH^+^ Neurons**  The percent of TH^+^ neurons in the SNpc was statistically significantly reduced (~ 38%) in PQ-treated C57BL/6 Jnk3^+/+^ mice. There was no effect of PQ on the number of TH^+^ neurons in Jnk3^-/-^ mice.  The authors did not indicate whether the results were for male, female or both sexes pooled although it was stated that the results from behavioral studies collected on the same animals were pooled. |
| Cristovao (2009) [11]  **Study #9** | **Strain:** C57BL/6 mice  **Source**: Charles River (Wilmington, MA)  **Gender:** Male  **Age:** 8-10 weeks  **Quarantine Period:** Not provided  **Housing:** Not stated  **Number of PQ Mice**:  **TH^+^ Neurons:** 7 mice  **Microglia:** Not stated | **PQ dichloride hydrate**  **Source:** Sigma Chemical  **PQ Dose:** 10 mg/kg i.p. on Days 1, 4 and 7  **Negative Control:** 1% DMSO in saline  **Sacrifice:** **Stereology Subset:** 5 days after the 3rd injection  **Microglia Subset:** Evaluated 2, 5 and 12 days after a single dose.  **Analytical Confirmation of Dosing Solutions:** Not stated | **3D Stereology: TH^+^ Neurons**  40 µm nominal section thickness (1 in 4 sections evaluated bilaterally) through the SNpc for immune-reactive, DAB- labeled TH^+^ neurons and counter-stained with cresyl violet.  Each section was examined using MicroBrightfield Stereo Investigator (v 4.35)  Counting frame thickness = 14 µm; Sample grid 140 µm;  Counting frame area = 4900 µm^2^  Number of sections evaluated: Not stated.  **Statistical Analysis**: 1 way ANOVA followed by the Dunnett test or Bonferroni multiple comparisons.  **Microglia Assessment:** For the microglia subset of mice, sections through the SNpc (number evaluated unspecified) were immunostained antibody to CD11b.  **Stereology and microglial evaluation conducted blinded to dose group?** Not stated | **Stereology: TH^+^/Cresyl Violet Neurons**  A statistically significant (~35%) reduction in the number of TH^+^ neurons was observed, presumably 5 days after the 3^rd^ PQ dose.  A statistically significant (~25.3%) reduction in the number of cresyl violet- labeled cells was observed 5 days after the 3^rd^ PQ dose (Figure 8b, 8c).  **Microglia**  Reported that PQ mice displayed a morphological change in microglia (enlarged cell bodies, loss in processes) compared to control 1 day after the 1^st^ dose (quantitative data not provided).  Observable CD-11b – positive microglia declined by Day 4 and they were undetected by Day 12 (quantitative data were not provided). |
| Fei (2008) [12]  **Study #10** | **Wild Type Strain:** C57BL/6J  **Source:** Charles River (Hollister, CA)  **Gender:** Male  **Age:** 8 weeks  **Quarantine Period:** Not provided  **Housing:** Not stated  **Number PQ Mice:** 3 mice/group  **SBak-/-**  **Strain:** B6-129-Bak1-/-/J  **Source:** Jackson Labs  **Gender:** Male  **Age:** 8 weeks  **Quarantine Period:** Not provided    **Housing:** Not stated  **Number of PQ Mice**: 3 mice/group | **PQ dichloride hydrate**  **Source:** Sigma Chemical  **PQ Dose:** 10 mg/kg i.p. doses once/week for 3 weeks  **Negative Control**: Saline  **Neuroprotection:** Bak^-/-^mice  **Wild Type Sacrifice:** 2 days after the 2^nd^ dose  **Bak -/- Sacrifice:** 1 week after the 3^rd^ dose  **Analytical Confirmation of Dosing Solutions:** Not stated | **Stereology: TH^+^ Neurons and Total Neurons (cresyl violet stained)**  **3D Stereology:** 40 µm nominal section thickness (1 in 6 sections evaluated unilaterally; total of 3 sections/mouse brain) through the SNpc for immuno-reactive DAB-labeled TH^+^ neurons and counter-stained with cresyl violet.  Each section was examined using MicroBrightfield Stereo Investigator.    **Stereology conducted blinded to dose group?** Yes | **Wild Type C57BL/6J Mice** A statistically significant (~31%) reduction in the number of TH^+^ neurons in the SNpc was found in mice administered 10 mg/kg PQ, once weekly for 3 weeks.  The total number of cresyl violet-stained neurons in the SNpc was reduced ~28%.    **C57BL/6J (Bak^-/-^) Mice** No statistically significant differences were observed in the mean number of TH^+^ neurons or the mean number of total neurons in the SNpc of Bak-/- PQ-treated mice compared to the vehicle control group. |
| Fernagut (2007) [13]  **Study #11** | **Wild Type**  **Strain:** C57BL/6-DBA/2  **Source:** UCLA colony  **Age:** 12-14 weeks  **Number of Litters:** 7  **Housing:** Not stated  **Number PQ Mice**: 7 mice/group  **Human α-synuclein** over-expression under the Thy-1 promoter  **Strain**: On wild-type background.  **Age:** 12-14 weeks  **Quarantine Period:** Not provided  **Housing:** Not stated  **Number of mice:** 7 mice/group  **Number of PQ Mice:** 7 mice/group | **PQ dichloride hydrate**  **Source:** Sigma Chemical  **PQ Dose:** 10 mg/kg i.p. doses once/week for 3 weeks  **Negative Control:** Saline  **Positive Control:** α-synuclein over-expression  **Sacrifice:** 5 days after the 3^rd^ dose  **Analytical Confirmation of Dosing Solutions:** Not stated | **TH^+^ Neurons in the SNpc**  40 µm nominal section thickness (1 in 4 sections evaluated unilaterally through the SNpc for immune-reactive DAB- labeled TH^+^ neurons. Each section was examined using MicroBrightfield Stereo Investigator (Guard Z1: 1.5 µm).  Sub-regions of the SNpc were evaluated:   1. 250 µm medial portion near the VTA 2. A lateral portion near the external tip of the cerebral peduncle 3. Central region between the medial and lateral regions. 4. Total SNpc   CD-68 immune-reactive micronuclei were counted at the mid-nigral level (Bregma 3.16 mm).    **Densitometric Image Analysis in Striatum:** Optical density of TH^+^ stained fibers (Bregma ^+^ 0.98) was assessed in 75 µm^2^ areas of the dorsolateral, dorso-medial, ventolateral and ventromedial portions of the striatum in both the right and left hemispheres.  **Reference**: Corpus callosum  **Optical density of TH^+^ Immune-reactivity in the striatum.**  **Reference:** Cortex  **Stereology conducted blinded to dose group?** Yes | **TH^+^ Neurons in the SNpc**  Statistically significant reductions in the number of TH^+^ neurons in the SNpc found in wild type (26.7%) and α-synuclein over-expressed (25.6%) mice administered 10 mg/kg PQ, once weekly for 3 weeks.  Of the 3 sub-regions evaluated, the ventral portion of the SNpc was most affected by PQ treatment (30.4 to 37.6% reduction).  **Optical Density Studies in Striatum**  Non-statistically significant reductions noted in TH^+^ optical density (WT: 12.7%; synuclein overexpressed 15.8%).  TH^+^ fiber density was significantly reduced in PQ-treated mice, mainly in the dorsomedial striatum of the WT (-18.5%) and synuclein over-expressed (-25%).  **Microglia Study in Mid-Nigral Region**  There was no effect of PQ treatment on the number of CD68 positive microglia (data not shown). |
| Gollamudi  (2012) [14]  **Study #12** | **Strain:** C57BL/6J mice  **Source**: Jackson Labs  **Gender:** Male  **Age:** 3 months  **Quarantine Period:** Not provided  **Housing:** Not stated  **Number of PQ Mice**: 6 mice/group | **PQ dichloride hydrate**  **Source:** Sigma Chemical  **PQ Dose:** 10 mg/kg/day; continuous subcutaneous injection by Alzet pump for 7 days  **Negative Control:** PBS as a 1:8 mix with ethanol and neobee^©^ M-5 oil  **Sacrifice:** At the end of Day 7  **Analytical Confirmation of Dosing Solutions:** Not stated | **3D Stereology: TH^+^ Neurons**  50 µm nominal section thickness (7 sections were evaluated bilaterally) through the SNpc for immune-reactive, DAB-labeled TH^+^ neurons. Each section was examined using MicroBrightfield Stereo Investigator (V 9.12).  **Stereology conducted blinded to dose group?** Not stated | **TH^+^ Neurons in the SNpc**  A statistically significant (35%) reduction in the number of TH^+^ neurons in the SNpc was found in mice administered 10 mg/kg/day PQ, administered for 7 days as a continuous subcutaneous injection.  The absolute number of TH^+^ neurons in the SNpc of control or PQ-treated mice was not reported. |
| Jiao (2012) [15]  **Study #13** | **Strains:**  C57BL/6J mice  SWR/J mice  **Source**: Jackson Labs  **Gender:** Male  **Age:** 9 weeks  **Quarantine Period:** 2 to 3 weeks  **Housing:** 5 mice/cage in plastic shoe-box cages  **Number of PQ mice:** 6 mice/group | **PQ dichloride hydrate**  **Source:** Sigma Chemical  **PQ Dose:** 10 mg/kg i.p. injection twice weekly for 3 weeks  **Negative Control:** Saline  **Sacrifice:** 7 days after the last dose  **Analytical Confirmation of Dosing Solutions:** No | **2D Stereology: TH^+^ Neurons**  10 µm sections (1 in every 6 sections) were evaluated bilaterally) through the SNpc for immune-reactive, DAB-labeled TH^+^ neurons. Stereology was conducted using MicroBrightfield Stereo Investigator (2D stereological method).  **Stereology conducted blinded to dose group?** No | **TH^+^ Neurons in the SNpc: C57BL/6J mice**  A statistically significant (~47%) reduction in the number of TH^+^ neurons in the SNpc was found in mice administered 10 mg/kg/day PQ, twice weekly for 3 weeks.  **TH^+^ Neurons in the SNpc: SWR/J mice**  There was no statistically significant effect of PQ on TH^+^ neurons in the SNpc of SWR/J mice administered 10 mg/kg/day PQ, twice weekly for 3 weeks. |
| Kang (2009) [16]  **Study #14** | **Strain:** C57BL/6 mice  **Source**: Japan SLC  **Gender:** Male  **Age:** 7 weeks  **Quarantine Period:** 1 week  **Housing:** Not stated  **Number of PQ mice:** 5 mice/group | **PQ dichloride hydrate**  **Source:** Sigma-Aldrich  **PQ Dose:** 10 mg/kg i.p. injection twice weekly for 3 weeks  **Negative Control:** Saline  **Sacrifice:** 7 days after the last dose  **Analytical Confirmation of Dosing Solutions:** Not stated | **Computer Image Analysis: Number of TH^+^ Neurons in the SNpc**  40 µm nominal section thickness through the SNpc for immune-reactive, DAB labeled TH^+^ neurons.  1 section every 300 µm was evaluated Unstated if sections were evaluated bilaterally but the TH^+^ neuron counts reported are consistent with bilateral examination).  Stereological methods: not provided other than stating that images were analyzed for TH immune-reactive neurons under bright-field illumination; only TH^+^ neurons with nuclei were counted.  **Computer Image Analysis: TH^+^ Fiber Density in the SNpr**  The optical density of the region evaluated in PQ-treated mice was compared to the optical density of a comparable section from untreated controls.  **Image analysis conducted blinded to dose group?** Not stated | **TH^+^ Neurons in the SNpc**  A statistically significant (~39%) reduction in the number of TH^+^ neurons in the SNpc was found in mice administered 10 mg/kg/day PQ, twice weekly for 3 weeks.  The absolute number of TH^+^ neurons in the SNpc of control or PQ treated mice was not reported.  **Optical Density in the SNpr**  A statistically significant (~37%) reduction in optical density (attributed to TH^+^ fibers in the SNpr) was found in mice administered 10 mg/kg/day PQ, twice weekly for 3 weeks when compared to controls.  **Correlated Striatal Neurochemistry**  Kang et al. (2009) [17] reported a statistically significant decrease in striatal DA and an increase in DA turnover in PQ-treated mice. |
| Kang (2010) [18]  **Study #15** | **Strain:** C57BL/6 mice  **Source**: Japan SLC  **Gender:** Male  **Age:** 7 weeks  **Quarantine Period:** 1 week  **Housing:** Not stated  **Number of PQ mice:** 5 mice/group | **PQ dichloride hydrate**  **Source:** Sigma-Aldrich  **PQ Dose:** 10 mg/kg i.p. injection twice weekly for 3 weeks  **Negative Control:** Saline  **Sacrifice:** 7 days after the last dose  **Analytical Confirmation of Dosing Solutions:** Not stated | **Computer Image Analysis: TH^+^ Fiber Density in the Rostral and Caudal Striatum**  A 40 µm coronal section through rostral (Bregma = ^+^1.18) and caudal (Bregma = -0.46 mm were stained for immune-reactive, DAB-labeled, TH^+^ neuronal fibers.    Mean optical density (OD) in each of the rostral and caudal striata was calculated by averaging the OD for the dorsal and ventral portions of each section after subtracting the OD measured in the corpus callosum.  Mean OD for the dorsal and ventral portions of each of the rostral and caudal portions of the striatum were also calculated  The OD in PQ treated mice was expressed as a percentage of the control group OD evaluated in the same regions of the striatum  **Image analysis conducted blinded to dose group?** Not stated | **Optical Density in the Rostral Striatum**  A statistically significant (~13%) reduction in OD (attributed to TH^+^ fibers in the dorsal portion of the striatum; Fig. 3C) was found in mice administered 10 mg/kg/day PQ, twice weekly for 3 weeks compared to controls. A similar magnitude reduction in the ventral striatum (Fig. 3C) was not statistically significant.  **Optical Density in the Caudal Striatum**  A statistically significant (~18%) reduction in OD (attributed to TH^+^ fibers in the dorsal and ventral portions of the caudal striatum; Fig. 4C) was found in mice administered 10 mg/kg/day PQ, twice weekly for 3 weeks compared to controls.  **Correlated Striatal Neurochemistry**  In contrast to the majority of studies, but consistent with Kang (2009) [17], Kang (2010) [18] reported statistically significant decreased striatal DA in all sub-regions of the striatum (Fig 5); DOPAC was only significantly decreased in ventral region of the caudal striatum. |
| Khawaja (2007) [19]  **Study #16** | **Strain:** C57BL/6 mice  **Source**: Charles River (Gilroy, CA)  **Gender:** Male  **Age:** 8-10 months old  **Quarantine Period:** 2 days  **Housing:** 1 or 2 mice per cage  **Number of PQ Mice**: 3-6 mice/group | **PQ dichloride hydrate**  **Saccharin: 2%**  **Nicotine: free base**  **Source:** Sigma-Aldrich  **PQ Dose**: 10 mg/kg i.p. injection once weekly for 3 weeks commencing on week 3 (Day 21)  **Nicotine ^+^ Saccharin doses:** in drinking water (Fig. 1)  **Nicotine Concentration**:  25 µg/ml (Day 2)  50 µg/ml (Day 3-6)  100 µg/ml (Day 7-8)  200 µg/ml (Day 9-10)  300 µg/ml (Day 11-13)  400 µg/ml (Day 14-42)  0 µg/ml (Day 29)    **Negative Control:** Saline  **Positive Control:** N1  **Sacrifice:** 7 days after the last PQ dose, nicotine was withdrawn 18 hours before mice were killed by cervical dislocation.  **Analytical Confirmation of Dosing Solutions:** Not stated | **3D Stereology: TH^+^ Neurons and Total Neurons (cresyl violet stained)**  40 µm thick sections (1 in every 6th section was evaluated throughout the SNpc were stained for immune-reactive DAB- labeled TH^+^ neurons and counter-stained with cresyl violet. Each section was examined using MicroBrightfield Stereo Investigator.  It was not stated whether the stereological evaluation was conducted unilaterally or bilaterally, although counts reported are consistent with a unilateral evaluation).    **Stereology conducted blinded to dose group?** Yes | **TH^+^ Neurons in the SNpc (Fig. 2)**  A statistically significant (~25%) reduction in the number of TH^+^ neurons in the SNpc was found in mice administered 10 mg/kg/day PQ, twice weekly for 3 weeks.  PQ-treated mice that received nicotine in drinking water also sustained a significant reduction (-16%) in the number of TH^+^ neurons, although the magnitude of the reduction was less than observed in mice that did not receive nicotine.  **Nissl-stained Neurons in the SNpc**  A statistically significant (~25%) reduction in the number of Nissl-stained neurons in the SNpc was found in mice administered 10 mg/kg/day PQ, twice weekly for 3 weeks.  PQ-treated mice that received nicotine in drinking water also sustained a significant reduction (-9%) in the number of TH^+^ neurons, although the magnitude of the reduction was less than observed in mice that did not receive nicotine. |
| Li (2005)[20]  **Study #17** | **Strain:** C57BL/6 mice  **Source**: Animal Center of Chinese Medical University (Shenyang, China)  **Gender:** Male  **Age:** 10 months  **Quarantine Period:** Not provided  **Housing:** Not stated  **Number of PQ Mice**:  TH^+^ neurons: 8 in the control group and 9-10 mice/PQ group | **PQ dichloride hydrate**  **Source:** Sigma-Aldrich  **PQ Dose:** 5 or 10 mg/kg i.p. injection every 2 days for a total of 10 doses administered over 20 days.  **Negative Control:** Saline  **Sacrifice:** 8 days after last dose by decapitation. 1 mouse in the 10 mg/kg PQ dose group died.  **Analytical Confirmation of Dosing Solutions:** Not stated | **TH^+^ Neurons in the SNpc**  The method for collecting, sectioning, staining and counting the number of TH^+^ neurons in the SNpc were not provided.  **TH^+^ neurons counted blinded to dose group?** Yes | **TH^+^ Neurons in the SNpc**  The mean number TH^+^ neurons counted in the control group was ~77 (Fig. 2).  The mean number of TH^+^ neurons counted in the SNpc of PQ treated mice was statistically significantly reduced in the 5 mg/kg (~52%) and 10 mg/kg (~57%) PQ treated groups. |
| Li (2012)  [21]  **Study #18** | **Strain:** C57BL/6 mice  **Source**: SLAC Lab. Animal Co. Ltd. (Shanghai, China)  **Gender:** Male  **Age:** 8 weeks  **Quarantine Period:** Not provided  **Housing:** Not stated  **Number of PQ Mice**: 5 mice/group | **PQ dichloride hydrate**  tert-butylhydroquin1 (tBHQ)  **Source:** Sigma-Aldrich  **PQ Dose:** 7 mg/kg i.p. injection every 2 days for a total of 10 doses administered over 20 days.  **Negative Control:** Saline  **Neuroprotection Control**: 1% tBHQ (w/w) in food pellets 3 days prior to the 1^st^ PQ dose  **Sacrifice:** Killed by cardiac perfusion of fixative, 7 days after the last dose.  **Analytical Confirmation of Dosing Solutions:** Not stated | **TH^+^ Neurons in the SNpc**  6 µm thick sections through the SNpc were stained for immune-reactive DAB- labeled TH^+^ neurons. The number of TH^+^ neurons was counted in 3 “standardized” sections from the same region of the SNpc.  **Evaluation of TUNEL-Staining and Apoptotic Neurons in the SNpc**  TUNEL-positive and apoptotic cells were counted in 3 “standardized” sections through the SNpc.  Cellular apoptosis indicated by   1. Marked condensation of chromatin and cytoplasm or the presence of 2. cytoplasmic fragments or 3. micronuclei   Apoptotic Index: Average percentage of TUNEL-positive cells counted in 10 fields in the 3 sections evaluated.  **TH^+^, TUNEL-positive and apoptotic neurons counted blinded to dose group?** Yes | **TH^+^ Neurons in the SNpc (Fig. 1B)**  A statistically significant ~ 46% reduction was observed in the mean number of TH^+^ neurons counted in 3 section through the SNpc of PQ-treated mice compared to controls.  The effect of PQ on TH^+^ neurons in tBHQ pre-treated mice was significantly reduced (~ 23%) but it was less than that observed in non-tBHQ treated mice.  **Apoptotic Index in the SNpc (Fig. 1C)**  The mean apoptotic index was statistically significantly increased (3.2-fold) in PQ treated mice compared to controls.  The effect of PQ on the apoptotic index in tBHQ pre-treated mice was significantly increased (1.6-fold) but it was less than that observed in non-tBHQ treated mice. |
| Mangano (2009) [22]  **Study #19** | **Strain:** C57BL/6 mice  **Source**: Charles River (Laprairie, Quebec).  **Gender:** Male  **Age:** 9-10 weeks  **Quarantine Period:** 2 weeks  **Housing:** Single polypropylene cages (see Mangano et al. (2011) [23])  **Number of PQ Mice**: 10 mice per group | **PQ dichloride hydrate**  **Lipopolysaccharide (LPS)**  **Source:** Not reported  **LPS:** 0.1 or 2 µg injected over 5 minutes via a cannula above the SNpc located at bregma -3.16 a-p; 1.2 mm lateral; -4 mm ventral.  **PQ Dose:** 10 mg/kg PQ i.p. administered 3 times/week for 3 weeks. Dosing began either 2 or 7 days after LPS priming.  **Negative Control:** Saline  **Sacrifice Schedule:** Killed by cardiac perfusion of fixative, 5 days after the last PQ dose.  **Analytical Confirmation of Dosing Solutions:** Not stated | **Microglia, Astrocytes in the Striatum**  14 µm thick sections through the striatum (1 ever 6 sections) were stained for immune-reactive CD11b-labeled microglia and GFAP-labeled astrocytes.  Microglia were classified by 2 raters who were blinded to treatment into the following 3 categories:  Category 0: Resting  Category 1: Intermediate reactive state  Category 2: Majority of cells display intermediate or highly activated shape. Category 3: Large number of cells display activated, amoeboid shape.  **TH^+^ Neurons in the SNpc**  The number of TH^+^ neurons was counted in at least 2 sections/mouse at each of 5 levels through the SNpc from bregma -3.08 to bregma -3.40 mm.  The number of TH^+^, NeuN^+^ neurons were also counted as were NeuN^-^. Fluoro-Jade B used to detect neuro-degeneration in surviving neurons.  **TH^+^ Terminal Density in Striatum**  Average TH^+^ terminal density in the striatum was determined. Methods were not provided in this publication (see Mangano et al. (2011) [23]for the detail)}.  **Analyses conducted blinded to dose group?** Yes | **TH^+^ Neurons in the SNpc (Fig. 4)**  The mean number of TH^+^ neurons was statistically significant (~22- 25%) reduced in the SNpc counted at bregma -3.08 of PQ-treated mice compared to controls. The reduction (42%) was greater when PQ-treated mice were administered LPS 2 days prior to PQ treatment; there was no effect of LPS priming 7 days prior to PQ treatment initiation. The mean number of TH^+^ plus NeurN^+^ neurons in the SNpc was reduced by 40% in day 2, LPS-primed, PQ-treated mice (Fig. 4I). There was no effect of PQ on the number of TH^+^ neurons in the SNpc counted at bregma -3.40.in the PQ-treated group (Mean ~46.1 TH^+^ neurons) vs. the control group (Mean ~ TH^+^ 51.8 neurons). PQ-treated mice primed with LPS 2 days prior to PQ displayed a statistically significant, 52% reduction. There was no effect at bregma 3.5 when LPS priming occurred 7 days prior to PQ treatment.  **Fluoro-Jade B (FJB)**  Cells labeled with FJB were detected at the site of LPS injection and elsewhere in the SNpc of LPS-primed, PQ-treated mice. Quantitative data not provided.  **Microglia/Astrocytes**  CD11b labeled microglia were increased and GFAP labeled astrocytes were decreased in PQ-treated mice. No quantitative data provided. |
| Mangano (2011) [23]  **Study #20** | **Strain:** C57BL/6 mice  **Source**: Charles River (Laprairie, Quebec)  **Gender:** Male  **Age:** 10-12 weeks  **Quarantine Period:** 1 week  **Housing:** Single polypropylene cages  **Number of PQ Mice**: 8-10 mice per group | **PQ dichloride hydrate**  **Lipopolysaccharide (LPS)**  **Source:** Sigma-Aldrich  **PQ Dose:** 10 mg/kg PQ administered by ip injection, 3 times/week for 3 weeks. Dosing began 2 days after LPS priming.  **LPS:** 0.1µg/2µl injected over 5 minutes via a cannula above the SNpc located at bregma -3.16 a-p; 1.2 mm lateral; -4 mm ventral.  **Negative Control:** Saline  **Sacrifice:** Killed by cardiac perfusion of fixative, 5 days after the last PQ dose.  **Analytical Confirmation of Dosing Solutions:** Not stated | **TH^+^ Neurons in the SNpc (Exper. 1)**  20 µm thick sections through the striatum (1 in every 6 sections) were stained for immune-reactive DAB-labeled TH^+^ neurons which were counterstained with cresyl violet. The number of TH^+^ neurons and the number of cresyl violet-stained neurons was counted in at least 2 sections/ mouse at each of 5 levels through the SNpc from bregma -3.08 to bregma -3.40 mm (see Mangano et al. (2009) [22]).  **TH^+^ Neurons in the SNpc (Exper. 2)**  60 µm thick sections (1 in every 2 sections) through the SNpc were DAB-immuno-stained for TH^+^ neurons and counterstained with cresyl violet. The number of TH^+^ neurons was determined stereologically, islateral to the LPS injection using MicroBrightfield’s optical fractionator (Guard zone = 3 µm; Disector height = 15 µm; Counting frame = 60 x 60 µm).  **Microglia (CD11b), GFAP, NeurN** **in the SNpc**: See Mangano et al. (2009) [22].  **TH^+^ Neuronal Processes in Striatum**  Photomicrographs of TH^+^ stained sections through the striatum were image analyzed for the average intensity of dark staining (TH^+^) pixels.  **Analyses conducted blinded to dose group?** Yes | **TH^+^ Neurons in the SNpc (Exper. 1)**  The mean number of TH^+^ (at bregma= -3.08) was significantly (~28%) reduced in PQ-treated mice and ~-46% reduced in LPS primed, PQ-treated mice. Similar results were reported at bregma -3.16, 3.28 and 3.40 (Fig. 1).  **TH^+^ Neurons in the SNpc (Exper. 2)**  The mean number of TH^+^ neurons was reduced by ~25 to 30% evaluated by stereology in PQ treated mice and by ~40% in LPS primed, PQ-treated mice. The number of cresyl violet stained, TH^-^ neurons unaffected by PQ treatment; cresyl violet and NeuN data were not provided.  **TH^+^ Neuronal Processes in Striatum**  A statistically significant, 35% reduction was observed in the intensity of TH^+^ staining in the striatum in PQ-treated mice that did not receive LPS priming (Fig.2) LPS primed mice displayed a similar reduction.  **Microglia (CD11b) in the SNpc**  The presence of CD11b stained microglia was reported to be increased and the presence of GRAP astrocytes decreased in PQ-treated mice compared to controls; quantitative data were not provided.  The categorical rank assigned to microglia activation was increased approximately 4-fold in PQ-treated mice relative to controls (Figure 7C). |
| Mangano (2012) [24]  **Study #21** | **Strain:** C57BL/6 mice or IFN-γ (Interferon–γ) knockout (KO) mice created on a C57BL/6 background  **Source**: Jackson Lab (Bar Harbor, ME)  **Gender:** Male  **Age:** 8-10 weeks  **Quarantine Period:** 2 weeks  **Housing:** Single polypropylene cages  **Number of Mice:**  **Experiment 1:** 10 mice/group | **PQ dichloride hydrate**  **Source:** Sigma-Aldrich  **Experiment 1**  **PQ Dose:** 10 mg/kg PQ i.p. administered 3 times/week for 3 weeks  **Negative Control:** Saline  **Sacrifice:** Killed by decapitation, 7 days after the last PQ dose  **Analytical Confirmation of Dosing Solutions:** Not stated | **TH^+^ Neurons in the SNpc (Exper. 1)**  20 µm thick sections through the striatum (1 in every 6 sections) were stained for immune-reactive DAB-labeled TH^+^ neurons and counterstained with cresyl violet.  The number of TH^+^ neurons was counted in at least 2 sections/ mouse at each of 5 levels through the SNpc from bregma -3.08 to bregma -3.40 mm (see Mangano et al. (2009) [22])  **Microglia (CD11b) in the SNpc**: See Mangano et al. (2009) [22] for methods.  **Analyses conducted blinded to dose group?** Yes | **TH^+^ Neurons in the SNpc (Exper. 1)**  The mean number of TH^+^ (at bregma= -3.08) was significantly (~36%) reduced in PQ-treated wild-type mice.  In in IFN-γ (KO) mice, there was no effect of PQ on the number of TH^+^ neurons. Similar results were obtained at bregma -3.08, 3.16, 3.28 and 3.40 (Fig 1).  The mean number of cresyl violet-stained neurons was also reported reduced in wild –type C57BL/6 mice but not in the IFN-γ (KO) mice (data not provided).  **Microglia (CD11b) in the SNpc**  Quantitative data were not provided, only a “representative photomicrograph”. |
| Manning-Bog (2003) [8]  **Study #22** | **Strain:** Embryos were from a cross between Swiss Webster 3 and C57blk6 /DBAF1 parents. Transgene mice had  1) human α-synuclein over-expressed or  2) α-synuclein mutation (Ala53Thr substitution).  **Source**: Developed by Matsuoka et al. (2001) [25]  **Gender:** Not stated; presumed to be males  **Age:** 3-4 months  **Quarantine Period:** Not provided  **Housing:** Not stated  **Number of Mice**  **Experiment 1:** 5 mice/ group | **PQ dichloride hydrate**  **Source:** Not specified  **Experiment 1**  **PQ Dose:** 10 mg/kg PQ i.p. administered 1 time/week for 3 weeks  **Negative Control:** Saline  **Sacrifice:** Killed by cervical dislocation, 7 days after the last PQ dose.  **Analytical Confirmation of Dosing Solutions:** Not stated | **TH^+^ Neurons in the SNpc**  40 µm thick sections through the striatum (1 in every 6 sections) were stained for immune-reactive DAB-labeled TH^+^ neurons and counterstained with cresyl violet.  An optical fractionator method was used to count TH^+^ and Nissl^+^ neurons. Details not provided (see McCormack et al. (2002) [26])  **Silver Staining in the SNpc**  Silver stain was used to identify degenerating neurons in the SNpc.  Details on the number of sections stained and evaluated were not provided.  **Analyses conducted blinded to dose group?** Not stated | **TH^+^ Neurons in the SNpc (Table 1)**  The mean number of TH^+^ neurons in PQ-treated wild type mice (9,462±296; N =5) was significantly reduced (~22%) compared to saline controls (12,084±143; N=5). The SEM’s were within 1-3% of the mean.  There was no effect of PQ treatment in mice that had an over-expression of  human α-synuclein or α-synuclein mutation (Ala53Thr substitution).  **Nissl^+^ Neurons in the SNpc (Table 1)**  The mean number of Nissl^+^ neurons in PQ-treated wild type mice (13522±493; N=5) was significantly reduced (~18%) compared to saline controls (16484±476; N =5). SEM’s were within 3-4% of the mean.  There was no effect of PQ treatment in mice that had an over-expression of  human α-synuclein or α-synuclein mutation (Ala53Thr substitution).  **Silver Staining in the SNpc (Fig 1)**  A single photomicrograph was presented to illustrate the presence of silver stained neurons in a PQ-treated mouse but not in a control mouse. No quantitative data was presented. |
| McCormack (2002) [26]    **Study #23** | C57BL/6 mice  **Likely Source**: Simonsen Labs as referenced by McCormack et al. (2002) [26]; Chan et al. (1997) [27] and Manning-Bog et al. (2002) [7].  **Gender:** Male  **Age:** 6 or 8 weeks;  5 or 18 months. (Data for 8 week & 6 month old mice were not provided)  **Quarantine Period:** Not provided  **Housing**: Not stated.  **Number of PQ mice**  **Stereology:** Group ≥ 4 mice/group  **Silver Stain:** Data for 1 PQ-treated and 1 control mouse shown.  **Western Blot:** ≥ 4 mice/group | **PQ dichloride hydrate**  **Source:** Sigma Chemical  **PQ Dose - Stereology:** 1, 5 or 10 mg/kg i.p., 1x per week for 3 weeks in 6 week old or 5- and 18-month-old mice.  **PQ Dose - Western Blot**: 10 mg/kg i.p.; 1 time/week for 3 weeks.  **Negative Control:** Saline  **Sacrifice - Stereology:** Mice killed 1 week after the last dose.  **Western Blots**: Mice killed 2 days after the last dose.  **Analytical Confirmation of Dosing Solutions:** Not stated | **3D Stereology: TH^+^ Neurons**  40 µm sections though the SNpc were collected and TH^+^ neurons were labeled with immune-reactive DAB chromogen and counterstained with cresyl violet.  1 of every 6^th^ section was evaluated.  **Stereology conducted blinded to dose group:** Not stated.  **Silver Stain (Degenerating Neurons)**  SNpc sections stained with silver (method not provided) from mice (number not provided) dosed with 10 mg/kg PQ i.p. for 3 weeks (age not provided).  **Western Blot: Microglia & Astrocytes**  (Homogenized ventral mesencephalon) (Not clear whether the SNpc was dissected and analyzed separately from the SNr, frontal cortex & cerebellum).  Western blots were evaluated (% optical density) using antibodies to MAC-1 (microglia) or GFAP (astrocytes) Mice dosed with 10 mg/kg PQ i.p. for 3 weeks (age of mice not stated) and sacrificed 2 days after the last dose.  **Analyses conducted blinded to dose group?** Not stated. | **Stereology**: **TH^+^ Neurons**  10, 18 and 28% decreases in the number of TH^+^ neurons in the SNpc at 1, 5 and 10 mg PQ/kg, respectively.  An ~25% decrease in the number of TH^+^ neurons was observed in mice given PQ at 6 weeks & 5 months. 33% decrease in mice aged 18 months.  There was 31.6% and 24.6% reduction in TH^+^ and Nissl-stained neuron counts respectively, observed in the 10 mg/kg PQ group compared to controls (age of mice not stated - Table 1)  **Silver Stain**  A single photomicrograph was shown at 2 levels of magnification to indicate silver staining in the midbrain of 1 10 mg/kg PQ-treated mouse compared to 1 control. No quantitative data was provided.  **Western Blot: Microglia & Astrocytes**  Statistically significant increases in the optical density for MAC-1 (~40%) and GFAP (~20%) was observed in the ventral mesencephalon |
| McCormack (2003) [28]  **Study #24** | C57BL/6 mice  **Source:** Not provided (see McCormack et al. (2002) [26])  **Gender:** Male    **Age:** 8-9 weeks  **Quarantine Period:** Not provided    **Housing**: Not stated  **Number of PQ mice:** 4 mice/group | **PQ dichloride hydrate**  **Source:** Sigma Chemical  **PQ Dose**:10 mg/kg i.p.,  1x per week for 3 weeks  **Negative Control:** Saline  **Sacrifice:** 1 week after last dose  **Analytical Confirmation of Dosing Solutions:** Not stated | **3D Stereology: TH^+^ Neurons** Stereological methods reported by McCormack et al. (2002) were used to assess DAB chromogen labeling of TH^+^ neurons (counterstained with cresyl violet) in the SNpc.  **Stereology conducted blinded to dose group?** Not stated | **Stereology: TH^+^ Neurons**  A statistically significant reduction (~25.6%) was observed in the number of TH^+^, neurons in the SNpc of the PQ-treated group (9,284 ±354) compared to the saline control (12,476 ±376).  **Stereology: Nissl-Stained Neurons**  A statistically significant reduction (25.8%) was observed in the mean number of Nissl-stained neurons in the SNpc of the PQ-treated mice (12,646 ±780) compared to the saline control group (17,042 ±322; mean ± SEM). |
| McCormack (2005) [29]  **Study #25** | **Strain:** C57BL/6 mice  **Source:** Charles River (Hollister, CA)  **Strain:** Ferritin transgenic mice  **Source:** Kaur et al. (2003) [30]  **Gender:** Male  **Age:** 8-9 weeks  **Quarantine Period:** Not provided  **Housing:** Not stated  **Number of PQ mice:** Minimum of 4 mice/ group | **PQ dichloride**    **Source:** Sigma Chemical  **PQ Dose:**10 mg/kg i.p.,  1x per week for 1, 2 or 3 weeks of dosing  **Negative Control:** Saline  **Sacrifice:** Mice killed 7 days after the 1^st^ dose; 1, 2, 4 or 7 days after the 2^nd^ dose or 2 or 7 days after the 3^rd^ dose  **Analytical Confirmation of Dosing Solutions:** Not stated | **3D Stereology: TH^+^ Neurons**  3D stereology on 40 µm sections. Stereological methods reported by McCormack et al. (2002) [31] were used to assess DAB chromogen-labeled, TH^+^ neurons in the SNpc (counterstained with cresyl violet).  **Image Analysis: 4-HNE-Positive Neurons**  Single section through A9 (SNpc) or A10 (ventral midbrain tegmentum) was immnunostained for 4-hydroxy-2-nonenal (4-HNE) positive neurons.  **Stereology and Image Analysis conducted blinded to dose group?** Not stated | **Stereology: TH^+^ Neurons**  PQ had no effect on the number of TH^+^ neurons in the SNpc 7 days after the 1^st^ dose.  A statistically significant reduction in TH^+^ neurons was found in the SNpc 1 (8%), 2 (15%), 4 & 7 (25-30%) days after the 2^nd^ PQ dose. The reduction on day 7 was of no greater magnitude than that at day 4.  PQ had no further effect (reduction) on the number of TH^+^ neurons in the SNpc, 2 or 7 days after the 3^rd^ dose (reduction was 25-30%).  **Stereology: Nissl-Stained Neurons**  PQ had no effect on the number of Nissl-positive neurons in the SNpc, 7 days after the 1^st^ dose. Statistically significant reductions were observed in the number of Nissl-positive neurons in the SNpc of the PQ-treated group, 1 (5.4%), 2 (9.6%) & 4 (19.7%) days after the 2^nd^ dose and 7 days (22.6%) after the 3^rd^ dose.  **Image Analysis: 4-HNE-Positive Neurons**  Statistically increased numbers of 4-HNE- positive neurons were observed in the SNpc 2 and 4 days after the 1^st^ dose, and 1, 2, 4 and 7 days after the 2^nd^ dose.  PQ had no effect on the number of 4-HNE positive neurons in the SNpc, 1, 2, 4 or 7 days after the 3^rd^ dose. |
| McCormack (2006) [32]  **Study #26** | **Strain:** C57BL/6 mice  **Source:** Unknown  **Gender:** Male  **Age:** 8 weeks  Number of mice per PQ group (dose regimen 1) = 5 mice  **Quarantine Period:** Not provided  **Housing:** Not stated  **Number of PQ mice**  **Dose regimen 1:** ≥ 5 mice/group  **Dose regimen 2:** ≥ 4 mice/group | **PQ dichloride hydrate**  **Source:** Sigma Chemical  **PQ Dose Regimen 1:**  10 mg/kg i.p., once per week for 3 weeks.  Mice sacrificed 1 week after the 3^rd^ dose.  **PQ Dose Regimen 2:**  10 mg/kg i.p., once per week for 2 weeks.  Mice sacrificed 1, 2, 4 or 7 days after the 2^nd^ dose.  **Negative Control:** Saline  **Analytical confirmation of dosing solutions**: Not stated | **3D Stereology: TH^+^ Neurons**  40 µm sections were made through the SNpc and stained for) immune-reactive, DAB chromogen- labeled TH^+^ neurons (counterstained with cresyl violet).  1 of every 6^th^ section was evaluated as described by McCormack et al. (2002) [26]  **Stereology: Calcium Binding Protein Calbindin (CB)**  Stereology was conducted on 40 µm sections through the SNpc. 1 of every 3^rd^ section was evaluated as described by McCormack et al. (2002)[26]  **Image Analysis: 4-HNE-positive Neurons (Lipid Peroxidation)**  Single section through A9 (SNpc) or A10 (ventral midbrain tegmentum - VTA) were immnunostained for 4-hydroxy-2-n1anol (4-HNE) positive neurons as described by McCormack et al. (2005) [29].  **Stereology and Image Analysis conducted blinded to dose group?** Not stated | **Stereology: TH^+^ Neurons**  A statistically significant reduction (30%) in the mean number of TH^+^ immuno-reactive neurons was found in the SNpc of the PQ-treated group (dose regimen 1) compared to the saline control group. PQ had no effect on the number of TH^+^ neurons in the VTA.  **Stereology: Nissl-Stained Neurons**  There was a statistically significant reduction (~28%) in the number of Nissl-stained neurons in the SNpc of the PQ-treated group (Fig.2b)  **Image Analysis: 4-HNE-positive Neurons (Lipid Peroxidation)**  **Dose Regimen 1:** PQ had no effect on the number of CB-positive neurons in the SNpc or the VTA (Table 1).  **Dose Regimen 2:** There was a statistically significant increase in the mean number of 4-HNE neurons in the SNpc and VTA of PQ-treated mice 1, 2, 4 and 7 days post-treatment.  The effect of treatment more pronounced in the SNpc than in the VTA (Table1). |
| Minnema (2014) [33]  **Study #27** | **Strain:** C57BL/6 mice  **Source**: Jackson Labs (ME)  **Gender:** Male and female  **Age at receipt:** 7 weeks  **Quarantine Period:** 3 weeks  **Housing:** Individual stainless-steel, wire-mesh-floored cages    **Number of PQ Mice**  **Neuropathology**: 5 mice/sex/group killed at day 31, 59 or 94  **Stereology:** 20 mice/sex/group killed after 91-94 days of treatment  **Number of MPTP Mice - Pathology:** 5 mice/sex/occasion  **Stereology:** 20 mice/ sex | **PQ dichloride hydrate**  **Source:** Syngenta, 99.9% pure, Lot No. ASJ10083-03  **PQ Diet:** 10 ppm or 50 ppm PQCl_2_ in diet for 13 weeks.  **Average PQ ion Dose in the 10 ppm PQCl_2_ group**  Males: 1.7 mg/kg/day  Females: 2.7 mg/kg/day  **Average PQ ion Dose in the 50 ppm PQCl_2_ group**  Males: 10.2 mg/kg/day  Females:15.6 mg/kg/day  **Negative Control:** Certified Rodent Diet 5002; PMI Nutrition International  **Positive Control:** MPTP (10 mg/kg x 4 doses i.p. given 1 day at 22 weeks of age)  **Analytical confirmation of dosing solutions**: Yes | **3D Stereology**: **TH^+^/TH^-^ Neurons**  40 µm nominal section thickness (1 in 3 sections evaluated bilaterally) through the SNpc for immune-reactive, DAB chromogen-labeled TH^+^ neurons, counter-stained with cresyl violet.  Each section examined through its depth excluding guard zones.  Counting frame interval = 120 x 120 mm; counting frame size = 60 x 60 µm; disector height = 4 µm; and upper guard zone = 2 µm.    **Neuropathology**  Semi-quantitative neuropathological assessment of SNpc and striatum  • Cell Death (AmCuAg,TUNEL,  Caspase 3)  • TH^+^ Neurons: Staining intensity  • Microglia (Iba-1)  • Astrocytes (GFAP)  • Nissl staining (Thionine)  **Stereology conducted blinded to dose group?** Yes | **Stereology**: **TH^+^/TH^-^ Neurons:**  There were no effects of paraquat on the number of TH^+^ neurons or the total number of neurons in the SNpc of male or female mice administered PQCl_2_ in the diet at concentration of 10 or 50 ppm for 13 weeks.  22 week old MPTP-treated male mice displayed a statistically significant, 10% reduction in the number of TH^+^ neurons in the SNpc when assessed 7 days after dosing. The number of TH^+^ neurons in female mice was reduced by 5% but this was not statistically significant.  **Neuropathology**:  There were no effects of PQ on indicators of cell death (AmCuAg, TUNEL, Caspase 3), cell loss (TH or Nissl staining) or microglial (Iba-1) or astrocyte (GFAP) activation when assessed after 4, 8 or 13 weeks in male or female mice.  MPTP-treated male mice displayed increased mean severity scores for GFAP and Iba-1 staining in the striatum and the SNpc and TH staining was reduced. Similar results were noted in the striatum of females but no effects of MPTP treatment were detected in the SNpc. Indicators of cell death (AmCuAg, TUNEL, Caspase 3) were not detected 7 days after MPTP dosing in males or females. |
| Mitra (2011) [34]  **Study #28** | **Strain:** Swiss albino mice  **Source:** National Institute of Nutrition, Hyderabad (India)  **Gender:** Male  **Age:** 22-24 weeks  **Quarantine Period:** 1 week minimum  **Housing:** Individual cage housing  **Number of Mice:** 3/group | **Paraquat dichloride, α-tocopherol**  **Source:** Sigma Aldrich  **PQ Doses**  **Acute Experiment**  5, 10. 20, 40 or 80 mg/kg i.p. twice weekly for 4 weeks  **Repeat-Dose Experiment**  10 mg/kg i.p., twice weekly for 4 weeks  **Negative Control:** Saline  **Neuroprotection Control:** α-tocopherol administered (20 mg/kg i.p.) daily for 5 days after the last PQ dose.  **Sacrifice Schedule:** Decapitated 7 days after the last PQ dose.  **Analytical confirmation of dosing solutions**: Not stated | **TH^+^ Neurons in the SNpc**  5 µm thick sections were paraffin embedded and immunostained for DAB marked TH^+^ neurons and counterstained with hematoxylin and eosin.  TH^+^ neurons were visualized and counted under magnification using optical fractionators from MicroBrightfield and Stereo Investigator software (v. 8). The number of sections evaluated was not stated, but Pabon (2011) [35] was cited. These investigators evaluated every 3^rd^ section of the striatum and every 6^th^ section of the SNpc.  **TH, IBA1 and Mac1 Expression in the SNpc**  Densiometric analysis of western blots (N=3) was used to characterize the intensity of microglia (Iba-1) and microglial activation (Mac-1) in the SNpc after PQ treatment.  **Cell counts conducted blinded to dose group?** Not stated | **PQ Repeat Dose LD50** (Fig. 1)  100% of the mice in the 5 and 10 mg/kg dose group survived to scheduled sacrifice (Day 35). Approximately 50% of the mice in the 20 mg/kg PQ treated- group survived at least 14 days, whereas only 20% of the 40 mg/kg PQ mice survived 7 days. All mice in the 80 mg/kg died within 8 hours of the first dose.  **TH^+^ Neurons in the SNpc (Fig. 6)**  In mice surviving for an unspecified number of days after the last PQ dose, the number of TH^+^ neurons was reduced by 10, 43, 30,18 and 5% compared to saline controls in the 5, 10, 20, 40 and 80 mg/kg PQ dose groups, respectively.  Nuclei in the neurons in the SNpc were described as being pyknotic (Fig 7b); quantitative data were not provided.  TH expression (Western blots) was significantly reduced (~45%) in the SNpc of PQ treated mice compared to controls (Fig 8E).  **Microglial: Expression of Iba-1 and Mac-1 in the SNpc**  Iba-1 expression was significantly increased (~1.9-fold) in PQ treated mice and Mac-1 was increased ~ 2.1-fold (Figure 13D and 13H). |
| Norris (2007) [36]  **Study #29** | **Strain:** Wild type (WT;M7 line) mice on a C57Bl/C3H  Transgenic (Tg) mice on a C57Bl/C3H back-ground that over-expressed human α-synuclein (A53T; M83 line)  **Source:** Mouse facility at University of Pennsylvania  **Gender:** Not stated  **Age:** 3, 8 or 12 months  **Quarantine Period:** Not provided  **Housing:** Not stated  **Number of Mice:** Not stated | **Paraquat**  **Source:** Not provided  **PQ Doses**  5 mg/kg i.p., twice weekly for 3 weeks in 3 month old mice  10 mg/kg i.p. twice weekly for 3 weeks in 8 or 12 month old mice | **TH^+^ Neurons in the SNpc**  Methods not described | **TH^+^ Neurons in the SNpc**  “No noticeable cell loss in the substantia nigra or other brain regions was observed in in NTg, M7, Tg or M83 mouse lines at any age after PQ and/or MB (i.e. maneb) treatment.” |
| Peng (2004) [37]  **Study #30** | **Strain:** C57BL/6  **Source**: Jackson Labs (Bar Harbor, ME)  **Gender:** Male  **Age at receipt:** 8 weeks  **Quarantine Period:** Not provided  **Housing:** Not stated  **Number of Mice**: 4 mice/group | **PQ dichloride hydrate**  **Source:** Sigma Chemical  **PQ Dose:** 7 mg/kg/dose i.p.; once every other day (2Day intervals) for a total of 10 doses.  **Negative Control:** Saline  **Sacrifice:** 7 or 8 days after the last PQ dose  **Analytical Confirmation of Dosing Solutions:** Not stated | **Tissue Collection and Processing**  7 to 8 days after the final PQ dose, the brains were fixed *in situ* using perfusion fixation. Brains were cryoprotected in 20% sucrose and sectioned (40 μm) through the SNpc. Sections were treated with a primary antibody in order to immunolabel TH^+^ neurons (DAB chromogen) in the SNpc.  **3D Stereology: TH^+^ Neurons**  After outlining the SNpc, a computer-assisted image analysis system (optical fractionator method, Neurolucida stereo investigator, MicroBrightfield software) was used to count TH^+^ neurons in sample fields of the SNpc. Every second section throughout the SNpc was evaluated  The total number of TH^+^ neurons was estimated by multiplying the number of TH^+^ neurons with the sampled regions with the reciprocals of the fraction of the sectional area sampled and the fraction of the section thickness.  **Stereology conducted blinded to dose group?** Not stated | **Stereology of TH^+^ Neurons**  A statistically significant reduction (approximately 28%) in the mean number of TH^+^ neurons was reported for the PQ- treated mice compared to the control group (Fig 7B). |
| Peng (2005) [38]  **Study #31** | **Strain:**C57BL/6  **Source**: Jackson Labs  **Gender:** Male  **Age at receipt:** 8 weeks  **Quarantine Period:** Not provided  **Housing:** Not stated  **Number of PQ Mice**: 4 to 5 mice | **PQ dichloride hydrate**  **Source:** Sigma Chemical  **PQ Dose:** 7 mg/kg/dose i.p.; once every other day (2Day intervals) for a total of 10 doses.  **Negative Control:** Saline  **Sacrifice:** 7 or 8 days after the last PQ dose  **Analytical Confirmation of Dosing Solutions:** Not stated | **Tissue Collection and Processing**  7 to 8 days after the final PQ dose, the brains were fixed *in situ* using perfusion fixation. Brains were cryoprotected in 20% sucrose and sectioned (40 μm) through the SNpc. Sections were treated with a primary antibody in order to immunolabel TH^+^ neurons (DAB chromogen) in the SNpc.  **3D Stereology: TH^+^ Neurons**  After outlining the SNpc, a computer-assisted image analysis system (optical fractionator method, Neurolucida stereo investigator, MicroBrightfield software) was used to count TH^+^ neurons in sample fields of the SNpc. Every 2^nd^ section throughout the SNpc was evaluated.  The total number of TH^+^ neurons was estimated by multiplying the number of TH^+^ neurons with the sampled regions with the reciprocals of the fraction of the sectional area sampled and the fraction of the section thickness.  **Stereology conducted blinded to dose group?** Not stated | **Stereology of TH^+^ Neurons**  A statistically significant reduction (approximately 28%) in the mean number of TH^+^ neurons was reported for PQ-treated mice compared to the control group (Fig 5B). |
| Peng (2007) [39]  **Study #32** | **Strain:** C57BL/6  **Source**: Jackson Labs  **Gender:** Male  **Age at receipt:** 8 weeks  **Quarantine Period:** Mice were maintained in the laboratory for 2, 6, 12, or 24 months  **Housing:** Not stated  **Number of PQ Mice**: 4 or 5 mice at each age | **PQ dichloride hydrate**  **Source:** Sigma Chemical  **PQ Dose**  2, 6, and 12 month-old mice: 10 mg/kg/dose i.p.  24 month-old mice: 8 mg/kg/ dose i.p.  All mice were dosed twice weekly for 3 weeks for a total of 6 doses.  **Negative Control:** Saline  **Sacrifice:** 4 or 7 days after the last PQ dose  **Analytical Confirmation of Dosing Solutions:** Not stated | **Tissue Collection and Processing**  7 to 8 days after the final PQ dose, the brains were fixed *in situ* using perfusion fixation. Brains were cryoprotected with 20% sucrose and sectioned (40 μm) through the SNpc. Sections were treated with a primary antibody in order to immunolabel TH^+^ neurons (DAB chromogen) in the SNpc.  **3D Stereology: TH^+^ Neurons**  After outlining the SNpc, a computer-assisted image analysis system (optical fractionator method, Neurolucida stereo investigator, MicroBrightfield software) was used to count TH^+^ neurons in sample fields of the SNpc. Every 2^nd^ section throughout the SNpc was evaluated.  The total number of TH^+^ neurons was estimated by multiplying the number of TH^+^ neurons with the sampled regions with the reciprocals of the fraction of the sectional area sampled and the fraction of the section thickness.  **Double Labeling for 3-nitrotyrosine**  In addition to labeling of TH^+^ neurons, the tissue sections were treated with anti-3-nitrotyrosine (3-NT) and then secondary antibodies in order to determine if PQ treatment altered 3-NT levels.  **Stereology conducted blinded to dose group?** Not stated | **Stereology of TH^+^ Neurons**  A statistically significant reduction (approximately 30%) in the number of TH^+^ neurons was reported for the PQ treated group compared to the control group, at all ages (2, 4, 12 and 24 months; Fig 3).  **Double Labeling (3-NT)**  PQ administration at 2 months of age resulted in an increased 3-NT levels that were further increased at 12 months of age. |
| Peng (2009) [40]  **Study #33** | **Strain:**C57BL/6  **Source**: Jackson Labs  **Gender:** Male  **Age at receipt:** Mice were bred in-house  **Quarantine Period:** Mice were maintained in the in-house facility until 2 or 12 months of age.  **Housing:** Not stated  **Number of PQ Mice**: 3 to 5 mice at each age | **PQ dichloride hydrate**  **Source:** Sigma Chemical  **PQ Dose:** 10 mg/kg/dose.  Mice were dosed twice weekly for 3 weeks for a total of 6 doses, beginning at either age 2 or 12 months of age.  **Negative Control:** Saline  **Sacrifice:** 4 days after the last PQ dose  **Analytical Confirmation of Dosing Solutions:** Not stated | **Preparation of Brain Sections**  Fixed brain sections were incubated with anti-hp91^phox^ and anti-CD11b primary antibodies. The sections were then treated with the secondary antibodies. Multicolor images were scanned using different lasers. IMARIS (Bitplane AG) imaging software was used for 3-dimensional reconstruction.  CD11b was used as a marker of activated microglia, and gp91^phox^ was used as a biomarker for NADPH oxidase.  **Analyses conducted blinded to dose group?** Not stated | **Activated Microglia**  An increase of approximately 463% in the number of activated microglia was noted for the 2-month-old, PQ-treated mice.  An increase of approximately 787% in the number of activated microglia was noted for the 2-month-old, PQ-treated mice.  **NADPH oxidase**  Co-localization of activated microglia with gp91^phox^ (NADPH oxidase levels) was increased by approximately 584% in the 2 month old PQ mice, and by approximately 582% in the 12 month-old, PQ-treated mice. |
| Peng (2010) [41]  **Study #34** | **Strain:** Cross between Swiss Webster and C57BL/6  **Source**: Unclear–reference made to Matsuoka et al. (2001) [25].  **Gender:** Male  **Age at receipt:** Mice were bred in-house  **Quarantine Period:** Mice were maintained in the in-house facility until 2, 12 or 23 months of age.  **Housing:** Not stated  **Number of PQ Mice**: 4 to 5 mice at each age | **PQ dichloride hydrate**  **Source:** Sigma Chemical  **PQ Dose:** 10 mg/kg/dose for the 2-month old mice, 9 mg/kg/dose for the 12-month old mice, and 8 mg/kg/dose for the 23-month old mice.  Mice were dosed twice weekly for 3 weeks for a total of 6 doses  **Negative Control:** Saline  **Sacrifice:** 7 days after the last PQ dose  **Analytical Confirmation of Dosing Solutions:** Not stated | **Tissue Collection and Processing**  7 days after the final PQ dose, the brains were fixed *in situ* using perfusion fixation. Brains were cryoprotected in 20% sucrose and sectioned (40 μm) through the SNpc. Sections were treated with a primary antibody in order to immunolabel TH^+^ neurons (DAB chromogen) in the SNpc.  **3D Stereology: TH^+^ Neurons**  After outlining the SNpc, the number of TH^+^ in sample fields was counted in every third section throughout the SNpc using a computer-assisted image analysis system (optical fractionator method, Neurolucida stereo investigator, MicroBrightfield).  **Double Labeled for 3-nitrotyrosine**  Tissue sections were treated with anti-3-nitrotyrosine (3-NT) and then with secondary antibodies.  **Stereology conducted blinded to dose group?** Not stated | **Stereology of TH^+^ Neurons**  A statistically significant reduction of approximately 25 to 27% in the number of TH^+^ neurons was reported for the PQ treated group compared to the control group at 2, 12 and 23 months of age.  **Double Labeling (3-NT):**  3-NT immunofluorescence was used as a biomarker for protein oxidation. PQ administration at 2, 12, and 23 months of age resulted in an increased mean number of TH^+^ neurons that had co-localized 3-NT staining (increased by approximately 555, 516, and 401%, respectively). |
| Prakash (2013) [42]  **Study #35** | **Strain:** Swiss albino  **Source**: Institute of Medical Science, Varanasi (India)  **Gender:** Male  **Age at receipt:** Not provided  W8: ~25 g  **Quarantine Period:** Not provided  **Housing:** Not stated  **Number of Mice**  6 mice in each of the control and PQ-treated groups | **PQ dichloride hydrate**  **Source:** Sigma Aldrich  **PQ Dose:** 10 mg/kg i.p. injection, twice weekly for 9 weeks    **Negative Control:** Saline  **Sacrifice:** Mice were perfused with fixative an undefined number of days after the last dose.  **Analytical confirmation of dosing solutions**: Not stated | **Tissue Collection and Processing**  An undefined number of days after the final PQ dose, the brains were fixed in situ using perfusion fixation. Brains were cryoprotected in 20% sucrose and sectioned (20 μm) through the SNpc. Sections were treated with a primary antibody in order to immunolabel TH^+^ neurons (DAB chromogen) in the SNpc.  **TH^+^ Neuronal Assessment**  Sections were evaluated using bright field microscopy. Images were captured at a 10x magnification and TH^+^ neurons were counted using an undisclosed method.  **Assessment conducted blinded to dose group?** Not stated | **Number of TH^+^ Neurons**  The mean number of TH^+^ neurons in the SNpc was statistically significantly reduced in PQ treated mice by ~ 55% compared to the control group. |
| Prasad (2009)  [43]  **Study #36**  **Experiment 1** | **Strain:**C57BL/6  **Source**: Jackson Labs (Bar Harbor, ME)  **Gender:** Male  **Age at receipt:** 8-12 weeks  **Quarantine Period:** Not provided  **Housing:** Not stated  **Number of PQ Mice** **6, 12, 18 or 24 Dose Subgroup**: 6 to 8 mice/group | **PQ dichloride hydrate**  **Source:** Sigma Chemicals  **PQ Dose:** 10 mg/kg i.p. injection, twice, or 3 times weekly for a total of 6, 12, 18 or 24 doses.    **Negative Control:** Saline  **Sacrifice:** Mice killed 7 days after the last PQ dose  **Analytical confirmation of dosing solutions**: Not stated | **Western Blot Analysis of TH in Striatum**  Striatal samples were collected and homogenized in buffer, centrifuged, re-suspended in buffer and subjected to polyacrylamide gel electrophophoresis.  Densitometric values for TH were normalized as a ratio to densitometric values for α-tubulin. | **Western Blot: TH Levels in Striatum (Fig. 5)**  The mean TH/α-tubulin ratio was significantly greater in PQ-treated groups after 6 (~44%) or 12 (~31%) doses compared to the vehicle controls, irrespective of dose frequency (2 doses or 3 doses/week).  The results highly variable after 18 or 24 doses and the group mean ratios in the PQ-treated mice were not statistically significantly different from controls. |
| Purisai (2007) [44]  **Study #37** | **Strain**: C57BL/6 mice  **Source:** Charles River (CA) and Jackson Labs (ME)  **Gender:** Male  **Age**:10-12 weeks  **Quarantine Period**: Not provided    **Housing:** Not stated  **Number of PQ Mice Evaluated:** Not stated | **PQ dichloride hydrate**  **Source:** Sigma Chemical  **PQ Dose:** 10 mg/kg i.p.,  Once weekly for 1 or 2 weeks  **Negative Control:** Saline  **LPS Priming:** 2 or 4 mg/kg LPS prior to PQ dose  **Anti-inflammatory Control:** 45 mg/kg minocycline prior to PQ dose and twice daily for 3 or 7 days.  **Sacrifice:**  **Stereology of TH^+^ Neurons:**  7 days after the last PQ dose  **Image Analysis of Mac-1 Positive Microglia:** 1, 2, 4 or 7 days after the last PQ dose  **Analytical Confirmation of Dosing Solutions:** Not stated | **3D Stereology: TH^+^ Neurons**  40 µm sections through the SNpc were stained for immune-reactive, (DAB chromogen- labeled), TH^+^ neurons that were counter-stained with cresyl violet.  **Image Analysis of Mac-1-positive microglia**  Mac-1 immuno-reactive microglia with enlarged cell body and swollen processes were counted in SNpc (midbrain sections anterior to the 3^rd^ nerve), 1, 2, 4 or 7 days after the PQ dose). The details on the counting procedure not provided.  **Stereology and Image Analysis conducted blinded to dose group?** Not stated | **Stereology: TH^+^ Neurons**  There was a statistically significant reduction in the mean number of TH^+^ neurons in the SNpc, 7 days after 2 10 mg/kg doses of PQ (Fig 2B and Fig 6) but not after a single dose (Fig 5).  There was no effect of PQ on the mean number of TH^+^ neurons in mice treated with minocycline.  A statistically significant reduction in the mean number of TH^+^ neuron was observed in the SNpc of mice that were pretreated with LPS and then given a single 10 mg/kg PQ dose.  **Image Analysis of MAC-1-positive microglia**  A statistically significant increased number of Mac-1-labeled, activated microglia was observed in the SNpc, 1, 2, 4 or 7 days after PQ administration.  There was no significant effect of PQ on Mac-1 activated microglia when mice were pre-treated with minocycline. |
| Rappold (2011) [45]  **Study #38** | **Strain**: WT C57BL/6 mice  **Source:** Charles River Laboratories  DAT_HOM_ mice (Partial loss of DAT function)  OCT3^+^/^+^ and OCT-/- mice  **Source:** Columbia University Transgenic facility  **Gender:** Male  **Age**: 10-12 weeks  **Quarantine Period**: Not provided    **Housing:** Not stated  **Number of PQ Mice:**  **TH Immunostaining - Striatum**: 4 to 5 mice  **TH^+^ Stereology - SNpc**: 5 | **PQ dichloride hydrate**  **Source:** Sigma Chemical  **PQ Dose:** 10 mg/kg i.p., every second day for a total of 10 dose  **Negative Control:** Saline  **Dopamine Transporter** **Control:** (DA-HOM) or OCT3 ^+^/^+^ or OCT-/-    **Sacrifice:** 7 days after last PQ dose by transcardial perfusion  **Analytical Confirmation of Dosing Solutions:** Not stated | **TH^+^ Neuron Assessment in the SNpc**  Method not described.  **TH Optical Density in the Striatum**  30 µm thick sections through the striatum were immune-stained with antibodies for TH and Alexa Fluor 498 and 594. The optical density (OD) of TH immune-reactivity was evaluated by scanning sections at successive 0.5 µm depths throughout the whole section using confocal microscopy.  The densiometric method used to quantify TH immune-reactivity was not described.  **Stereology and Image Analysis conducted blinded to dose group?** Not stated | **TH*^+^* Neurons in the SNpc : OCT^+^/^+^ and OCT-/- (Fig. 1a)**  The mean number of TH^+^ neurons in the SNpc was statistically significantly reduced (~22%) in PQ treated OCT^+^/^+^ and OCT-/- mice compared to their respective saline controls.  The total number of Nissl-stained neurons in the SNpc of PQ-treated mice (12,364 ± 522) was reduced ~ 20% compared to saline controls (15,438 ± 532).  **TH*^+^* Neurons in the SNpc : DAT_HOM_ vs. DAT_WT_ (Fig. 5D)**  The mean number of TH^+^ neurons in the SNpc was statistically significantly reduced (~36%) in PQ-treated, DAT_WT_ but not DAT_HOM_ compared to saline controls.  **TH Optical Density (OD) in the Striatum**  There was no effect of PQ treatment on TH^+^ immune-reactivity in DAT-WT male mice compared to saline controls (Supplemental Fig S4).  There was no effect of PQ on TH immuno-reactivity (OD) in OCT3^+/+^ PQ treated mice compared to OCT3^+/+^ saline controls (Fig 1B).  PQ-treated OCT-/- mice had a significant reduction (~34%) in striatal TH immuno-reactivity (OD) compared to OCT^-/-^ saline controls (Fig 1B). |
| Reeves (2003) [46]  **Study #39** | **Strain**: WT C57BL/6 mice  **Source:** Not stated    **Gender:** Male  **Age**: 10 weeks  **Quarantine Period**: 4 weeks    **Housing:** Not stated  **Number of PQ Mice:** 6 per group | **PQ dichloride hydrate**  **Source:** Sigma Chemicals  **PQ Dose:** 10 mg/kg i.p. injection, twice weekly for 12 weeks.    **Negative Control:** Saline  **Sacrifice:** Mice killed by intracardial perfusion with 4% paraformaldehyde, 7 days after the last PQ dose.  **Analytical confirmation of dosing solutions**: Not stated | **TH Immune-reactivity in the Striatum**  40 µm sections through the striatum were collected in cyroprotectant. Sections were incubated overnight at 4^o^ C with a primary antibody to TH followed by a secondary antibody and DAB as the chromogen. Densitometric values were obtained for the immune-reactive TH chromogen.    **Western Blot Analysis of TH in Striatum**  Samples from the dorsal striatum (projection region for the nigrostriatal DA system) and the nucleus accumbens (control region) were collected and homogenized in buffer, centrifuged, re-suspended in buffer and subjected to polyacrylamide gel electrophoresis for TH. Densiometric values for TH were determined. | **TH Levels in Striatum (Fig. 6)**  Mean TH immune-reactivity in the dorsal striatum was statistically significantly less (~13%) in the PQ-treated groups 7 days after the after the last dose compared to the vehicle controls.  Mean TH densiometric levels in the striatum were decreased by ~47% in the PQ-group based on Western blots. The difference was marginally statistically significant (p < 0.53) |
| Ren (2009) [47]  **Study #40** | **Strain**: C57BL/6 mice  **Source:** Not stated    **Gender:** Male  **Age**: 8 weeks  **Quarantine Period**: Not provided    **Housing:** Not stated  **Number of PQ Mice:** 8 per group | **PQ dichloride hydrate;**  **MPTP**  **Source:** Sigma Chemical (USA)  **PQ Dose:** 10 mg/kg/day administered by oral injection daily for 4 months.  **Negative Control:** Saline  **MPTP Positive Control:** 10 mg/kg/day administered daily by oral injection for 4 months.    **Sacrifice:** Mice were anaesthetized and killed by transcardial perfusion.  **Analytical Confirmation of Dosing Solutions:** Not stated | **Optical Density (OD) of TH^+^ Neurons in the SNpc**  30 µm sections through the SNpc were immunostained for TH using a DAB chromogen.  Integrated optical density was measured; neither the number of sections that were evaluated nor the method used were provided.  **Image Analysis conducted blinded to dose group?** Not stated | **Optical Density (OD) of TH^+^ Neurons in the SNpc**  The integrated OD of TH immuno-reactive neurons in PQ-treated mice (2788.0±367.5) was reduced by 48.7% compared to saline controls (5439.1± 453.2).  The OD for immune-reactive-TH neurons in the SNpc was reduced by 46.6% in MPTP-treated mice compared to saline controls. |
| Rojo (2007) [48]  **Study #41** | **Strain**: WT C57BL/6 mice  **Source:** Charles River International (MA)    **Gender:** Male  **Age**: 8 weeks  **Quarantine Period**: Not provided  **Housing:** Not stated  **Number of PQ Mice:** 4 per group | **PQ dichloride hydrate**  **MPTP**  **Source:** Sigma Chemical (Spain)  **PQ Dose:** 10, 20 or 30 mg/kg/day administered intra-nasally for 30 days at a total volume of 20 µl/mouse/day (10 µl/nostril) for a 20 gram mouse; Body W8 = 33 g.  **PQ Concentration:** 25, 50, 75 µg/µl injected daily for 4 months.  **Negative Control:** Saline  **MPTP Positive Control:** 30 or 60 mg/kg/day administered by intranasal injection, daily for 4 months.    **Sacrifice:** Anaesthetized and killed by transcardial perfusion.  **Analytical Confirmation of Dosing Solutions:** Not stated | **Number of TH^+^ Neurons in the SNpc**  40 µm thick sections through the SNpc and striatum were immunostained for TH using the DAB chromogen.  The number of TH^+^ neurons in the SNpc was counted in 1 in approximately every 7.5 sections (i.e. every 300 µm); The total number of TH^+^ neurons in the SNpc was estimated (specific method not provided; a method described for counting the number of TH^+^ neurons in human brain was cited).  **TH^+^ Immune-reactivity in Striatum**  “Representative photomicrographs” of sections through the striatum were presented. TH^+^ immune-reactivity was not quantified.  **Stereology and Image Analysis conducted blinded to dose group?** Not stated | **Number of TH^+^ Neurons in the SNpc (Fig. 2B)**  PQ had no effect on the number of TH^+^ neurons in the SNpc at doses up to 30 mg /kg/day administered for 30 days.  In the positive control, MPTP (60 mg/kg/day), a statistically significant (~71%) reduction in TH^+^ neurons in the SNpc was found.  **TH^+^ Immunostaining of Striatum (Fig 2A)**  There was no apparent effect of PQ on TH immunostaining of the striatum compared to controls in the “representative section”  TH immunostaining was apparently reduced in a MPTP mouse compared to a “representative control mouse. |
| Srivastava (2012) [49]  **Study #42** | **Strain**: Swiss Webster mice  **Source:** CSIR Indian Institute of Toxicology Research (Lucknow)    **Gender:** Male  **Age**: Not stated  **W8:** 20-25 grams  **Quarantine Period**:  Not provided  **Housing:** Not stated  **Number of PQ Mice Evaluated:** 9-12 mice per group  **Number of Replicates**: 3 to 4 | **PQ dichloride hydrate**    **Source:** Sigma-Aldrich (St. Louis, MO)  **PQ Dose:** 10 mg/kg/day i.p. administered twice weekly for 9 weeks.  **Negative Control:** Saline  **Sacrifice:** Killed by trans-cardial perfusion or cervical dislocation  **Analytical Confirmation of Dosing Solutions:** Not stated | **Number of TH/NeuN^+^ or Fluro-Jade B/DAPI^+^ Neurons in the SNpc**  20 µm thick sections through the SNpc were immunostained for TH using the DAB chromogen and counterstained with NeuN.  In a second subset of animals, sections through the SNpc were immunostained with Fluoro-Jade B and DAPI.  The number of TH/NeuN^+^ or Fluro-Jade B/DAPI neurons in the SNpc were counted bilaterally in 2, 100 x 100 µm frames selected for each of 3 sections/animal. This was replicated in 3 or 4 experiments (i.e. 9-16 mice).  The mean number of TH^+^ or NeuN^+^ neurons in the PQ group was expressed as a percentage of the control group.  The mean number of Fluro-Jade B/DAPI neurons in the control and PQ-treated group were expressed as a percentage of the total number of neurons.  **Stereology and Image Analysis conducted blinded to dose group?** Yes | **Number of TH/NeuN^+^ Neurons in the SNpc**  The mean number of TH/NeuN^+^ neurons in the SNpc of PQ treated mice was significantly reduced (~36%) compared to the saline controls.  **Number of Fluro-JadeB/DAPI^+^ Neurons in the SNpc**  The percent of total neurons in the SNpc that stained positive for Fluro-JadeB/DAPI in PQ treated mice was significantly increased (~2.2 fold) compared to the saline controls. |
| Su *(*2015) [50]  **Study #43** | **Strain**:C57BL/6NCrIVr mice    **Source:** Not stated    **Gender:** Male  **Age**: 8 weeks  **Quarantine Period**: Not provided    **Housing:** Not stated  **Number of PQ Mice:** 5 to 6 per group | **PQ dichloride hydrate**    **Source:** Sigma-Aldrich (St. Louis, MO)  **PQ Dose:** 10 mg/kg/day i.p. administered twice weekly for 4 weeks.  **Negative Control:** Saline  **Sacrifice:** Mice were killed 48 hours after the last dose; the method used not stated.  **Analytical Confirmation of Dosing Solutions:** Not stated | **Number of TH^+^ Neurons in the SNpc**  4 µm thick sections through the SNpc were immunostained for TH using the DAB chromogen.  The number of TH^+^ neurons in sections through the SNpc was counted under bright field illumination. A t**o**tal of 5 to 6 sections were evaluated per mouse. No details were provided on the size or selection of counting frames. Approximately 155 TH^+^ neurons were counted in the control group.    **Stereology and Image Analysis conducted blinded to dose group?** Not stated | **Number of TH^+^ Neurons in the SNpc**  The mean number of TH^+^ neurons in the SNpc was significantly reduced (~39%) in PQ-treated mice compared to the saline controls. |
| Thiruchelvam (2000a) [51]  **Study #44** | **Strain**: C57BL/6 mice  **Source:** Taconic (Germantown, NY)  **Gender:** Male  **Age**: 6 weeks  **Quarantine Period**:1 week minimum  **Housing:** 1 mouse per cage  **Number of PQ mice:** 5 per group | **PQ dichloride hydrate**  **Source:** Sigma Chemical (St. Louis, MO)  **PQ Dose:** 5 or 10 mg/kg i.p., once weekly for 4 weeks  **Negative Control:** Saline  **Sacrifice:** Mice killed 3 days after the last PQ dose  **Analytical confirmation of dosing solutions**: Not stated | **Tissue Collection and Processing:**  **Striatum and nucleus accumbens**  3 days after the last PQ dose, brains were frozen and 6, 40 μm sections through the striatum, and 4 sections through the nucleus accumbens, were immunostained (DAB chromogen) for TH^+^ neurons.  **Image Analysis:** TH^+^ neurons in striatum and nucleus accumbens.  Staining intensity for TH, from 2 different regions on each slide (30% of total area) was averaged to give a total mean density for TH. Background intensity was measured from a region of the slide that stained negative for TH.  **Image Analysis conducted blinded to dose group?** Yes  . | **Image Analysis for TH Density**  There were no statistically significant effects of PQ on TH density in the striatum or nucleus accumbens at PQ doses of 5 or 10 mg/kg. |
| Thiruchelvam (2000b) [52]  **Study #45** | **Strain**: C57BL/6 mice  **Sources:** Taconic (Germantown, NY) (6-week old mice)  NIA/Harlan (IN) (5-month old mice)  **Gender:** Male  **Age:** 6 weeks and 5 months  **Quarantine Period**: At least 1 week  **Housing**: 5 mice per cage  **Number of PQ Mice:**  **Image Analysis/ TH^+^ and GFAP Counts:** 6 mice  **Western blot of TH in the striatum**: 8 mice | **PQ dichloride hydrate**  **Source:** Sigma Chemical (St. Louis, MO)  **PQ Dose:** 10 mg/kg i.p., twice/week for 6 weeks.  **Negative Control:** Saline  **Positive Control:** N1 specifically for the TH or GFAP- analyses  **Sacrifice:** Killed 5 days after the last PQ dose  **Analytical confirmation of dosing solutions**: Not stated | **Tissue Collection and Processing:**  **Striatum and nucleus accumbens**  5 days after the last PQ dose, brains were frozen and 6, 40 μm sections through the striatum and 4 sections through the nucleus accumbens were immunostained for TH^+^ neurons (DAB chromogen) and counter-stained with cresyl violet; Additional sections through the striatum (number of sections not given) were immunostained for GFAP.  Western blots of TH in the striatum were prepared and quantified for 8 mice.  **SNpc and VTA:** 5 days after the last PQ dose, brains were frozen and 5 representative, 40 μm sections through the striatum and 2 sections through the VTA were immunostained (DAB chromogen) for TH^+^ neurons.  **Image Analysis:** TH^+^ neurons in striatum and nucleus accumbens.  Staining intensity for TH, from 2 different regions on each slide (30% of total area) was averaged to give a total mean density for TH as described by Thiruchelvam et al. (2000a) [51].  **Number of TH^+^ neurons in the SNpc and VTA:** The mean number of TH^+^ neurons per section was calculated from 3 representative sections.  **GFAP Positive Astrocytes:** The mean number of resting and activated (clusters) of astrocytes per section was calculated.  **Image and Cell Counts conducted blinded to dose group?** Yes | **Image Analysis, Western Blots and TH^+^ and GFAP Positive Cell Counts**  Compared to the saline control group, there were no statistically significant effects of 10 mg/kg PQ administered twice weekly for 6 weeks on:   - the density of TH^+^ labeling in the striatum or nucleus accumbens (Fig 5A) - the intensity of the Western blot TH bands in the striatum (Fig. 6) - the mean density of TH^+^ staining in the SNpc or VTA. (Fig.7) - the mean number of TH^+^ neurons in the SNpc or VTA (Fig. 7) - the average total number of astrocytes (resting) or clusters of astrocytes (activated) in the striatum (Table 1) |
| Thiruchelvam (2002) [53]  **Study #46** | **Strain:** C57BL/6 mice  **Source**: Unknown  **Gender:** Male  **Age:** PND 5 or 6.5 months of age  **Quarantine Period:** 1 week  **Housing:**  **PND 1-21:** Group- housed with dam and littermates    **Post-Weaning:** 4 mice/cage; cage type not provided    **Number of PQ Mice**: 4 mice  **Pups/PQ Groups:** 4-5; no more than 2 pups from each litter | **PQ dichloride hydrate**  **Source:** Sigma Chemical (St. Louis, MO)  **PQ Dose of F1 generation Pups:** 0.3 mg/kg i.p., daily from PND 5 to19 (15 doses)    **PQ Dose of F1 generation Adults:** 10 mg/kg i.p. twice week for 3.5 weeks (7 doses)  **Negative Control:** Saline  **Sacrifice:** Mice were killed 2 weeks after the last adult dose irrespective of whether mice were only administered PQ from PND 5-19 and/or as adults.  **Analytical confirmation of dosing solutions**: Not stated | **Tissue Collection and Processing: SNpc**  2 weeks after the last adult PQ dose, brains were frozen and 30 μm sections through the SNpc were immunostained for TH^+^ neurons (DAB chromogen) and counter-stained with cresyl violet.  **3D Stereology: TH^+^ Neurons**  30 µm nominal sections through the SNpc were stained for immune-reactive, DAB chromogen-labeled TH^+^ neurons.  1 in every 4 sections was evaluated bilaterally. The entire depth of field was sampled excluding a 1.5 μm upper and lower guard z1. The thickness of each section was measured and an optical fractionation method (MCID Imaging Program; Imaging Research, St. Catherines, ON) was used to calculate the total number of TH^+^ neurons in the SNpc  **Nissl-Stained Neurons**  The sections described above were counterstained with cresyl violet. The total number of TH^-^ neurons in the SNpc was calculated (TH^-^ = Nissl^+^ minus TH^+^)  **Stereology conducted blinded to dose group?** Not stated | **Stereology: TH^+^ or Nissl^+^ Neurons**  **PQ Exposure: PND 5-19 (Fig. 5)**  A statistically significant, ~16% reduction in the number of TH^+^ neurons was observed in mice administered PQ from PND 5-19 and evaluated as adults. The number of Nissl-stained neurons was reduced by ~9% in the PQ group compared to controls but this difference was not statistically significant.  **PQ Exposure: PND 5-19 & 6.5 Months (Fig. 5)**  A statistically significant ~41% reduction in the number of TH^+^ neurons was observed in mice administered PQ from PND 5-19 and as adults. The number of Nissl-stained neurons was not reduced.  **PQ Exposure: 6.5 Months (Fig. 5)**  A statistically significant, ~24% reduction in the number of TH^+^ neurons was observed in mice administered PQ as adults. The number of Nissl-stained neurons in the PQ group was comparable to controls. |
| Thiruchelvam (2003) [54]  **Study #47**  Watson (2013) [55]  **Study #48** | **Strain:** C57BL/6 mice  **Source**: NIA/Harlan  **Gender:** Male  **Ages:** 6 weeks, 5 months and 18 months of age  **Quarantine:** At least 1 week  **Housing:** 4 mice/cage; cage type not provided    **Number of PQ Mice Stereology:** 3 to 5 mice  **Western blots and TH Activity:** 8 mice    **Strain:** C57BL/6 WT  PACAP KO **(**Pituitary adenylyl cyclase activating polypeptide)  **Source**: University of California (Los Angeles, CA)  **Gender:** Male  **Age:** 2-3 months  **Quarantine Period:** Not provided  **Housing:** Not provided    **Number of PQ Mice** **Stereology:** 6 mice  **TH IR:** 6 mice  **Microglia (Iba-1):** 6 to 9 mice | **PQ dichloride hydrate**  **Source:** Sigma Chemical (St. Louis, MO)  **PQ Dose:** 10 mg/kg i.p. twice weekly for 3 weeks  **Negative Control:** Saline  **Sacrifice:** Mice killed 2 weeks or 3 months after the last dose  **Analytical confirmation of dosing solutions**: Not stated  **PQ dichloride hydrate**  **Source:** Sigma Chemical (St. Louis, MO)  **PQ Dose:** Single 10 mg/kg i.p. dose  **Negative Control:** Saline  **Sacrifice:** Mice killed 7 days after PQ dose administration by transcardial perfusion.  **Analytical confirmation of dosing solutions**: Not stated | **TH Enzyme Activity in Striatum**  Measured 2 weeks after the last dose after 6 weeks, 5 months and 18 months (n=8)  **TH in Striatum - Western Blots** Prepared and quantified for 8 mice, sacrificed 2 weeks and 3 months after PQ doses administered at 6 weeks, 5 months or 18 months.  **Tissue Collection and Processing of the SNpc for Stereology**  5 days after the last PQ dose, brains were frozen and 40 μm sections through the striatum were immune-stained for TH^+^ neurons (DAB chromogen) and counter-stained with cresyl violet.  **3D Stereology: TH^+^ and TH^-^ Neurons in the SNpc:** 40 µm nominal section thickness (sectioned bilaterally) through the SNpc were evaluated (1 in 6 sections) using the MicroBrightfield Stereo Investigator software. The number of TH^+^ and TH^-^ neurons in the SNpc was estimated.  **Stereology conducted blinded to dose group?** Not stated  **Tissue Collection and Processing: SNpc**  7 days after the PQ dose, brains were frozen and 40 μm sections through the striatum were immune-stained for TH^+^ neurons (DAB chromogen) and counter-stained with cresyl violet.  **3D Stereology: TH^+^ and TH^-^ Neurons in the SNpc:** MicroBrightfield Stereo Investigator software was used to calculate the total number of TH^+^ and TH^-^ neurons in the SNpc; 1 in every 4^th^ section was evaluated bilaterally in medial-lateral, dorsal-ventral regions. Guard Z1 = 1.5 µm at top and bottom of section. ~ 300 objects counted; Gunderson CE < 0.1.  **TH Immune-reactivity in the Striatum**  Rostral, medial and caudal sections through the striatum were stained with a primary antibody to TH and a secondary immunofluorescence antibody. Sections were scanned and TH IR was quantified using ImageJ to characterize pixel intensity.  **Microglial Activation**  The number of microglia present in standardized frames was determined and microglial size was measured in Iba-1stained sections. Microglia cell diameter was used to distinguish resting from active microglia.  **Analyses conducted blinded to dose group?** Yes | **TH Activity in Striatum (Fig. 5)**  TH activity in the striatum was statistically significantly increased at 6 weeks (~21%) and 18 months (~28%) and elevated (~36%), but not statistically significantly, in the 5-month, PQ-treated mice.  **TH in Striatum - Western Blots (Fig. 6)**  The mean amount of TH protein in the striatum was quantified in controls was comparable to mice administered PQ at 6 weeks or 5 months of age and sacrificed 2 week or 3 months later. In mice administered PQ at 18 months, TH protein levels in PQ-treated mice were reduced by ~5% (not statistically significant.), 2 weeks after the last dose and significantly reduced (~17%.) 3 months after the last dose.  **Stereology: TH^+^ Neurons in the SNpc (Fig. 7)**  The mean number of TH^+^ neurons in the SNpc was statistically significantly reduced in mice sacrificed 2 weeks after the last PQ dose aged 6 weeks (~29%), 5 months (30%) and 18 months (37%). The magnitude of the reductions was slightly greater (~35%, ~34% and ~42%) in 6 week, 5 month and 18 month old mice respectively sacrificed 3 months after the last PQ dose.  **Stereology**: **Nissl-Stained Neurons in the SNpc**  Data not provided  **Stereology**  There was no effect of a single dose of PQ on the mean regional, or total number of TH^+^ neurons in the SNpc of 6 WT mice. The mean number of Nissl-positive neurons in the SNpc was also comparable between the PQ-treated and the vehicle control group.   \| SNpc \| Control \| PQ \| \| --- \| --- \| --- \| \| Dorsal \| 5989±558 \| 4793±810 \| \| Ventral \| 2435±251 \| 2287±326 \| \| Medial \| 4202±308 \| 3887±488 \| \| Lateral \| 1049± 157 \| 2435±251 \| \| Total \| 13675±319 \| 13402±396 \|   **TH IR**  The mean number of TH^+^ neurons in PACAP KO mice was significantly less (~31%) in PQ-treated mice (8966±690) compared to KO vehicle controls mice (12966±1518).  There was no effect of PQ on striatal TH immune-reactivity in the WT mice whereas the average immune-reactivity for TH was significantly increased (~13%) in PACAP KO, PQ-treated mice compared to KO controls.  **Microglia**  An increased number of activated microglia was observed in PQ-treated WT but not in PQ-treated PACAP KO mice. |
| Yin (2011) [56]  **Study #49** | **Strains:**  Inbred C57BL/6J  Inbred DBA/2  **Source**: University of Tennessee HSC  **Gender:** Male, female  **Age:** 2.5 to 4 months  **Quarantine Period:** Not provided  **Housing:** Not provided    **Mice/PQ group**:  **Stereology**: 4 to 7 mice | **PQ dichloride hydrate**  **Source:** Sigma Chemical  **PQ Dose**:1 or 5 mg/kg i.p., once weekly for 3 weeks  **Negative Control:** Saline  **Sacrifice:** Mice killed 24 hours after the last dose.  Mice used for stereological assessment (Experiment 2) were killed either by exposure to carbon dioxide. Brains were maintained frozen (-80^o^C) until evaluated.  **HPLC confirmation of dosing solutions**: Not stated | **Tissue Collection and Processing: SNpc**  Twenty four hours after the last PQ dose, brains were frozen and 40 μm sections through the striatum were immunostained for TH^+^ neurons (DAB chromogen) and counter-stained with cresyl violet.  **3D Stereology: TH^+^ and TH- Neurons in the SNpc**  A total of 4 sections (1 in 8 sections) through the SNpc were evaluated unilaterally for immune-reactive, DAB chromogen-labeled, TH^+^ neurons. The entire depth of field was sampled excluding a 0.5 μm upper and lower guard z1.  An optical fractionation method (Stereo Investigator, MicroBrightfield) was used to calculate the total number of TH^+^ and TH- neurons on one side of the SNpc.  **Stereology conducted blinded to dose group?** Not stated | **TH^+^ Neurons in the SNpc (Fig. 2)**  **C57BL/6J Male Mice:** The mean number of TH^+^ neurons in the SNpc was reduced by ~ 9% and 26% in the 1 and 5 mg/kg PQ groups, respectively. The reduction in the 1 mg/kg group was not statistically significant.  **DBA/2J Male Mice:** The mean number of TH^+^ neurons in the SNpc was reduced by ~ 4% and 15% in the 1 and 5 mg/kg PQ groups, respectively. The reduction in the 1 mg/kg was not statistically significant.  **C57BL/6J and BBA/2J Females** the data was not shown for females but authors stated that the number of TH^+^ neurons was significantly reduced in C57BL/6J and DBA/2J females at a PQ dose of 5 mg/kg but not at a dose of 1 mg/kg.  **TH Negative (Nissl-Stained) Neurons in the SNpc**  The authors stated that a statistically significant reduction in the number of TH^−^ neurons was observed in DBA2J mice administered 1 mg/kg and 5 mg/kg PQ, but not in the C57BL/6J mice. No stereological TH^−^ data were provided. |
| Zhou (2011) [57]  **Study #50** | **Strain:** B6 males with tamoxifen-inducible CreERT males mated with PINK1-RNAi females.  **Source**: Jackson Laboratories  **Gender:** Male  **Ages:** 2 or 12 weeks  **Quarantine Period:** Not provided  **Housing:** Not provided    **Number of PQ Mice**  **2 Weeks Old:** 7 to 8 mice  **12 Weeks Old:** 5 mice | **PQ dichloride hydrate**  **Source:** Sigma Chemical  **PQ Doses**  **PND 14:** Single dose of 0.3 mg/kg i.p.  **12 Week-Old Mice:** 10 mg/kg i.p. twice weekly for 3 weeks  **Negative Control:** Saline  **Sacrifice:** Mice decapitated   1. 1 week after PND 14 2. 1 week after the last dose commencing on week 12 3. 20 months of age in subsets a and b.   **Analytical confirmation of dose concentration in dosing solutions**: Not stated | **Tissue Collection and Processing: SNpc**  Mice were decapitated at time intervals a, b, or c; the hindbrain was fixed, frozen and cut 40 μm sections through the striatum were immuno-stained for TH^+^ neurons (DAB chromogen).  **3D Stereology: TH^+^ Neurons in SNpc**  Methodological details were not presented in this manuscript. The description provided above was taken from Zhou et al. (2007) [58] and Xia et al. (2001) [59].  The number of sections, guard z1, disector h8, and other details of the optical fractionator technique were not provided in this publication.  **Stereology conducted blinded to dose group?** Not stated | **TH^+^ Neurons in the SNpc (Fig. 1 Sacrifice Interval a or b)**  The mean number of TH^+^ neurons in the SNpc was significantly reduced (~18%) 1 week after a single dose of PQ (0.3 mg/kg) administered to transgenic male mice (a).  The mean number of TH^+^ neurons in the SNpc was significantly reduced (~14%) 1 week after the last PQ dose (10 mg/kg twice weekly for 3 weeks). The magnitude of the reduction (~28%) was significantly greater is mice that received PQ on PND 14 and then twice weekly for 3 weeks commencing on week 12.  **TH^+^ Neurons in the SNpc (Fig. 4 Sacrifice Interval c)**  The mean number of TH*^+^* neurons was significantly reduced (20%) in male mice administered PQ on PND 14 (0.3 mg/kg) and week 12 (10 mg/kg. twice weekly for 3 weeks) and sacrificed at 20 months of age.  **Overall Assessment:** The absence of methodological details in Zhou et al. (2011) [57], limited the utility of this study. |

^1^Data on the effect of an interaction between paraquat and other agents (e.g. maneb, nanoparticles) have not been summarized in this table because these treatments were not evaluated in our study. For the same reason, results on the behavioral effects of paraquat are not summarized here. Neurochemistry data are not summarized because neurochemical endpoints were not evaluated in the study by Smeyne (2016 – current study). Previous studies from our laboratories (Breckenridge et al 2013 [2], Minnema et al., 2014 [33] have conducted a comprehensive evaluation of the effects of MPTP and PQ on neurochemical endpoints. The study number in column 2 refers to the study number in the Tables of S3 Appendix.

**REFERENCES**

1. Barlow BK, Richfield EK, Cory-Slechta DA, Thiruchelvam M. A fetal risk factor for Parkinson's disease. Developmental Neuroscience. 2004;26(1):11-23. Epub 2004/10/29. doi: DNE2004026001011 [pii]

10.1159/000080707 [doi]. PubMed PMID: 15509894.

2. Breckenridge CB, Sturgess NC, Butt M, Wolf JC, Zadory D, Beck M, et al. Pharmacokinetic, neurochemical, stereological and neuropathological studies on the potential effects of paraquat in the substantia nigra pars compacta and striatum of male C57BL/6J mice. Neurotoxicology. 2013;37:1-14. Epub 2013/03/26. doi: 10.1016/j.neuro.2013.03.005. PubMed PMID: 23523781.

3. Brooks AI, Chadwick CA, Gelbard HA, Cory-Slechta DA, Federoff HJ. Paraquat elicited neurobehavioral syndrome caused by dopaminergic neuron loss. Brain Res. 1999;823(1-2):1-10.

4. Chen P, Chen Z, Li A, Lou XC, Wu XK, Zhao CJ, et al. Catalytic metalloporphyrin protects against paraquat neurotoxicity in vivo. Biomedical and Environmental Sciences. 2008;21(3):233-8. Epub 2008/08/22. doi: 10.1016/s0895-3988(08)60035-5. PubMed PMID: 18714822.

5. Kato H, Araki T, Imai Y, Takahashi A, Itoyama Y. Protection of dopaminergic neurons with a novel astrocyte modulating agent (R)-(-)-2-propyloctanoic acid (ONO-2506) in an MPTP-mouse model of Parkinson's disease. J Neurol Sci. 2003;208(1-2):9-15. Epub 2003/03/18. PubMed PMID: 12639719.

6. Choi HS, An JJ, Kim SY, Lee SH, Kim DW, Yoo KY, et al. PEP-1-SOD fusion protein efficiently protects against paraquat-induced dopaminergic neuron damage in a Parkinson disease mouse model. Free Radical Biology and Medicine. 2006;41(7):1058-68. Epub 2006/09/12. doi: S0891-5849(06)00390-X [pii]

10.1016/j.freeradbiomed.2006.06.006 [doi]. PubMed PMID: 16962931.

7. Manning-Bog AB, McCormack AL, Li J, Uversky VN, Fink AL, Di Monte DA. The herbicide paraquat causes up-regulation and aggregation of alpha-synuclein in mice: paraquat and alpha-synuclein. J Biol Chem. 2002;277(3):1641-4. Epub 2001/11/15. doi: 10.1074/jbc.C100560200. PubMed PMID: 11707429.

8. Manning-Bog AB, McCormack AL, Purisai MG, Bolin LM, Di Monte DA. Alpha-synuclein overexpression protects against paraquat-induced neurodegeneration. Journal of Neuroscience. 2003;23(8):3095-9. Epub 2003/04/30. doi: 23/8/3095 [pii]. PubMed PMID: 12716914.

9. Choi HS, Lee SH, Kim SY, An JJ, Hwang SI, Kim DW, et al. Transduced Tat-alpha-synuclein protects against oxidative stress in vitro and in vivo. J Biochem Mol Biol. 2006;39(3):253-62. Epub 2006/06/08. PubMed PMID: 16756753.

10. Choi WS, Abel G, Klintworth H, Flavell RA, Xia Z. JNK3 Mediates Paraquat- and Rotenone-Induced Dopaminergic Neuron Death. Journal of Neuropathology and Experimental Neurology. 2010;69(5):511-20. Epub 2010/04/27. doi: 10.1097/NEN.0b013e3181db8100. PubMed PMID: 20418776.

11. Cristovao AC, Choi D, Baltazar G, Beal F, Kim YS. The role of NADPH oxidase 1-derived reactive oxygen species in paraquat-mediated dopaminergic cell death. Antioxidants and Redox Signaling. 2009;11(9):2105-18. Epub 2009/05/20. doi: 10.1089/ARS.2009.2459 [doi]. PubMed PMID: 19450058.

12. Fei Q, McCormack AL, Di Monte DA, Ethell DW. Paraquat neurotoxicity is mediated by a Bak-dependent mechanism. J Biol Chem. 2008;283(6):3357-64. Epub 2007/12/07. doi: 10.1074/jbc.M708451200. PubMed PMID: 18056701.

13. Fernagut PO, Hutson CB, Fleming SM, Tetreaut NA, Salcedo J, Masliah E, et al. Behavioral and histopathological consequences of paraquat intoxication in mice: effects of alpha-synuclein over-expression. Synapse (New York, NY). 2007;61(12):991-1001. Epub 2007/09/20. doi: 10.1002/syn.20456. PubMed PMID: 17879265; PubMed Central PMCID: PMCPMC3097512.

14. Gollamudi S, Johri A, Calingasan NY, Yang L, Elemento O, Beal MF. Concordant signaling pathways produced by pesticide exposure in mice correspond to pathways identified in human Parkinson's disease. PLoS One. 2012;7(5):e36191. Epub 2012/05/09. doi: 10.1371/journal.pone.0036191. PubMed PMID: 22563483; PubMed Central PMCID: PMC3341364.

15. Jiao Y, Lu L, Williams RW, Smeyne RJ. Genetic dissection of strain dependent paraquat-induced neurodegeneration in the substantia nigra pars compacta. PLoS One. 2012;7(1):e29447. Epub 2012/02/01. doi: 10.1371/journal.pone.0029447. PubMed PMID: 22291891; PubMed Central PMCID: PMC3265472.

16. Kang MJ, Gil SJ, Koh HC. Paraquat induces alternation of the dopamine catabolic pathways and glutathione levels in the substantia nigra of mice. Toxicology Letters. 2009;188(2):148-52. Epub 2009/05/19. doi: 10.1016/j.toxlet.2009.03.026. PubMed PMID: 19446248.

17. Kang MJ, Gil SJ, Koh HC. Paraquat induces alternation of the dopamine catabolic pathways and glutathione levels in the substantia nigra of mice. Toxicol Lett. 2009;188(2):148-52. Epub 2009/05/19. doi: S0378-4274(09)00183-0 [pii]

10.1016/j.toxlet.2009.03.026 [doi]. PubMed PMID: 19446248.

18. Kang MJ, Gil SJ, Lee JE, Koh HC. Selective vulnerability of the striatal subregions of C57BL/6 mice to paraquat. Toxicology Letters. 2010;195(2-3):127-34. Epub 2010/03/24. doi: 10.1016/j.toxlet.2010.03.011. PubMed PMID: 20307631.

19. Khwaja M, McCormack A, McIntosh JM, Di Monte DA, Quik M. Nicotine partially protects against paraquat-induced nigrostriatal damage in mice; link to alpha6beta2* nAChRs. Journal of Neurochemistry. 2007;100(1):180-90. Epub 2007/01/18. doi: JNC4177 [pii]

10.1111/j.1471-4159.2006.04177.x [doi]. PubMed PMID: 17227438.

20. Li X, Yin J, Cheng CM, Sun JL, Li Z, Wu YL. Paraquat induces selective dopaminergic nigrostriatal degeneration in aging C57BL/6 mice. Chinese Medical Journal (English Edition). 2005;118(16):1357-61. Epub 2005/09/15. PubMed PMID: 16157030.

21. Li H, Wu S, Wang Z, Lin W, Zhang C, Huang B. Neuroprotective effects of tert-butylhydroquinone on paraquat-induced dopaminergic cell degeneration in C57BL/6 mice and in PC12 cells. Archives of Toxicology. 2012;86(11):1729-40. Epub 2012/09/18. doi: 10.1007/s00204-012-0935-y. PubMed PMID: 22983789.

22. Mangano EN, Hayley S. Inflammatory priming of the substantia nigra influences the impact of later paraquat exposure: Neuroimmune sensitization of neurodegeneration. Neurobiol Aging. 2009;30(9):1361-78. Epub 2008/01/12. doi: S0197-4580(07)00449-6 [pii]

10.1016/j.neurobiolaging.2007.11.020 [doi]. PubMed PMID: 18187236.

23. Mangano EN, Peters S, Litteljohn D, So R, Bethune C, Bobyn J, et al. Granulocyte macrophage-colony stimulating factor protects against substantia nigra dopaminergic cell loss in an environmental toxin model of Parkinson's disease. Neurobiol Dis. 2011;43(1):99-112. Epub 2011/03/08. doi: 10.1016/j.nbd.2011.02.011. PubMed PMID: 21377529.

24. Mangano EN, Litteljohn D, So R, Nelson E, Peters S, Bethune C, et al. Interferon-gamma plays a role in paraquat-induced neurodegeneration involving oxidative and proinflammatory pathways. Neurobiology of Aging. 2012;33(7):1411-26. Epub 2011/04/13. doi: 10.1016/j.neurobiolaging.2011.02.016. PubMed PMID: 21482445.

25. Matsuoka Y, Vila M, Lincoln S, McCormack A, Picciano M, LaFrancois J, et al. Lack of nigral pathology in transgenic mice expressing human alpha-synuclein driven by the tyrosine hydroxylase promoter. Neurobiol Dis. 2001;8(3):535-9. Epub 2001/07/10. doi: 10.1006/nbdi.2001.0392. PubMed PMID: 11442360.

26. McCormack AL, Thiruchelvam M, Manning-Bog AB, Thiffault C, Langston JW, Cory-Slechta DA, et al. Environmental risk factors and Parkinson's disease: Selective degeneration of nigral dopaminergic neurons caused by the herbicide paraquat. Neurobiology of Disease. 2002;10(2):119-27.

27. Chan P, Di Monte DA, Langston JW, Janson AM. (+)MK-801 does not prevent MPTP-induced loss of nigral neurons in mice. J Pharmacol Exp Ther. 1997;280(1):439-46. Epub 1997/01/01. PubMed PMID: 8996226.

28. McCormack AL, Di Monte DA. Effects of L-dopa and other amino acids against paraquat-induced nigrostriatal degeneration. Journal of Neurochemistry. 2003;85(1):82-6. Epub 2003/03/19. PubMed PMID: 12641729.

29. McCormack AL, Atienza JG, Johnston LC, Andersen JK, Vu S, Di Monte DA. Role of oxidative stress in paraquat-induced dopaminergic cell degeneration. Journal of Neurochemistry. 2005;93(4):1030-7. PubMed PMID: 15857406.

30. Kaur D, Yantiri F, Rajagopalan S, Kumar J, Mo JQ, Boonplueang R, et al. Genetic or pharmacological iron chelation prevents MPTP-induced neurotoxicity in vivo: a novel therapy for Parkinson's disease. Neuron. 2003;37(6):899-909. Epub 2003/04/03. PubMed PMID: 12670420.

31. McCormack AL, Thiruchelvam M, Manning-Bog AB, Thiffault C, Langston JW, Cory-Slechta DA, et al. Environmental risk factors and Parkinson's disease: selective degeneration of nigral dopaminergic neurons caused by the herbicide paraquat. Neurobiol Dis. 2002;10(2):119-27. Epub 2002/07/20. doi: S0969996102905073 [pii]. PubMed PMID: 12127150.

32. McCormack AL, Atienza JG, Langston JW, Di Monte DA. Decreased susceptibility to oxidative stress underlies the resistance of specific dopaminergic cell populations to paraquat-induced degeneration. Neuroscience. 2006;141(2):929-37. Epub 2006/05/09. doi: 10.1016/j.neuroscience.2006.03.069. PubMed PMID: 16677770.

33. Minnema DJ, Travis KZ, Breckenridge CB, Sturgess NC, Butt M, Wolf JC, et al. Dietary administration of paraquat for 13weeks does not result in a loss of dopaminergic neurons in the substantia nigra of C57BL/6J mice. Regulatory Toxicology and Pharmacology. 2014;68(2):250-8. Epub 2014/01/07. doi: 10.1016/j.yrtph.2013.12.010. PubMed PMID: 24389362.

34. Mitra S, Chakrabarti N, Bhattacharyya A. Differential regional expression patterns of alpha-synuclein, TNF-alpha, and IL-1beta; and variable status of dopaminergic neurotoxicity in mouse brain after Paraquat treatment. J Neuroinflammation. 2011;8(1):163. Epub 2011/11/25. doi: 10.1186/1742-2094-8-163. PubMed PMID: 22112368.

35. Pabon MM, Bachstetter AD, Hudson CE, Gemma C, Bickford PC. CX3CL1 reduces neurotoxicity and microglial activation in a rat model of Parkinson's disease. J Neuroinflammation. 2011;8:9. Epub 2011/01/27. doi: 10.1186/1742-2094-8-9. PubMed PMID: 21266082; PubMed Central PMCID: PMC3039584.

36. Norris EH, Uryu K, Leight S, Giasson BI, Trojanowski JQ, Lee VM. Pesticide exposure exacerbates alpha-synucleinopathy in an A53T transgenic mouse model. American Journal Of Pathology. 2007;170(2):658-66. Epub 2007/01/27. doi: 170/2/658 [pii]

10.2353/ajpath.2007.060359 [doi]. PubMed PMID: 17255333.

37. Peng J, Mao XO, Stevenson FF, Hsu M, Andersen JK. The herbicide paraquat induces dopaminergic nigral apoptosis through sustained activation of the JNK pathway. J Biol Chem. 2004;279(31):32626-32. Epub 2004 May 20.

38. Peng J, Stevenson FF, Doctrow SR, Andersen JK. Superoxide dismutase/catalase mimetics are neuroprotective against selective paraquat-mediated dopaminergic neuron death in the substantial nigra: implications for Parkinson disease. J Biol Chem. 2005;280(32):29194-8. Epub 2005/06/11. doi: M500984200 [pii]

10.1074/jbc.M500984200 [doi]. PubMed PMID: 15946937.

39. Peng J, Peng L, Stevenson FF, Doctrow SR, Andersen JK. Iron and paraquat as synergistic environmental risk factors in sporadic Parkinson's disease accelerate age-related neurodegeneration. Journal of Neuroscience. 2007;27(26):6914-22. Epub 2007/06/29. doi: 27/26/6914 [pii]

10.1523/JNEUROSCI.1569-07.2007 [doi]. PubMed PMID: 17596439.

40. Peng J, Stevenson FF, Oo ML, Andersen JK. Iron-enhanced paraquat-mediated dopaminergic cell death due to increased oxidative stress as a consequence of microglial activation. Free Radical Biology and Medicine. 2009;46(2):312-20. Epub 2008/11/26. doi: S0891-5849(08)00663-1 [pii]

10.1016/j.freeradbiomed.2008.10.045 [doi]. PubMed PMID: 19027846.

41. Peng J, Oo ML, Andersen JK. Synergistic effects of environmental risk factors and gene mutations in Parkinson's disease accelerate age-related neurodegeneration. Journal of Neurochemistry. 2010;115(6):1363-73. Epub 2010/11/03. doi: 10.1111/j.1471-4159.2010.07036.x. PubMed PMID: 21039522.

42. Prakash J, Yadav SK, Chouhan S, Singh SP. Neuroprotective Role of Withania somnifera Root Extract in Maneb-Paraquat Induced Mouse Model of Parkinsonism. Neurochemical Research. 2013;38(5):972-80. Epub 2013/02/23. doi: 10.1007/s11064-013-1005-4. PubMed PMID: 23430469.

43. Prasad K, Tarasewicz E, Mathew J, Strickland PA, Buckley B, Richardson JR, et al. Toxicokinetics and toxicodynamics of paraquat accumulation in mouse brain. Experimental Neurology. 2009;215(2):358-67. Epub 2008/12/17. doi: S0014-4886(08)00429-9 [pii]

10.1016/j.expneurol.2008.11.003 [doi]. PubMed PMID: 19084006.

44. Purisai MG, McCormack AL, Cumine S, Li J, Isla MZ, Di Monte DA. Microglial activation as a priming event leading to paraquat-induced dopaminergic cell degeneration. Neurobiol Dis. 2007;25(2):392-400. PubMed PMID: 17166727.

45. Rappold PM, Cui M, Chesser AS, Tibbett J, Grima JC, Duan L, et al. Paraquat neurotoxicity is mediated by the dopamine transporter and organic cation transporter-3. Proc Natl Acad Sci U S A. 2011;108(51):20766-71. Epub 2011/12/07. doi: 10.1073/pnas.1115141108. PubMed PMID: 22143804; PubMed Central PMCID: PMCPmc3251116.

46. Reeves R, Thiruchelvam M, Baggs RB, Cory-Slechta DA. Interactions of paraquat and triadimefon: behavioral and neurochemical effects. Neurotoxicology. 2003;24(6):839-50. Epub 2003/11/26. doi: 10.1016/s0161-813x(03)00057-3. PubMed PMID: 14637379.

47. Ren JP, Zhao YW, Sun XJ. Toxic influence of chronic oral administration of paraquat on nigrostriatal dopaminergic neurons in C57BL/6 mice Chinese Medical Journal (English Edition). 2009;122(19):2366-71.

48. Rojo AI, Cavada C, de Sagarra MR, Cuadrado A. Chronic inhalation of rotenone or paraquat does not induce Parkinson's disease symptoms in mice or rats. Experimental Neurology. 2007;208(1):120-6. Epub 2007/09/21. doi: S0014-4886(07)00300-7 [pii]

10.1016/j.expneurol.2007.07.022 [doi]. PubMed PMID: 17880941.

49. Srivastava G, Dixit A, Yadav S, Patel DK, Prakash O, Singh MP. Resveratrol potentiates cytochrome P450 2d22-mediated neuroprotection in maneb- and paraquat-induced parkinsonism in the mouse. Free Radical Biology and Medicine. 2012;52(8):1294-306. Epub 2012/02/16. doi: 10.1016/j.freeradbiomed.2012.02.005. PubMed PMID: 22334051.

50. Su C, Niu P. Low doses of single or combined agrichemicals induces alpha-synuclein aggregation in nigrostriatal system of mice through inhibition of proteasomal and autophagic pathways. Int J Clin Exp Med. 2015;8(11):20508-15. Epub 2016/02/18. PubMed PMID: 26884967; PubMed Central PMCID: PMC4723812.

51. Thiruchelvam M, Brockel BJ, Richfield EK, Baggs RB, Cory-Slechta DA. Potentiated and preferential effects of combined paraquat and maneb on nigrostriatal dopamine systems: environmental risk factors for Parkinson's disease? Brain Res. 2000;873(2):225-34. Epub 2000/08/10. doi: S0006-8993(00)02496-3 [pii]. PubMed PMID: 10930548.

52. Thiruchelvam M, Richfield EK, Baggs RB, Tank AW, Cory-Slechta DA. The nigrostriatal dopaminergic system as a preferential target of repeated exposures to combined paraquat and maneb: implications for Parkinson's disease. Journal of Neuroscience. 2000;20(24):9207-14.

53. Thiruchelvam M, Richfield EK, Goodman BM, Baggs RB, Cory-Slechta DA. Developmental exposure to the pesticides paraquat and maneb and the Parkinson's disease phenotype. Neurotoxicology. 2002;23(4-5):621-33. Epub 2002/11/14. doi: S0161-813X(02)00092-X [pii]. PubMed PMID: 12428734.

54. Thiruchelvam M, McCormack A, Richfield EK, Baggs RB, Tank AW, Di Monte DA, et al. Age-related irreversible progressive nigrostriatal dopaminergic neurotoxicity in the paraquat and maneb model of the Parkinson's disease phenotype. Eur J Neurosci. 2003;18(3):589-600. Epub 2003/08/13. doi: 2781 [pii]. PubMed PMID: 12911755.

55. Watson MB, Nobuta H, Abad C, Lee SK, Bala N, Zhu C, et al. PACAP deficiency sensitizes nigrostriatal dopaminergic neurons to paraquat-induced damage and modulates central and peripheral inflammatory activation in mice. Neuroscience. 2013;240:277-86. Epub 2013/03/19. doi: 10.1016/j.neuroscience.2013.03.002. PubMed PMID: 23500093; PubMed Central PMCID: PMCPMC3637876.

56. Yin L, Lu L, Prasad K, Richfield EK, Unger EL, Xu J, et al. Genetic-based, differential susceptibility to paraquat neurotoxicity in mice. Neurotoxicology and Teratology. 2011;33(3):415-21. Epub 2011/03/05. doi: 10.1016/j.ntt.2011.02.012. PubMed PMID: 21371552.

57. Zhou H, Huang C, Tong J, Xia XG. Early Exposure to Paraquat Sensitizes Dopaminergic Neurons to Subsequent Silencing of PINK1 Gene Expression in Mice. Int J Biol Sci. 2011;7(8):1180-7. Epub 2011/11/02. PubMed PMID: 22043175; PubMed Central PMCID: PMC3204408.

58. Zhou H, Falkenburger BH, Schulz JB, Tieu K, Xu Z, Xia XG. Silencing of the Pink1 gene expression by conditional RNAi does not induce dopaminergic neuron death in mice. Int J Biol Sci. 2007;3(4):242-50. Epub 2007/03/29. PubMed PMID: 17389931; PubMed Central PMCID: PMC1820878.

59. Xia XG, Harding T, Weller M, Bieneman A, Uney JB, Schulz JB. Gene transfer of the JNK interacting protein-1 protects dopaminergic neurons in the MPTP model of Parkinson's disease. Proc Natl Acad Sci U S A. 2001;98(18):10433-8. Epub 2001/08/16. doi: 10.1073/pnas.181182298. PubMed PMID: 11504916; PubMed Central PMCID: PMC56978.
