# Supplementary figures and images for: Assessment of the Effects of MPTP and Paraquat on Dopaminergic Neurons and Microglia in the Substantia Nigra Pars Compacta of C57BL/6 Mice
Source: PLoS One. 2016 Oct 27;11(10):e0164094. doi: 10.1371/journal.pone.0164094 (PMC5082881; doi:10.1371/journal.pone.0164094)

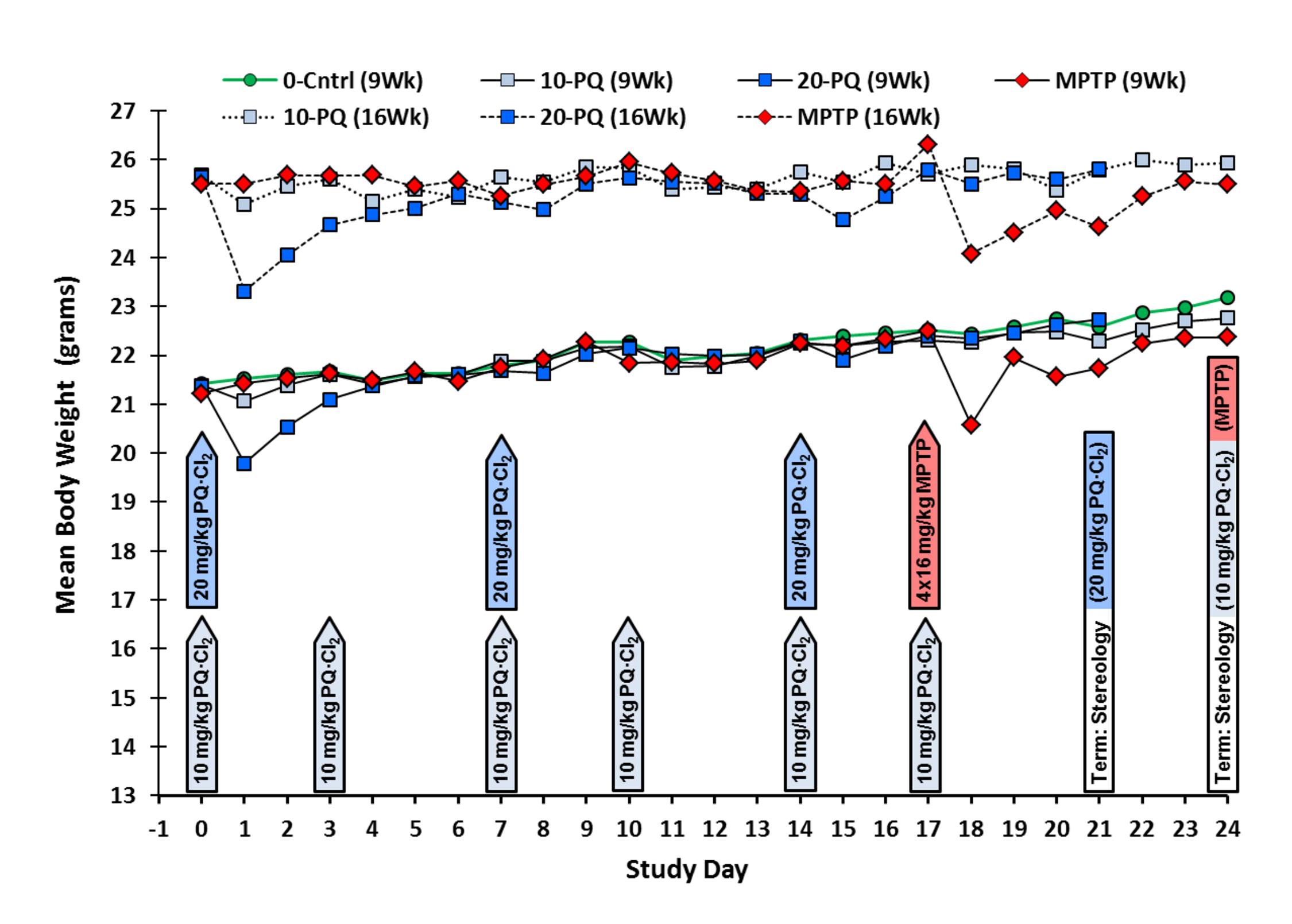

Supplement: S1 Fig — Mice were either 9 weeks of age (9Wk, solid lines) or 16 weeks of age (16Wk, dashed lines) at the time of treatment initiation on Study Day 1. Mice receiving 10 mg/kg/dose PQ·Cl2 were injected with the test item formulation on Study Days 0, 3, 7, 10, 14 and 17, and then euthanized at 8, 16, 24, 48, 96 and 168 hours after the final dose (neuropathology) or on Study Day 24 (stereology). Mice receiving 20 mg/kg/dose PQ·Cl2 were injected with the test item formulation on Study Days 0, 7 and 14, and then euthanized at 8, 16, 24, 48 96 and 168 hours after the final dose (neuropathology) or on Study Day 21 (stereology). MPTP mice received four injections of MPTP (16 mg/kg/dose; expressed as free base) at 2-hour intervals on Study Day 17, and then euthanized on either Study Day 19 (48 hours after the final dose; neuropathology) or Study Day 24 (stereology). Control mice were administered saline vehicle ip on the same days as the 10 mg/kg/dose PQ·Cl2 mice. Body weights were measured and recorded daily. (TIF) [file pone.0164094.s005.tif]
