# Supplementary material for: Assessment of the Effects of MPTP and Paraquat on Dopaminergic Neurons and Microglia in the Substantia Nigra Pars Compacta of C57BL/6 Mice
Source: PLoS One. 2016 Oct 27;11(10):e0164094. doi: 10.1371/journal.pone.0164094 (PMC5082881; doi:10.1371/journal.pone.0164094)
Supplement: S1 Table — (DOCX) [file pone.0164094.s006.docx]

**Supplemental Table S1**: Statistical comparisons of the effect of animal supply, age, housing, paraquat dose frequency or stereological method on the number of TH^+^ neurons in control, paraquat or MPTP treated groups of mice.

| Characteristic | Level A | Level B | Level A | Level B | Statistical Comparisons Between Groups | | |
| --- | --- | --- | --- | --- | --- | --- | --- |
|  |  |  |  |  | Level A Mean ± SEM | Level B Mean ± SEM | p-value A < B or A > B |
| Animal Supplier | Harlan | Jackson | G4 - Control | G5 - Control | 6679 ±181 | 6531 ±184 | 0.5722 |
|  |  |  | G4 - PQ | G5 - PQ | 6523 ±161 | 7018 ±246 | 0.1034 |
|  |  |  | G4 - MPTP | G5 - MPTP | 3601 ±394 | 4425 ±374 | 0.1444 |
|  | | | | | | | |
| Animal  Age | 9 Weeks | 16 Weeks | G2 - Control | G3 - Control | (G2 Control = G3 Control) | | |
|  |  |  | G2 - PQ | G3 - PQ | 6291 ±241 | 5969 ±300 | 0.4109 |
|  |  |  | G2 - MPTP | G3 - MPTP | 4162 ±296 | 3689 ±378 | 0.3399 |
|  | | | | | | | |
| Animal Housing | WIL | SJCRH | G3 - Control | G5 - Control | 6302 ±262 | 6531 ±184 | 0.4810 |
|  |  |  | G3 - PQ | G5 - PQ | 5969 ±300 | 7018 ±246 | 0.0124 |
|  |  |  | G3 - MPTP | G5 - MPTP | 3689 ±378 | 4425 ±374 | 0.1852 |
|  | | | | | | | |
| Dose Frequency | PQ 20 mg/kg Weekly | PQ 10 mg/kg Biweekly | G2 - Weekly | G2- Biweekly | 6291 ±241 | 6114 ±198 | 0.5762 |
|  |  |  | G3 - Weekly | G3- Biweekly | 5968 ±300 | 6294 ±238 | 0.4039 |
|  | | | | | | | |
| Stereological Method | EPL | SJCRH | G1 - Control | G2 - Control | 6861 ±338 | 6302 ±262 | 0.2006 |
|  |  |  | G1 - PQ | G2 - PQ | 6679 ±369 | 6291 ±241 | 0.3863 |
|  |  |  | G1 - MPTP | G2 - MPTP | 4123 ± 375 | 4162 ±296 | 0.9361 |
