# Supplementary material for: Assessment of the Effects of MPTP and Paraquat on Dopaminergic Neurons and Microglia in the Substantia Nigra Pars Compacta of C57BL/6 Mice
Source: PLoS One. 2016 Oct 27;11(10):e0164094. doi: 10.1371/journal.pone.0164094 (PMC5082881; doi:10.1371/journal.pone.0164094)
Supplement: S2 Table — (DOCX) [file pone.0164094.s007.docx]

Supplemental Table S2: Individual Animal Neuropathology Severity Grade in the SNpc of 9 Week Old C57BL/6J Mice (WIL Study): 8 Hours Post-Dosing

| Animal | Group 1 Vehicle Control | Group 2 Paraquat 6 x 10 mg/kg | Group 3 Paraquat 3 x 20 mg/kg |
| --- | --- | --- | --- |
| Am Cu Ag | | | |
| 1 | 0 | 0 | 0 |
| 2 | 0 | 0 | 0 |
| 3 | 0 | 0 | 0 |
| 4 | 0 | 0 | 0 |
| 5 | 0 | 0 | 0 |
| Mean | 0.00 | 0.00 | 0.00 |
| Z-score p-value |  | 0.5 | 0.5 |
| TH | | | |
| 1 | 0 | 0 | 0 |
| 2 | 0 | 0 | 0 |
| 3 | 0 | 0 | 0 |
| 4 | 1 | 0 | 0 |
| 5 | 0 | 0 | 0 |
| Mean | 0.20 | 0.00 | 0.00 |
| Z-score p-value |  | 0.84 | 0.84 |
| GFAP | | | |
| 1 | 0 | 0 | 0 |
| 2 | 0 | 0 | 0 |
| 3 | 0 | 0 | 0 |
| 4 | 0 | 0 | 0 |
| 5 | 0 | 0 | 0 |
| Mean | 0.00 | 0.00 | 0.00 |
| Z-score(p-value |  | 0.5 | 0.5 |
| Iba-1 | | | |
| 1 | 0 | 0 | 0 |
| 2 | 0 | 0 | 0 |
| 3 | 0 | 0 | 0 |
| 4 | 0 | 0 | 0 |
| 5 | 0 | 0 | 0 |
| Mean | 0.00 | 0.00 | 0.00 |
| Z-score p-value |  | 0.5 | 0.5 |

Supplemental Table S2 (Continued): Individual Animal Neuropathology Severity Grade in the SNpc of 9 Week Old C57BL/6J Mice (WIL Study): 48 Hours Post-Dosing

| Animal | Group 1  Vehicle Control | Group 2  Paraquat  6 x 10 mg/kg | Group 3  Paraquat  3 x 20 mg/kg | Group 4  MPTP  4 x16 mg/kg |
| --- | --- | --- | --- | --- |
| Am Cu Ag | | | | |
| 1 | 0 | 0 | 0 | 3 |
| 2 | 0 | 0 | 0 | 4 |
| 3 | 0 | 0 | 0 | 2 |
| 4 | 0 | 0 | 0 | 3 |
| 5 | 0 | 0 | 0 | 4 |
| Mean | 0.00 | 0.00 | 0.00 | 3.20 |
| Z-score p-value |  | 0.5 | 0.5 | <0.0001 |
| TH | | | | |
| 1 | 0 | 0 | 0 | 2 |
| 2 | 0 | 0 | 0 | 1 |
| 3 | 0 | 0 | 0 | 2 |
| 4 | 0 | 0 | 0 | 3 |
| 5 | 0 | 0 | 0 | 3 |
| Mean | 0.00 | 0.00 | 0.00 | 2.20 |
| Z-score p-value |  | 0.5 | 0.5 | <0.0001 |
| GFAP | | | | |
| 1 | 0 | 0 | 0 | 3 |
| 2 | 0 | 0 | 0 | 4 |
| 3 | 0 | 0 | 0 | 2 |
| 4 | 0 | 0 | 0 | 3 |
| 5 | 0 | 0 | 0 | 4 |
| Average | 0.00 | 0.00 | 0.00 | 3.20 |
| Z-score p-value |  | 0.5 | 0.5 | <0.0001 |
| Iba-1 | | | | |
| 1 | 0 | 0 | 0 | 3 |
| 2 | 0 | 0 | 0 | 3 |
| 3 | 0 | 0 | 0 | 3 |
| 4 | 0 | 0 | 0 | 3 |
| 5 | 0 | 0 | 0 | 2 |
| Mean | 0.00 | 0.00 | 0.00 | 2.80 |
| Z-score p-value |  | 0.5 | 0.5 | <0.0001 |

Supplemental Table S2 (Continued): Individual Animal Neuropathology Severity Grade in the SNpc of 9 Week Old C57BL/6J Mice (WIL Study): 96 Hours Post-Dose

| Animal | Group 1:  Control:  6 x 0 mg/kg | Group 2:  2X/Wk PQ  6 x 10 mg/kg | Group 3:  2X/Wk PQ  3 x 20 mg/kg |
| --- | --- | --- | --- |
| Am Cu Ag | | | |
| 1 | 0 | 0 | 0 |
| 2 | 0 | 0 | 0 |
| 3 | 0 | 0 | 0 |
| 4 | 0 | 0 | 0 |
| 5 | 0 | 0 | 0 |
| Mean | 0.00 | 0.00 | 0.00 |
| Z-score p-value |  | 0.5 | 0.5 |
| TH | | | |
| 1 | 0 | 0 | 0 |
| 2 | 0 | 0 | 0 |
| 3 | 0 | 0 | 0 |
| 4 | 0 | 0 | 0 |
| 5 | 0 | 1 | 0 |
| Mean | 0.00 | 0.20 | 0.00 |
| Z-score p-value |  | 0.16 | 0.5 |
| GFAP | | | |
| 1 | 0 | 0 | 0 |
| 2 | 0 | 0 | 0 |
| 3 | 0 | 0 | 0 |
| 4 | 0 | 0 | 0 |
| 5 | 0 | 0 | 0 |
| Mean | 0.00 | 0.00 | 0.00 |
| Z-score p-value |  | 0.5 | 0.5 |
| Iba-1 | | | |
| 1 | 0 | 0 | 0 |
| 2 | 0 | 0 | 0 |
| 3 | 0 | 0 | 0 |
| 4 | 0 | 0 | 0 |
| 5 | 0 | 0 | 0 |
| Mean | 0.00 | 0.00 | 0.00 |
| Z-score p-value |  | 0.5 | 0.5 |
